# Multilocus Inherited Neoplasia Allele Syndrome (MINAS): an update

**DOI:** 10.1038/s41431-021-01013-6

**Published:** 2022-01-04

**Authors:** Anthony McGuigan, James Whitworth, Avgi Andreou, Timothy Hearn, Genomics England Research Consortium, J. C. Ambrose, P. Arumugam, R. Bevers, M. Bleda, F. Boardman-Pretty, C. R. Boustred, H. Brittain, M. J. Caulfield, G. C. Chan, T. Fowler, A. Giess, A. Hamblin, S. Henderson, T. J. P. Hubbard, R. Jackson, L. J. Jones, D. Kasperaviciute, M. Kayikci, A. Kousathanas, L. Lahnstein, S. E. A. Leigh, I. U. S. Leong, F. J. Lopez, F. Maleady-Crowe, M. McEntagart, F. Minneci, L. Moutsianas, M. Mueller, N. Murugaesu, A. C. Need, P. O‘Donovan, C. A. Odhams, C. Patch, D. Perez-Gil, M. B. Pereira, J. Pullinger, T. Rahim, A. Rendon, T. Rogers, K. Savage, K. Sawant, R. H. Scott, A. Siddiq, A. Sieghart, S. C. Smith, A. Sosinsky, A. Stuckey, M. Tanguy, A. L. Taylor Tavares, E. R. A. Thomas, S. R. Thompson, A. Tucci, M. J. Welland, E. Williams, K. Witkowska, S. M. Wood, Marc Tischkowitz, Eamonn R. Maher

**Affiliations:** 1 Department of Medical Genetics, University of Cambridge and Cambridge University Hospitals NHS Foundation Trust, Cambridge, CB2 0QQ UK; 2 Genomics England, London, UK; 3 William Harvey Research Institute, Queen Mary University of London, London, EC1M 6BQ UK

**Keywords:** Risk factors, Cancer genetics

## Abstract

Multi-locus Inherited Neoplasia Allele Syndrome (MINAS) refers to individuals with germline pathogenic variants in two or more cancer susceptibility genes(CSGs). With increased use of exome/genome sequencing it would be predicted that detection of MINAS would become more frequent. Here we review recent progress in knowledge of MINAS. A systematic literature search for reports of individuals with germline pathogenic variants in 2 or more of 94 CSGs was performed. In addition, participants with multiple primary tumours who underwent genome sequencing as part of the Rare Disease arm of the UK 100,000 Genomes Project were interrogated to detect additional cases. We identified 385 MINAS cases (211 reported in the last 5 years, 6 from 100,000 genomes participants). Most (287/385) cases contained at least one pathogenic variant in either *BRCA1* or *BRCA2*. 108/385 MINAS cases had multiple primary tumours at presentation and a subset of cases presented unusual multiple tumour phenotypes. We conclude that, as predicted, increasing numbers of individuals with MINAS are being have been reported but, except for individuals with BRCA1/BRCA2 MINAS, individual CSG combinations are generally rare. In many cases it appears that the clinical phenotype is that which would be expected from the effects of the constituent CSG variants acting independently. However, in some instances the presence of unusual tumour phenotypes and/or multiple primary tumours suggests that there may be complex interactions between the relevant MINAS CSGs. Systematic reporting of MINAS cases in a MINAS database (e.g. https://databases.lovd.nl/shared/diseases/04296) will facilitate more accurate prognostic predictions for specific CSG combinations.

## Introduction

Though the development and progression of cancer are primarily driven by the acquisition of somatic genetic and epigenetic events that promote oncogenesis (driver mutations), in a subset of cases, cancer initiation is a consequence of a germline pathogenic variant in a high or moderate penetrance cancer susceptibility gene (CSG) [1, 2]. The proportion of a particular human cancer associated with a germline CSG pathogenic variant differs by site and tumour type being very high in some (e.g. wild-type gastrointestinal stromal tumours, paraganglioma) and low in others (e.g. lung cancer) [3–7]. Similarly, the number of CSGs that can predispose to a specific tumour type is highly variable with some only associated with a single CSG and others with many (e.g. haemangioblastoma and breast cancer respectively). Consequently, the role of, and approach to germline genetic testing in patient management varies such that for some tumour types only a fraction of cases may be tested whilst for others genetic testing can be indicated in the majority of cases. In addition, relevant testing may target a single or numerous CSGs. As advances in genomic technology have reduced the cost of genetic testing, increasing numbers of CSGs have been characterised and the tumour types associated with individual CSGs have expanded. These developments have been associated with a clear trend towards more testing with larger gene panels for many tumour types.

The incidence of pathogenic variants in CSGs in most populations is very low but in some ancestral groups, founder mutations may be as frequent as 1 in 100 individuals (e.g. BRCA1 pathogenic variants in the Ashkenazi Jewish population) [8–11]. Hence, the odds of an individual having a pathogenic variant in more than one CSG would be predicted to be remote. Nevertheless, as genetic testing expands and gene testing panels become more encompassing, increasing numbers of individuals who harbour pathogenic variants in two or more CSGs are being detected. This phenomenon, labelled MINAS (multilocus inherited neoplasia allele syndrome) was reviewed five years ago [12] and here we revisit the topic to review current knowledge on the occurrence, nature and cancer phenotypes associated with MINAS and to evaluate whether combinations of CSG mutations appear additive or synergistic with regard to cancer risks.

## Frequency of MINAS

Previously it was predicted the frequency of MINAS would increase as genetic testing of CSGs expanded [12]. To test this hypothesis a literature review for cases of MINAS using similar methodology to that adopted by Whitworth et al. [12] was performed. Additionally, the AND operator followed by the terms ‘germline mutation’ OR ‘germline’ OR ‘germ-line’ OR ‘double heterozygosity’ OR ‘double heterozygote’ OR ‘genetic predisposition’ OR ‘inherited mutation’ OR ‘MINAS’ OR ‘multilocus inherited neoplasia’ AND ‘cancer’ were used to limit the search to return a smaller number of entries by filtering out superfluous literature (see supplementary data for full details of search strategy). After candidate articles were identified, the pathogenicity of the described CSG variants was assessed to identify pathogenic or likely pathogenic (P/LP) variants (ClinVar $\ge$1* P/LP classification or classified as P/LP by American College of Clinical Genetics Guidelines). In addition to searching the published literature, cases of MINAS included in the UK 100,000 genomes data set [13] were sought among participants who were recruited to the Rare Disease arm of the study with a phenotype of ‘tumour predisposition syndromes’ and ‘multiple primary tumours’ and were considered to have MINAS if they were found to have predicted truncating variants in two CSGs (see supplementary material).

**Fig. 1:**
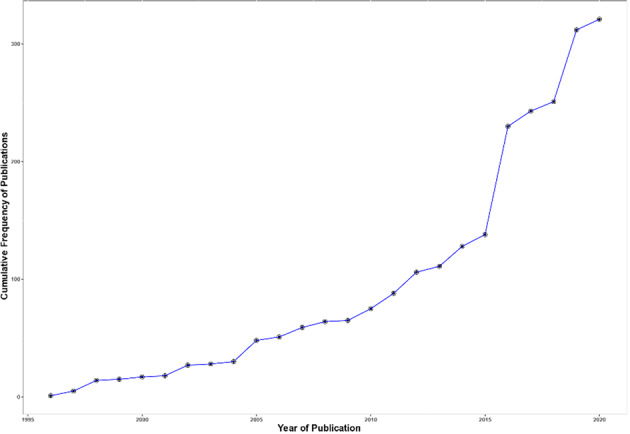
Cumulative frequency of reported individuals with MINAS cases from 1996 to 2020. (6 participants the from 100,000 genome project were included in the total for 2020 project were included in the total for 2020 figures).

In addition to the 89 cases reported by Whitworth et al. [12], 290 further MINAS cases from the literature and 6 from 100,000 Genomes Project data [13] were identified to provide a final total of 385 individuals with MINAS (see Supplementary Table 3 and additional details in Supplementary Table 4). The 385 cases were plotted according to the year of description (the six cases from 100,000 genomes project were included under 2020) and this revealed a progressive increase in the cumulative total with an apparent acceleration since 2016 (see Fig. 1).

## Genetic architecture of MINAS

**Fig. 2:**
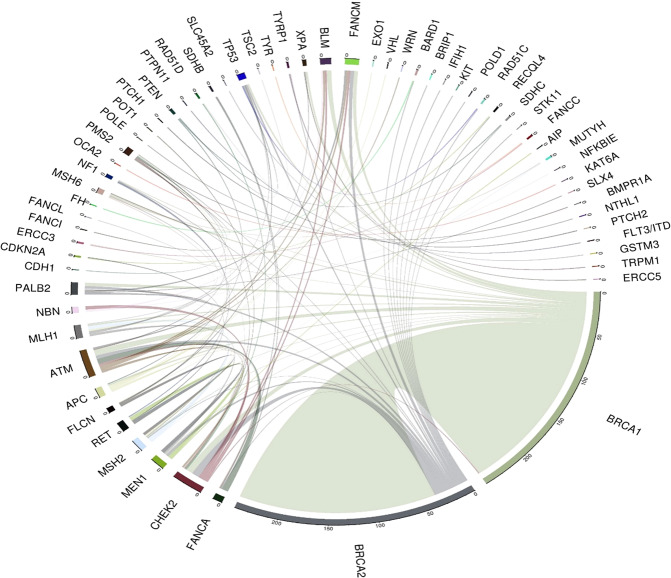
Circos plots illustrating combinations of cancer susceptibility genes (CSGs) involved in individual cases of MINAS (*n* = 385).

Reviewing the genetic architecture of the 385 individuals (current cohort) with 430 unique P/LP variants in 63 CSGs, a *BRCA1* and/or *BRCA2* variant was present in 78.5% of cases (287/385) (see Fig. 2). The current cohort was subdivided into those described previously in Whitworth et al (2016) (historical subgroup, *n* = 89) and those identified more recently (recent subgroup) and comparison of the two subgroups showed that the proportion of BRCA1/BRCA2 containing MINAS reports had increased slightly (75.3% (67/89) and 79% (220/296) respectively) (see Table 1). Comparing the MINAS-associated CSGs in the ‘historical subgroup’ and the ‘recent subgroup, revealed 45 CSGs not previously reported in MINAS cases that were present in the recent subgroup (including *ATM, CHEK2, FH*) and variants in these CSGs were present in 29% (113/385) of all cases (Table 1). Though in the historical subgroup, P/LP variants in FLCN were present in 6.7% (6/89) of cases, there were no additional cases in the ‘recent subgroup’ (0/296). The genes that were most frequently reported in recent subgroup MINAS reports, after *BRCA1* and *BRCA2*, included *CHEK2, ATM* and *FANCM*, which would be consistent with these ‘newer’ breast CSGs increasingly being included in gene testing panels for breast/ovarian cancer susceptibility [14–16]. It should be noted that not all of the genes that were included in our MINAS searching strategy (e.g*. BLM, ERCC3, ERCC5, RECQL4, XPA, FANCA, FANCC*) are known to be disease causing in a monoallelic P/LP variant state. However, on balance, we considered that these genes (marked with superscript ^a^ in Table 1) should be included in a MINAS cohort as there is the possibility that they might act as modifiers in the presence of CSG P/LP variant that was associated with a phenotype in the heterozygous state (particularly if both CSGs were functionally related e.g. implicated in DNA repair pathways). Excluding MINAS cases that included these CSGs would reduce the number MINAS cases by 32 (10 from the ‘historical subgroup’ and 22 in the ‘recent subgroup’).

**Table 1:** Frequency of involvement of individual cancer susceptibility genes (CSGs) in MINAS cases for historical subgroup (as described in Whitworth et al. [12]) and recent subgroup and all MINAS reports.

Gene	‘Historical subgroup’ MINAS frequency	‘Recent subgroup’ MINAS Frequency	Total MINAS frequency
BRCA1	60	189	249
BRCA2	62	182	244
CHEK2	0	32	32
ATM	0	31	31
MEN1	7	8	15
MLH1	5	10	15
FANCM	0	14	14
MSH2	6	8	14
PALB2	1	13	14
APC	7	4	11
BLM^a^	0	11	11
RET	6	5	11
FANCA^a^	0	10	10
PMS2	0	8	8
TP53	4	4	8
NBN	0	7	7
FLCN	6	0	6
MSH6	2	4	6
NF1	4	0	4
XPA^a^	1	3	4
PTEN	3	0	3
BARD1	0	2	2
CDKN2A	1	1	2
ERCC3^a^	0	2	2
FANCC^a^	0	2	2
MUTYH	1	1	2
RAD51C	0	2	2
RAD51D	0	2	2
RECQL4^a^	0	2	2
SDHB	0	2	2
SDHC	1	1	2
TYRP1	0	2	2
AIP	0	1	1
BMPR1A	0	1	1
BRIP1	0	1	1
CDH1	0	1	1
ERCC5^a^	0	1	1
EXO1	0	1	1
FANCI	0	1	1
FANCL	0	1	1
FH	0	1	1
FLT3/ITD	0	1	1
GSTM3	0	1	1
IFIH1	0	1	1
KAT6A	0	1	1
KIT	0	1	1
NFKBIE	0	1	1
NTHL1	0	1	1
OCA2	0	1	1
POLD1	0	1	1
POLE	0	1	1
POT1	0	1	1
PTCH1	0	1	1
PTCH2	0	1	1
PTPN11	0	1	1
SLC45A2	0	1	1
SLX4	0	1	1
STK11	0	1	1
TRPM1	0	1	1
TSC2	0	1	1
TYR	0	1	1
VHL	1	0	1
WRN	0	1	1

^a^Cancer predisposition associated with these genes occurs when there are biallelic pathogenic variants.

## Phenotypic consequences of MINAS

As discussed previously [12], it could be proposed that the adverse phenotypic consequences of MINAS could be additive (i.e. the observed cancer risks reflect those of each the relevant CSGs independent of the presence of the other) or synergistic (i.e. some CSG combinations could result in notably more severe phenotypes such as earlier ages at onset or the occurrence of tumour types that are atypical for the relevant CSGs (theoretically MINAS might also be associated with protective effects (e.g. through synthetic lethality) but this would be probably require analysis of healthy control cohorts rather than patients tested through diagnostic laboratories). To review the evidence for additive/synergistic effects, we subdivided the 385 MINAS cases in the current cohort into those with a BRCA1/BRCA2 MINAS combination (*n* = 206) and those with other combinations of CSGs and then examined possible evidence for additive/synergistic interactions. However, for assessing the occurrence of tumour types that are atypical for the relevant CSGs, for non-BRCA1/BRCA2 MINAS combinations, the number of instances of specific CSG combinations was generally very small. 108/385 (28%) MINAS cases had multiple primary tumours at presentation. Among the 108 cases, 2 (1.9%) had an unknown number of multiple primaries, 75 (69%) had had two primary tumours, 18 (17%) had had three and 13 (12%) had four or more. The most common multiple primary tumour combinations were Breast-Ovarian, with 33 cases, Breast-Breast with 24 cases and Colon-Colon with 6 cases.

### Phenotypic associations of non-BRCA1/BRCA2 MINAS

In the MINAS historical subgroup it was estimated that 14.6% of patients (13/89) had at least one tumour type that was not typical of the relevant CSGs (e.g. renal clear cell carcinoma in a patient with variants in both *BRCA1* and *MLH1*) [12]. However, in the recent cohort an atypical tumour phenotype was present in 15.8% (12/76) of non-BRCA1/BRCA2 MINAS cases (though not all studies presented individual patient-level data). Details of patients with multiple primary tumours are shown in Supplementary Table 5 and those with atypical tumour phenotypes are highlighted. Four examples of atypical MINAS phenotypes were (see Supplementary Tables 3/4 for further details) were:

1. A woman diagnosed with breast cancer and Waldenstrom’s disease aged 58 years with pathogenic variants in *BRCA1* and *BLM*. (From Sokolenko et al., [17]).
2. A woman diagnosed with breast cancer, melanoma and colorectal cancer with a pathogenic variant in FANCC and P/LP variant in TYR (Stolarova et al., [18]).
3. A woman diagnosed with lobular breast cancer at 51 years of age, followed by follicular adenoma and thyroid micropapillary carcinoma at 52 years with pathogenic variants in *PMS2* and *CDH1* (Njoroge et al., [19]).
4. A woman diagnosed with cutaneous leiomyomas at 40 years followed by colorectal polyposis at 52 years was found to be heterozygous for P/LP variants in *FH* and *BARD1* (Stradella et al., [20]).

In these cases, Waldenstrom disease, colorectal cancer, thyroid carcinoma and colorectal polyposis occurred despite not being associated with any of the relevant MINAS CSGs [21–24]. Each of these cases presented with multiple primary tumours and rare CSG combinations so it is not possible to say whether this was a manifestation of synergy between the relevant CSGs or coincidental (and the presence of multiple tumours of unusual types might have been more likely to prompt genetic testing).

### Phenotypic associations of BRCA1/BRCA2 MINAS

In a cohort of 32,295 females with *BRCA1/BRCA2* P/LP variants, Rebbeck et al. [25] identified 93 women with *BRCA1*/*BRCA2*-MINAS and reported that although there was no significant difference in the mean age at breast cancer diagnosis between *BRCA1* only pathogenic variant and the *BRCA1*/*BRCA2* MINAS women, there was an earlier age at breast cancer diagnosis (~4.5 years less) and increased incidence of ovarian cancer in women with *BRCA1*/*BRCA2* MINAS compared to *BRCA2* pathogenic variant carriers. In addition, *BRCA1*/*BRCA2* MINAS women were significantly more likely than *BRCA1* or *BRCA2* women to have had breast cancer [25]. In total, our literature review identified 206 cases of *BRCA1*/*BRCA2* MINAS and, after excluding those cases reported by Rebbeck et al. (90), the mean(+SD) age at breast cancer diagnosis was 42.4 (+-10 years, *n* = 69 compared to 40.4 years in the Rebbeck et al. *BRCA1*/*BRCA2* MINAS cohort and 41.9 (*n* = 9316) and 45.0 (*n* = 3370) in their *BRCA1*-only and *BRCA2*-only pathogenic variant carriers. These findings were consistent with the assertion in Rebbeck et al. [25] that mean age at breast cancer diagnosis on *BRCA1*/*BRCA2* MINAS is similar to that in *BRCA1* pathogenic variant carriers.

### Phenotypic associations of MINAS caused by breast cancer predisposition genes

In addition to those cases with *BRCA1*/*BRCA2* MINAS, there were also women with a pathogenic variant in *BRCA1* or *BRCA2* plus a pathogenic variant in another breast CSG. With the trend towards larger gene panels for breast cancer predisposition testing, increasing numbers of patients will be tested for *BRCA1*/*BRCA2* and moderate risk breast CSGs. Which moderate-risk breast CSGs are tested can vary between centres but a recent work by Dorling et al. recommend a hereditary breast cancer screening panel of *ATM*, *BARD1*, *BRCA1*, *BRCA2*, *CHEK2*, *PALB2*, *RAD51C*, *RAD51D* and *TP53* would contain the most clinically useful CSGs [26].

## Tumour studies and mechanisms of tumourigenesis in MINAS

When multiple primaries occur in association with MINAS and the relevant CSGs have very different tumour associations, it may be straightforward to assign the occurrence of a particular tumour to a specific CSG (notwithstanding the occurrence of atypical tumours as discussed previously). However, when the CSGs have overlapping tumour associations this is more difficult and indeed, the most frequent examples of MINAS involve multiple breast CSGs. In such cases the application of tumour studies might provide insights into whether a single CSG or multiple CSGs are implicated in the occurrence of a tumour. As most CSGs follow a tumour suppressor ‘two hit model’ of tumourigenesis the most readily available strategy for investigating mechanisms of tumourigenesis is tumour loss of heterozygosity (LOH) studies (though LOH may not be observed if the somatic inactivating event is a point mutation or promoter methylation of the wild-type allele) [27, 28]. Previously Rebbeck et al. [25] described LOH analysis from 14 informative cancers from BRCA1/BRCA2 MINAS cases and found LOH at a single locus in four tumours suggesting that in most cases the tumour develop from a second hit at a single CSG and the effects of MINAS are generally additive rather than synergistic.

When considering combinations of CSGs that might result in a synergistic effect on tumour risks, it has been suggested that mutations in CSGs that map to the same chromosome region might have a more adverse effect as LOH causing loss of a single chromosome harbouring the CSG wild type alleles would result in a tumour homozygous null for both CSGs. Other potential adverse combinations might include two CSG oncogenes, a direct relationship between the mechanisms of tumorigenesis of the two mutations (e.g. *APC* and mismatch repair gene mutations) or the CSG products being in the same cellular pathway. Other tumour profiling strategies that might provide insights into MINAS mechanisms of tumourigenesis include immunohistochemistry for CSG gene products, microsatellite instability testing and cancer mutational signature analysis [29–31]. We note that Rebbeck et al. [25] reported differences in oestrogen/progesterone receptor expression status of breast tumours in women with *BRCA1/BRCA2* MINAS compared to those with P/LP variants in *BRCA1* or *BRCA2* only such that MINAS cases were more likely to be oestrogen receptor (ER) and progesterone receptor (PR) positive breast cancers than *BRCA1*-only cases and less likely to be ER- and PR-positive than in *BRCA2*-only cases. This places the receptor phenotypes of the MINAS breast cancers intermediate between that of *BRCA2* only and *BRCA1* only breast cancers, and would be consistent with an independent rather than synergistic effect in *BRCA1/BRCA2* MINAS.

## Conclusions

Since the term MINAS was coined 5 years ago, there has been increasing awareness of the phenomenon and this is reflected in the increasing numbers of publications. There are some limitations to our analysis that would cause us to underestimate the frequency of MINAS. Firstly, only a fraction of MINAS cases is likely to be included in the published literature. Secondly, in order to reliably compare the frequency of MINAS in the current literature to that in 2016, we did not include recently identified cancer susceptibility genes. Thirdly, the cases reported to date are predominanty based on diagnostic gene-panel data and future studies based on exome or genome sequencing data would likely yield more cases of MINAS. Nevertheless, the availability of the MINAS database (https://databases.lovd.nl/shared/diseases/04296) provides a dynamic resource that enables data on published and unpublished cases to be shared widely. This is particularly important because, apart from *BRCA1/BRCA2* MINAS, other CSG MINAS combinations are rare and so clinicians faced with an individual with non-*BRCA1/BRCA2* MINAS will often find it very difficult to predict what the implications are for tumour risks in that individual. Though in most cases the evidence would appear to suggest that the effects are likely to be additive, reports of some MINAS cases with atypical tumours are a concern (though these cases may be overrepresented because of ascertainment bias). To provide the best prognostic information for individuals with MINAS, long-term follow up and molecular genetic analysis of any MINAS-related cancers (e.g. for LOH, cancer signature etc.) should be undertaken.

## Supplementary information

Supplementary Material

## Data Availability

All data generated or analysed during this study are included in this published article and its supplementary information files and in the MINAS database at https://databases.lovd.nl/shared/diseases/04296.
