# Supplementary material for: Multilocus Inherited Neoplasia Allele Syndrome (MINAS): an update
Source: Eur J Hum Genet. 2022 Jan 4;30(3):265–70. doi: 10.1038/s41431-021-01013-6 (PMC8904543; doi:10.1038/s41431-021-01013-6)

SUPPLEMENTARY MATERIAL

Multilocus Inherited Neoplasia Allele Syndrome (MINAS): An Update

Anthony McGuigan (1)*, James Whitworth (1), Avgi Andreou (1), Timothy Hearn (1), Genomics England Research Consortium (2), Marc Tischkowitz (1), Eamonn R Maher (1)*

1 Department of Medical Genetics, University of Cambridge and Cambridge University Hospitals NHS Foundation Trust, Cambridge CB2 0QQ, UK
2 Genomics England, London, UK

* Correspondence to: AMcG (aefm4@cantab.ac.uk) or EM (erm1000@medschl.cam.ac.uk)

Contents:

Supplementary Methods

1.1 Literature searching strategy

1.2 Methods for 100,000 genomes study analysis

- Table S2 –6 cases of MINAS identified through 100,000 genomes project

Supplementary Tables

Supplementary Table 1: List of 94 cancer susceptibility genes (CSGs) used for literature search.

Supplementary Table 2: Publications discovered containing MINAS Cases in Literature Review

Supplementary Table 3: Details of MINAS cases (n=296) not included in previous review of Whitworth et al (2016) (Further details for these cases and those in Whitworth et al (2016) in supplementary table 4.

Supplementary Table 4: Details of all MINAS cases included in "historical" and "recent" cohorts

Supplementary Table 5: Details of MINAS cases with multiple primary tumours. Those in which atypical tumour types were present are highlighted

Supplementary Figures

Supplementary Figure 1. Workflow for systematic literature review

Supplementary Methods

1.1 Literature Searching Strategy:

To maintain consistency with the previous report of Whitworth et al (2016), 94 well characterised CSGs frequently tested in inherited cancer gene panels were used to perform a Medline database search. Each of the 94 genes (see supplementary Table 1) were entered as a search term, separated using the OR operator to produce a list of every gene (“BRCA1” OR “BRCA2” OR “PALB2” OR ….). This list was then combined with an identical list, with the two lists being separated by the AND operator. This provided one continuous search term comprising of both lists (“BRCA1” OR “BRCA2” OR “PALB2” OR ….) AND (“BRCA1” OR “BRCA2” OR “PALB2” OR ….). This resulted in any article containing at least 2 of these 94 CSGs would be caught in the search.

Additionally the AND operator followed by the terms: “germline mutation” OR “germline” OR “germ-line” OR “double heterozygosity” OR “double heterozygote” OR “genetic predisposition” OR “inherited mutation” OR “MINAS” OR “multilocus inherited neoplasia” AND “cancer”, were used to limit the search to return a smaller number of cases by filtering out superfluous literature.

Potentially pertinent gene synonyms, as noted by the NIH Genetics Home Reference website in the ‘Other names for this gene’ section, were used in some instances to prevent missed cases due to differing naming conventions. I.e. “*CHEK2*” and “*CHK2*”.

Additional filters were used to limit the limited the search to the title and abstract. The resulting literature were then individually investigated for MINAS cases.

Following this search an additional search was made, identical to before but also including an additional filter for the term “Case Report”.

Cases at the point of discovery during the literature review were not discriminated against based on likely pathogenicity of the variants reported (see supplementary Table 2). All cases were pooled together for subsequent assessment of pathogenicity and resulting inclusion/exclusion except for cases which comprised of biallelic CSG variants. Biallelic cases were excluded as MINAS cases were defined as concurrent variants in multiple CSGs.

MINAS Literature Search Format

(“AIP” OR “ERCC2” OR “MEN1” OR “SBDS” OR “ALK” OR “ERCC3” OR “MET” OR “SDHAF2” OR “APC” OR “ERCC4” OR “MLH1” OR “SDHB” OR “ATM” OR “ERCC5” OR “MSH2” OR “SDHC” OR “BAP1” OR “EXT1” OR “MSH6” OR “SDHD” OR “BLM” OR “EXT2” OR “MUTYH” OR “SLX4” OR “BMPR1A” OR “EZH2” OR “NBN” OR “SMAD4” OR “BRCA1” OR “FANCA” OR “NF1” OR “SMARCB1” OR “BRCA2” OR “FANCB” OR “NF2” OR “STK11” OR “BUB1B” OR “FANCD2” OR “PALB2” OR “TMEM127” OR “CDC73” OR “FANCE” OR “PHOX2B” OR “TP53” OR “CDH1” OR “FANCF” OR “PMS1” OR “TSC1” OR “CDK4” OR “FANCG” OR “PMS2” OR “TSC2” OR “CDKN1C” OR “FANCI” OR “PRF1” OR “VHL” OR “CDKN2A” OR “FANCL” OR “PRKAR1A” OR “WRN” OR “CEBPA” OR “FANCM” OR “PTCH1” OR “WT1” OR “CEP57” OR “FH” OR “PTEN” OR “XPA” OR “CHEK2” OR “FLCN” OR “RAD51C” OR “XPC” OR “CYLD” OR “GATA2” OR “RAD51D” OR “DDB2” OR “GPC3” OR “RB1” OR “DICER1” OR “HNF1A” OR “RECQL4” OR “DIS3L2” OR “HRAS” OR “RET” OR “EGFR” OR “KIT” OR “RHBDF2” OR “EPCAM” OR “MAX” OR “RUNX1” OR “CHK1” OR “CHK2” OR “XPD” OR “MMAC1” OR “TEP-1” OR “RAD5*”)

AND

(“AIP” OR “ERCC2” OR “MEN1” OR “SBDS” OR “ALK” OR “ERCC3” OR “MET” OR “SDHAF2” OR “APC” OR “ERCC4” OR “MLH1” OR “SDHB” OR “ATM” OR “ERCC5” OR “MSH2” OR “SDHC” OR “BAP1” OR “EXT1” OR “MSH6” OR “SDHD” OR “BLM” OR “EXT2” OR “MUTYH” OR “SLX4” OR “BMPR1A” OR “EZH2” OR “NBN” OR “SMAD4” OR “BRCA1” OR “FANCA” OR “NF1” OR “SMARCB1” OR “BRCA2” OR “FANCB” OR “NF2” OR “STK11” OR “BUB1B” OR “FANCD2” OR “PALB2” OR “TMEM127” OR “CDC73” OR “FANCE” OR “PHOX2B” OR “TP53” OR “CDH1” OR “FANCF” OR “PMS1” OR “TSC1” OR “CDK4” OR “FANCG” OR “PMS2” OR “TSC2” OR “CDKN1C” OR “FANCI” OR “PRF1” OR “VHL” OR “CDKN2A” OR “FANCL” OR “PRKAR1A” OR “WRN” OR “CEBPA” OR “FANCM” OR “PTCH1” OR “WT1” OR “CEP57” OR “FH” OR “PTEN” OR “XPA” OR “CHEK2” OR “FLCN” OR “RAD51C” OR “XPC” OR “CYLD” OR “GATA2” OR “RAD51D” OR “DDB2” OR “GPC3” OR “RB1” OR “DICER1” OR “HNF1A” OR “RECQL4” OR “DIS3L2” OR “HRAS” OR “RET” OR “EGFR” OR “KIT” OR “RHBDF2” OR “EPCAM” OR “MAX” OR “RUNX1” OR “CHK1” OR “CHK2” OR “XPD” OR “MMAC1” OR “TEP-1” OR “RAD5*”)

AND

“germline mutation” OR “germline” OR “germ-line” OR “double heterozygosity” OR “double heterozygote” OR “genetic predisposition” OR “inherited mutation” OR “MINAS” OR “multilocus inherited neoplasia” AND “cancer”

A schematic representation of the literature searching workflow is provided in supplementary Figure 1

1.2 R Script (version 3.6.3) for identifying MINAS cases within the 100,000 genomes project data set

```
attach(tiering_data_2020.07.01_12.11.45)

## Vector of THE 94 Illumina Oncotarget genes + The tier1/2 genes previously found in 100k MINAS ("FGD1", "SDHA", "POLD1", "CDKN1B")

Oncotarget <- c("AIP", "ALK", "APC", "ATM", "BAP1", "BLM", "BMPR1A", "BRCA1", "BRCA2", "BRIP1", "BUB1B", "CDC73", "CDH1", "CDK4", "CDKN1C",
"CDKN2A", "CEBPA", "CEP57", "CHEK2", "CYLD", "DDB2", "DICER1", "DIS3L2", "EGFR", "EPCAM", "ERCC2", "ERCC3", "ERCC4", "ERCC5", "EXT1", "EXT2",
"EZH2", "FANCA", "FANCB", "FANCC", "FANCD2", "FANCE", "FANCF", "FANCG", "FANCI", "FANCL", "FANCM", "FH", "FLCN", "GATA2", "GPC3", "HNF1A",
"HRAS", "KIT", "MAX", "MEN1", "MET", "MLH1", "MSH2", "MSH6", "MUTYH", "NBN", "NF1", "NF2", "NSD1", "PALB2", "PHOX2B", "PMS1", "PMS2", "PRF1",
"PRKAR1A", "PTCH1", "PTEN", "RAD51C", "RAD51D", "RB1", "RECQL4", "RET", "RHBDF2", "RUNX1", "SBDS", "SDHAF2", "SDHB", "SDHC", "SDHD", "SLX4",
"SMAD4", "SMARCB1", "STK11", "SUFU", "TMEM127", "TP53", "TSC1", "TSC2", "VHL", "WRN", "WT1", "XPA", "XPC", "FGD1", "SDHA", "POLD1", "CDKN1B")


## Filtering by oncotarget genes (dataset contains all missense, splice site, trunc, stop gain and frameshift variants)

library(dplyr)

Tier123MissenseAndTrunc <- filter(Tiering123_MultTumour, Tiering123_MultTumour$Genomic.Feature.Hgnc %in% Oncotarget)


## Removing Superfluous Columns

Subdata <- Tier123MissenseAndTrunc[c(1,9:13,17,18,20)]
```

```
## Ordering based Upon Patient ID#

SubdataOrder <- Subdata[order(Subdata$Participant.Id),]


## Removing rows where both ID# and Gene are the same

SubTestOrder <- SubdataOrder[duplicated(SubdataOrder$Participant.Id, SubdataOrder$Genomic.Feature.Hgnc),]


## Creating vector to remove unique paraticipant IDs (ie removing non minas cases)

SubtestUnique <- SubTestOrder[c(1)]

SubU <- SubtestUnique[duplicated(SubtestUnique$Participant.Id),]



## Removing non-MINAS from Dataframe

Final <- filter(SubTestOrder, SubTestOrder$Participant.Id %in% SubU)


## Seeing how many MINAS cases there are

FinalNumber <- Final[!duplicated(Final$Participant.Id),]



### Getting Ages onto dataset ##################################################
```

```
MPTages <- read.delim("~/Documents/AnthonyProject/Test/MPTages.tsv", na.strings= c("-999", "999"))

## Remove Superfluous Columns

MPTage <- MPTages[c(1,4)]

## Add ages to final table

FinalAge <- merge(MPTage, Final, by="Participant.Id")

## Creating vector to remove unique paraticipant IDs (ie removing non minas cases)

SubtestUnique2 <- FinalAge[c(1)]

SubU2 <- SubtestUnique2[duplicated(SubtestUnique2$Participant.Id),]

## Removing non-MINAS from Dataframe

FinalAge2 <- filter(FinalAge, FinalAge$Participant.Id %in% SubU2)  #subest order 2 was here, that has gene tier as well as partid

## Seeing how many MINAS cases there are

FinalNumber2 <- FinalAge2[!duplicated(FinalAge2$Participant.Id),]

## Remove NA Values to get in final number

MINASageFinal <- na.omit(FinalNumber2)
```

```
### Data for MINAS ages

summary(MINASageFinal$Age.Of.Onset)

MINASagesdata <- MINASageFinal$Age.Of.Onset

MINASagesID <- as.factor(MINASageFinal$Participant.Id)

Ages <- filter(MPTage, !(MPTage$Participant.Id %in% MINASagesID))

AgesFinal <- na.omit(Ages)

## Add ages to final table (Note that some values are lost here)

MPTpheno <- merge(FinalAge, MPTphenotype, by="Participant.Id")


##Adding Phenotypes to dataset##############################################


## Remove Superfluous Columns

MPTphenoClean <- MPTpheno[c(1:10,14)]


## MINAS phenotypes only

MINASpheno <- merge(FinalNumber, MPTphenotype, by="Participant.Id")

MinasPhenoNumber <- MINASpheno[!duplicated(MINASpheno$Participant.Id),]
```

```
## Non-Minas Phenotypes

Phenotype <- filter(MPTphenotype, (MPTphenotype$Participant.Id %in% Tier123MissenseAndTrunc$Participant.Id))

Phenotype2 <- filter(Phenotype, !(Phenotype$Participant.Id %in% MinasPhenoNumber$Participant.Id))

Phenotype2 <- filter(Phenotype, (Phenotype$Participant.Id %in% Subdata$Participant.Id))

NonMinasPhenotypeNumber <- Phenotype2[!duplicated(Phenotype2$Participant.Id),]


## Getting Phenotype datasets wrt MINAS or non-MINAS

NonMinasPhenotypeData <- merge(MPTphenotype, NonMinasPhenotypeNumber, by="Participant.Id" )

MinasPhenotypeData <- merge(MPTphenotype, MinasPhenoNumber, by="Participant.Id" )




## Non-MINAS dataset for phenotype and age analysis

NonMINASmerge <- merge(MPTage, NonMinasPhenotypeData, by="Participant.Id" )

NonMINASmerge$Participant.Id <- factor(NonMINASmerge$Participant.Id)

NonMinasPhenoAge <- NonMINASmerge[c(1,2,6,14,27)]


##Minas dataset for phenotype and age analysis

MINASmerge <- merge(MPTage, MinasPhenotypeData, by="Participant.Id" )

MINASmerge$Participant.Id <- factor(MINASmerge$Participant.Id)
```

```
MinasPhenoAge <- MINASmerge[c(1,2,6,14,27)]

### Finish
```

Supplementary Table 1: List of 94 cancer predisposition genes (CSGs) used for literature search.

**AIP**	**ALK**	**APC**	**ATM**	**BAP1**	**BLM**	**BMPR1A**	**BRCA1**	**BRCA2**	**BRIP1**
**BUB1B**	**CDC73**	**CDH1**	**CDK4**	**CDKN1C**	**CDKN2A**	**CEBPA**	**CEP57**	**CHEK2**	**CYLD**
**DDB2**	**DICER1**	**DIS3L2**	**EGFR**	**EPCAM**	**ERCC2**	**ERCC3**	**ERCC4**	**ERCC5**	**EXT1**
**EXT2**	**EZH2**	**FANCA**	**FANCB**	**FANCC**	**FANCD2**	**FANCE**	**FANCF**	**FANCG**	**FANCI**
**FANCL**	**FANCM**	**FH**	**FLCN**	**GATA2**	**GPC3**	**HNF1A**	**HRAS**	**KIT**	**MAX**
**MEN1**	**MET**	**MLH1**	**MLH2**	**MSH6**	**MUTYH**	**NBN**	**NF1**	**NF2**	**NSD1**
**PALB2**	**PHOX2B**	**PMS1**	**PMS2**	**PRKAR1A**	**PTCH1**	**PTEN**	**RAD51C**	**RAD51D**	**RB1**
**RECQL4**	**RET**	**RHBDF2**	**SBDS**	**SDHAF2**	**SDHB**	**SDHC**	**SDHD**	**SLX4**	**SMAD4**
**SMARCB1**	**STK11**	**SUFU**	**TMEM127**	**TP53**	**TSC1**	**TSC2**	**VHL**	**WRN**	**WT1**
**XPA**	**XPC**	RUNX1	CHK1	CHK2	XPD	MMAC1	RAD5*	TEP-1	BRCA-1
BRCA-2	BRCA-2	WT-1	TP-53	PTCH2					

'*' indicates that all terms matching the text string preceding the * will be found in the search

Supplementary Table 2: Publications discovered containing MINAS Cases in Literature Review

Reference	PMID	Included in historical cohort (Whitworth et al 2016)	Number of Cases where both Variants met Pathogenicity Criteria	Number of Cases where either Variant did not meet Pathogenicity Criteria
Agiannitopoulos et al., 2020	32853479	No	1	0
Andrés et al., 2019	31292799	No	1	0
Ataei-Kachouei et al., 2015	26576347	No	0	1
Augustyn et al. 2011	From Whitworth 2016	Yes	2	0
Balta et al., 2019	30339652	No	5	0
Belanger et al., 2015	25884701	No	1	0
Bell et al. 2002	12019146	Yes	2	0
Bell et al., 2014	12019146	No	1	0
Borg et al. 2000	From Whitworth 2016	Yes	1	0
Brown et al., 2020	32190804	No	1	0
Caldes 2002	From Whitworth 2016	Yes	8	0
Campos et al.	From Whitworth 2016	Yes	1	0

2013				
Carbajal-Mamani et al., 2020	32206661	No	1	0
Choi et al. 2006	16684319	Yes	2	0
Claus et al., 2005	15728167	No	1	0
Cote et al., 2012	21947752	No	1	0
Del Valle et al., 2020	32235514	No	1	2
Ercolino et al. 2014	24361808	Yes	1	0
Foppiani et al. 2008	From Whitworth 2016	Yes	1	0
Fostira et al., 2019	31300551	No	9	0
Frank-Raue et al., 2005	15870131	No	3	0
Friedman et al. 1998	From Whitworth 2016	Yes	4	0
Genomics England	N/A	Yes	6	0

Work Environment				
Ghataorhe et al. 2007	From Whitworth 2016	Yes	3	0
Gong et al., 2012	24371622	No	1	0
Heidemann et al. 2012	22535016	Yes	8	0
Kamory et al., 2006	17189986	No	0	1
Kashiwada et al. 2012	From Whitworth 2016	Yes	1	0
Kast et al. 2012	23164213	Yes	1	0
Kilmartin et al.	From Whitworth 2016	Yes	1	0

1996				
Koren-Michowitz et al., 2005	15726604	No	3	0
Lavie et al., 2011	20924075	No	26	0
Le Duc et al., 2020	33319852	No	1	0
Le Page et al., 2018	29587661	No	2	0
Leegte et al. 2005<br>Frank et al. 2002	From Whitworth 2016	Yes	8	0
Leegte et al.<br>2005	From Whitworth 2016	Yes	4	0
Liede et al. 1998	From Whitworth 2016	Yes	1	0
Liede et al., 1998; Le Page 2020	31753525; 9585617	No	1	0
Lindor et al. 2012	22886683	Yes	1	0
Loader and Rowley, 1998	10464601	No	3	0
Lorca et al., 2019	31285513	No	0	1
Loubser et al.	22486351	Yes	2	0

2012				
Manoukian et al. 2007	From Whitworth 2016	Yes	1	0
Mastroianno et al. 2011	From Whitworth 2016	Yes	4	0
Meynard et al., 2018	28184945	No	1	0
Momozawa et al., 2018	30287823	No	3	0
Monnerat et al. 2007	From Whitworth 2016	Yes	1	0
Moslehi et al. 2000	10733239	Yes	1	0

Musolino et al. 2005	15868448	Yes	1	0
Na et al., 2017	27989354	No	1	0
Nakahara et al., 1997	9419403	No	3	0
Ng et al., 2016	26757417	No	1	0
Njoroge et al., 2017	28702897	No	1	0
Noh et al. 2011	21847643	Yes	3	0
Nomizu et al., 2015	23242612	No	2	0
Nurmi et al., 2019	30927251	No	23	0
Occhi et al., 2010	20530095	No	0	1
Ohmoto et al., 2018	29667044	No	0	1
Ouyang et al., 2019	DOI:10.1200/JCO.2019.37.15_suppl.e13027	No	9	0
Palmirotta et al., 2018	29346284	No	0	1
Papi et al., 2009	19837273	No	1	0
Pazderová et al., 2020	32683879	No	1	0
Pearlman et al., 2017	27978560	No	5	0
Pedroni et al. 2013	From Whitworth 2016	Yes	1	0
Penkert et al., 2018	30086788	No	2	1
Pern et al. 2012	From Whitworth 2016	No	1	0
Pilato et al. 2010	20730485	Yes	1	0

Plon et al. 2008	From Whitworth 2016	Yes	1	0
Puijenbroek et al. 2007	17039270	Yes	1	0
Ramus et al. 1997	From Whitworth 2016	Yes	1	0
Ramus et al., 1997; Le Page 2020	31753525; 8988162	No	3	0
Randall et al. 1998	From Whitworth 2016	Yes	1	0
Randall et al., 1998; Le Page 2020	31753525; 9790802	No	2	0
Rebbeck et al., 2016	27836010	No	90	0
Sarkadi et al., 2019	31263477	No	1	0
Scheenstra et al.	From Whitworth 2016	Yes	1	0

2003				
Sekido et al., 2017	28314314	No	1	0
Silva-Smith and Sussman, 2018	28600700	No	1	0
Smith et al. 2008	From Whitworth 2016	Yes	2	0
Sokolenko et al., 2014	24800916	No	15	0
Soravia et al. 2005	From Whitworth 2016	Yes	1	0
Spannuth et al., 2007	17403394	No	1	0
Stajkovska et al., 2019	31749828	No	1	0
Steffensen et al., 2010	20455026	No	4	0
Stolavora et al., 2020	33050356	No	9	2
Stradella et al., 2019	30580288	No	12	0
Sukumar et al., 2021	33507482	No	3	0
Suspitsin et al., 2013	24415413	No	5	0
Taeubner et al., 2017	29230040	No	0	1
Tedaldi et al., 2017	28423363	No	5	0
Tesoriero et al. 1999	From Whitworth 2016	Yes	1	0
Thiffault et al. 2004	From Whitworth 2016	Yes	1	0

Uhrhammer and Bignon. 2008	18629513	Yes	1	0
Vahteristo et al., 2001	11479205	No	1	0
Valle et al. 2004	From Whitworth 2016	Yes	1	0
Vietri et al., 2013	23940062	No	3	0
Vietri et al., 2020	32388397	No	1	0
Wen et al., 2019	31774011	No	1	0
Whitworth et al. 2015	From Whitworth 2016	Yes	7	0
Yamamoto et al., 2006	16533526	No	1	0
Yilmaz et al., 2020	31845022	No	1	0
Zbuk et al. 2007	From Whitworth 2016	Yes	1	0
Zheng et al., 2020	33224963	No	1	0
Zuradelli et al. 2010	33224963	Yes	4	0

Supplementary Table 3: Details of MINAS cases (n=296) not included in Whitworth et al (2016). Further details for these cases and those in Whitworth et al (2016) in supplementary table 4.

Abbreviations: BC= breast cancer, BBC = bilateral breast cancer, OC = ovarian cancer, PC = prostate cancer, IDC = invasive ductal carcinoma, DCIS = ductal carcinoma in-situ, EnC = Endometrial Cancer, LBC = lobular breast cancer, ALL = acute lymphoblastic leukaemia, CRC = colorectal cancer, FAP = familial adenomatous polyposis, TRIPLE HET = Triple heterozygote, TripNeg = Triple Negative (ER-, PR-, HER2-)

Case	Reference	Sex	Gene 1	Gene 1 variant	Consequence	Gene 2	Gene 2 variant	Consequence	Clinical Features/Phenotype	Multiple Primaries?
1	Le Page et al., 2018	F	BRCA1	c.4327 C>T	Nonsense	BRCA2	c.3170del	Frameshift	OC 40y<br><br>IHC = ER-, PR-, HER2?	N
2	Le Page et al., 2018	F	BRCA1	c.4327 C>T	Nonsense	BRCA2	c.2816insA	Frameshift	BC 52y, OC 63y<br><br>IHC = ER+, PR-, HER2?	Y
3	Belanger et al., 2015	F	BRCA1	c.3770_3771del	Frameshift	BRCA2	c.5946delT	Frameshift	OC 41y	N
4	Rebbeck et al., 2016	F	BRCA1	c.68_69delAG	Frameshift	BRCA2	c.5946delT	Frameshift	BC	N
5	Rebbeck et al., 2016	F	BRCA1	c.68_69delAG	Frameshift	BRCA2	c.5946delT	Frameshift	BC	N
6	Rebbeck et al., 2016	F	BRCA1	c.68_69delAG	Frameshift	BRCA2	c.5946delT	Frameshift	BC	N

7	Rebbeck et al., 2016	F	BRCA 1	c.68_69delAG	Frameshift	BRCA 2	c.5946delT	Frameshift	BC	N
8	Rebbeck et al., 2016	F	BRCA 1	c.68_69delAG	Frameshift	BRCA 2	c.5946delT	Frameshift	BC	N
9	Rebbeck et al., 2016	F	BRCA 1	c.68_69delAG	Frameshift	BRCA 2	c.5946delT	Frameshift	BC	N
10	Rebbeck et al., 2016	F	BRCA 1	c.68_69delAG	Frameshift	BRCA 2	c.5946delT	Frameshift	BC	N
11	Rebbeck et al., 2016	F	BRCA 1	c.68_69delAG	Frameshift	BRCA 2	c.5946delT	Frameshift	BC	N
12	Rebbeck et al., 2016	F	BRCA 1	c.68_69delAG	Frameshift	BRCA 2	c.5946delT	Frameshift	BC	N
13	Rebbeck et al., 2016	F	BRCA 1	c.68_69delAG	Frameshift	BRCA 2	c.5946delT	Frameshift	BC	N
14	Rebbeck et al., 2016	F	BRCA 1	c.68_69delAG	Frameshift	BRCA 2	c.5946delT	Frameshift	BC	N
15	Rebbeck et al., 2016	F	BRCA 1	c.68_69delAG	Frameshift	BRCA 2	c.5946delT	Frameshift	BC	N
16	Rebbeck et al., 2016	F	BRCA 1	c.68_69delAG	Frameshift	BRCA 2	c.5946delT	Frameshift	BC	N
17	Rebbeck et al., 2016	F	BRCA 1	c.68_69delAG	Frameshift	BRCA 2	c.5946delT	Frameshift	OC	N
18	Rebbeck et al., 2016	F	BRCA 1	c.68_69delAG	Frameshift	BRCA 2	c.5946delT	Frameshift	BC + OC	Y
19	Rebbeck et al., 2016	F	BRCA 1	c.68_69delAG	Frameshift	BRCA 2	c.5946delT	Frameshift	BC + OC	Y
20	Rebbeck et al., 2016	F	BRCA 1	c.68_69delAG	Frameshift	BRCA 2	c.5946delT	Frameshift	BC + OC	Y
21	Rebbeck et al., 2016	F	BRCA 1	c.68_69delAG	Frameshift	BRCA 2	c.5946delT	Frameshift	No cancer	N
22	Rebbeck et al., 2016	F	BRCA 1	c.68_69delAG	Frameshift	BRCA 2	c.5946delT	Frameshift	No cancer	N

23	Rebbeck et al., 2016	F	BRCA 1	c.68_69delAG	Frameshift	BRCA 2	c.5946delT	Frameshift	No cancer	N
24	Rebbeck et al., 2016	F	BRCA 1	c.68_69delAG	Frameshift	BRCA 2	c.5946delT	Frameshift	No cancer	N
25	Rebbeck et al., 2016	F	BRCA 1	c.68_69delAG	Frameshift	BRCA 2	c.5946delT	Frameshift	No cancer	N
26	Rebbeck et al., 2016	F	BRCA 1	c.68_69delAG	Frameshift	BRCA 2	c.5946delT	Frameshift	No cancer	N
27	Rebbeck et al., 2016	F	BRCA 1	c.68_69delAG	Frameshift	BRCA 2	c.5946delT	Frameshift	No cancer	N
28	Rebbeck et al., 2016	F	BRCA 1	c.68_69delAG	Frameshift	BRCA 2	c.5946delT	Frameshift	No cancer	N
29	Rebbeck et al., 2016	F	BRCA 1	c.68_69delAG	Frameshift	BRCA 2	c.5946delT	Frameshift	No cancer	N
30	Rebbeck et al., 2016	F	BRCA 1	c.68_69delAG	Frameshift	BRCA 2	c.5946delT	Frameshift	No cancer	N
31	Rebbeck et al., 2016	F	BRCA 1	c.68_69delAG	Frameshift	BRCA 2	c.5946delT	Frameshift	No cancer	N
32	Rebbeck et al., 2016	F	BRCA 1	c.68_69delAG	Frameshift	BRCA 2	c.5946delT	Frameshift	No cancer	N
33	Rebbeck et al., 2016	F	BRCA 1	c.68_69delAG	Frameshift	BRCA 2	c.5946delT	Frameshift	No cancer	N
34	Rebbeck et al., 2016	F	BRCA 1	c.68_69delAG	Frameshift	BRCA 2	c.5946delT	Frameshift	No cancer	N
35	Rebbeck et al., 2016	F	BRCA 1	c.68_69delAG	Frameshift	BRCA 2	c.5722_5723delCT	Frameshift	BC	N
36	Rebbeck et al., 2016	F	BRCA 1	c.1016delA (p.Lys339Argfs)	Frameshift	BRCA 2	c.7379_7382delACAA	Frameshift	BC	N
37	Rebbeck et al., 2016	F	BRCA 1	c.1504_1508del5 (p.Leu502fs)	Frameshift	BRCA 2	c.2798_2799delCA (p.Thr933fs)	Frameshift	BC	N

38	Rebbeck et al., 2016	F	BRCA 1	c.1504_1508del5 (p.Leu502fs)	Frameshift	BRCA 2	c.462_463delAA (p.Asp156Terfs)	Frameshift	BC + OC	Y
39	Rebbeck et al., 2016	F	BRCA 1	c.1687C > T (p.Gln563Ter)	Nonsense	BRCA 2	c.6469C > T (Q2157*)	Nonsense	BC + OC	Y
40	Rebbeck et al., 2016	F	BRCA 1	c.181 T > G (p.Cys61Gly)	Missense	BRCA 2	c.1318_1319dupCT (p.Thr441fs)	Frameshift	BC	N
41	Rebbeck et al., 2016	F	BRCA 1	c.181 T > G (p.Cys61Gly)	Missense	BRCA 2	c.1318_1319dupCT (p.Thr441fs)	Frameshift	No cancer	N
42	Rebbeck et al., 2016	F	BRCA 1	c.181 T > G (p.Cys61Gly)	Missense	BRCA 2	c.1318_1319dupCT (p.Thr441fs)	Frameshift	No cancer	N
43	Rebbeck et al., 2016	F	BRCA 1	c.211A > G (p.Arg71Gly)	Missense	BRCA 2	c.4380_4381delTT (p.Ser1461fs)	Frameshift	BC	N
44	Rebbeck et al., 2016	F	BRCA 1	c.213-12A > G (IVS5-12A>G)	Novel Splice Site (Leading to FS)	BRCA 2	c.7180A > T (p.Arg2394Ter)	Nonsense	BC	N
45	Rebbeck et al., 2016	F	BRCA 1	c.2389G > T (p.Glu797Ter)	Nonsense	BRCA 2	c.3068dupA (p.Asn1023Lysfs)	Frameshift	BC	N
46	Rebbeck et al., 2016	F	BRCA 1	c.2405_2406delTG	Frameshift	BRCA 2	c.4284dupT (p.Gln1429fs)	Frameshift	BC + OC	Y
47	Rebbeck et al., 2016	F	BRCA 1	c.246delT (p.Val83fs)	Frameshift	BRCA 2	c.517-2A > G (IVS6-2A>G)	Splice Acceptor	BC	N

48	Rebbeck et al., 2016	F	BRCA 1	c.246delT (p.Val83fs)	Frameshift	BRCA 2	c.517-2A > G (IVS6-2A>G)	Splice Acceptor	No cancer	N
49	Rebbeck et al., 2016	F	BRCA 1	c.301 + 1G > A	Splice Donor	BRCA 2	c.5682C > G (p.Tyr1894Ter)	Nonsense	BC	N
50	Rebbeck et al., 2016	F	BRCA 1	c.3048_3052dup5 (p.Asn1018Metfs)	Frameshift	BRCA 2	c.2830A > T (p.Lys944Ter)	Nonsense	BC	N
51	Rebbeck et al., 2016	F	BRCA 1	c.3048_3052dup5 (p.Asn1018Metfs)	Frameshift	BRCA 2	c.2830A > T (p.Lys944Ter)	Nonsense	No cancer	N
52	Rebbeck et al., 2016	F	BRCA 1	c.3228_3229delAG (p.Gly1077fs)	Frameshift	BRCA 2	c.3689delC (p.Ser1230fs)	Frameshift	No cancer	N
53	Rebbeck et al., 2016	F	BRCA 1	c.3228_3229delAG (p.Gly1077fs)	Frameshift	BRCA 2	c.9253dupA (p.Thr3085Asnfs)	Frameshift	BC	N
54	Rebbeck et al., 2016	F	BRCA 1	c.3400G > T (p.Glu1134Ter)	Nonsense	BRCA 2	c.2808_2811delACAA (p.Ala938Profs)	Frameshift	BC	N
55	Rebbeck et al., 2016	F	BRCA 1	c.3400G > T (p.Glu1134Ter)	Nonsense	BRCA 2	c.2808_2811delACAA (p.Ala938Profs)	Frameshift	No cancer	N
56	Rebbeck et al., 2016	F	BRCA 1	c.3477_3480 delAAAG (p.Ile1159fs)	Frameshift	BRCA 2	c.9401delG (p.Gly3134fs)	Frameshift	BC	N
57	Rebbeck et al., 2016	F	BRCA 1	c.3627dupA	Frameshift	BRCA 2	c.6724_6725delGA	Frameshift	BC	N

58	Rebbeck et al., 2016	F	BRCA 1	c.3700_3704del5 (p.Val1234fs)	Frameshift	BRCA 2	c.1815dupA (p.Pro606Thrfs)	Frameshift	BC	N
59	Rebbeck et al., 2016	F	BRCA 1	c.3756_3759delGT CT	Frameshift	BRCA 2	c.7757G > A (p.Trp2586Ter)	Nonsense	BC	N
60	Rebbeck et al., 2016	F	BRCA 1	c.3759_3760delTA (p.Lys1254fs)	Frameshift	BRCA 2	c.9699_9702 delTATG (p.Cys3233fs)	Frameshift	BC + OC	Y
61	Rebbeck et al., 2016	F	BRCA 1	c.3770_3771delAG	Frameshift	BRCA 2	c.5946delT	Frameshift	BC	N
62	Rebbeck et al., 2016	F	BRCA 1	c.3770_3771delAG	Frameshift	BRCA 2	c.5946delT	Frameshift	BC + OC	Y
63	Rebbeck et al., 2016	F	BRCA 1	c.390C > A (p.Tyr130Ter)	Nonsense	BRCA 2	c.3018delA (p.Gly1007fs)	Frameshift	BC	N
64	Rebbeck et al., 2016	F	BRCA 1	3910delG (E1257fs)	Frameshift	BRCA 2	c.2830A > T (p.Lys944Ter)	Nonsense	BC	N
65	Rebbeck et al., 2016	F	BRCA 1	c.4035delA (p.Glu1346fs)	Frameshift	BRCA 2	c.658_659delGT (p.Val220fs)	Frameshift	No cancer	N
66	Rebbeck et al., 2016	F	BRCA 1	c.4065_4068delTC AA (p.Asn1355fs)	Frameshift	BRCA 2	c.5350_5351delAA (p.Asn1784fs)	Frameshift	BC	N
67	Rebbeck et al., 2016	F	BRCA 1	c.4186?4357+?dup	Unknown	BRCA 2	c.2636_2637delCT (p.Asp878_Ser879T er)	Nonsense	BC	N

68	Rebbeck et al., 2016	F	BRCA 1	c.4186?4357+?dup	Unknown	BRCA 2	c.2636_2637delCT (p.Asp878_Ser879Ter)	Nonsense	No cancer	N
69	Rebbeck et al., 2016	F	BRCA 1	c.427G > T (p.Glu143Ter)	Nonsense	BRCA 2	c.8730delT (p.Asn2910fs)	Frameshift	BC	N
70	Rebbeck et al., 2016	F	BRCA 1	c.5030_5033 delCTAA (p.Thr1677fs)	Frameshift	BRCA 2	c.1399A > T (p.Lys467Ter)	Nonsense	BC	N
71	Rebbeck et al., 2016	F	BRCA 1	c.5123C > A (p.Ala1708Glu)	Missense	BRCA 2	c.6275_6276delTT (p.Leu2092fs)	Frameshift	BC	N
72	Rebbeck et al., 2016	F	BRCA 1	c.5136G > A (p.Trp1712Ter)	Nonsense	BRCA 2	c.4965delC (p.Cys1654_Tyr1655insTer)	Nonsense	BC	N
73	Rebbeck et al., 2016	F	BRCA 1	c.5193 + 1delG (IVS18+1delG)	Splice Donor	BRCA 2	c.658_659delGT (p.Val220fs)	Frameshift	BC	N
74	Rebbeck et al., 2016	F	BRCA 1	c.5266dupC (p.Gln1756Profs)	Frameshift	BRCA 2	c.8364G > A (p.Trp2788Ter)	Nonsense	BC	N
75	Rebbeck et al., 2016	F	BRCA 1	c.5266dupC (p.Gln1756Profs)	Frameshift	BRCA 2	c.5946delT	Frameshift	BC	N
76	Rebbeck et al., 2016	F	BRCA 1	c.5266dupC (p.Gln1756Profs)	Frameshift	BRCA 2	c.5946delT	Frameshift	BC	N
77	Rebbeck et al., 2016	F	BRCA 1	c.5266dupC (p.Gln1756Profs)	Frameshift	BRCA 2	c.5946delT	Frameshift	BC	N

78	Rebbeck et al., 2016	F	BRCA 1	c.5266dupC (p.Gln1756Profs)	Frameshift	BRCA 2	c.5946delT	Frameshift	BC + OC	Y
79	Rebbeck et al., 2016	F	BRCA 1	c.5266dupC (p.Gln1756Profs)	Frameshift	BRCA 2	c.5946delT	Frameshift	No cancer	N
80	Rebbeck et al., 2016	F	BRCA 1	c.5266dupC (p.Gln1756Profs)	Frameshift	BRCA 2	c.4478_4481delAAAG (p.Glu1493fs)	Frameshift	BC	N
81	Rebbeck et al., 2016	F	BRCA 1	c.5266dupC (p.Gln1756Profs)	Frameshift	BRCA 2	c.5645C > A (p.Ser1882Ter)	Nonsense	BC + OC	Y
82	Rebbeck et al., 2016	F	BRCA 1	c.962G > A (p.Trp321Ter)	Nonsense	BRCA 2	c.2231C > G (p.Ser744Ter)	Nonsense	BC	N
83	Rebbeck et al., 2016	F	BRCA 1	c.212 + 1G > A	Splice Donor	BRCA 2	c.739_740delAT*	Frameshift	BC	N
84	Rebbeck et al., 2016	F	BRCA 1	c.3196G > T (p.Glu1066Ter)	Nonsense	BRCA 2	c.658_659delGT (p.Val220fs)	Frameshift	BC	N
85	Rebbeck et al., 2016	F	BRCA 1	c.3916_3917delTT (p.Leu1306fs)	Frameshift	BRCA 2	c.5380delG* (p.Asn1793_Val1794insTer)	Frameshift	BC + OC	Y
86	Rebbeck et al., 2016	F	BRCA 1	c.548-?_4185 + ?del	Large Deletion	BRCA 2	c.2269A > T	Nonsense	BC + OC	Y
87	Na et al., 2017	M	BRCA 1	c.5266dup; (p.Q1756Pfs*74)	Frameshift	BRCA 2	c.5946del; (p.S1982Rfs*22)	Frameshift	PC 57y (localised) Biopsy Gleason Score: 6	N

88	Spannuth et al., 2007	F	BRCA 1	5382insC (p.Gln1756Profs)	Frameshift	BRCA 2	6174delT (p.Ser1982fs)	Frameshift	IDC 46y bilateral OC 57y	Y
89	Meynard et al., 2018	F	BRCA 1	c.1016dupA (p.V340Gfs*6)	Frameshift	BRCA 2	c.6814delA (p.R2272Efs*8)	Frameshift	BBC 46y (distinct histologies right/left) Right IDC; IHC = ER+, PR-, HER2 WT. Left IDC; IHC = TripNeg	Y
90	Nomizu et al., 2015	F	BRCA 1	c.188T>A (p.Leu63Ter)	Frameshift	BRCA 2	c.5576_5579del (p.Ile1859fs)	Frameshift	BC 55y IHC = ER+ PR- HER2-, BRCA1- BRCA2-	N
91	Vietri et al., 2013	F	BRCA 1	c.547+2T>A; (p.Gln148Aspfsx51)	Frameshift	BRCA 2	c.2830A>T (p.Lys944X)	Nonsense	TRIPLE HET: BRCA2 c.426-57A>G BBC 32y; IHC = ER+, PR+, HER2-	Y
92	Vietri et al., 2013	F	BRCA 1	c.547+2T>A; (p.Gln148Aspfsx51)	Frameshift	BRCA 2	c.2830A>T (p.Lys944X)	Nonsense	TRIPLE HET: BRCA2 c.426-57A>G BC 39y	N
93	Vietri et al., 2013	M	BRCA 1	c.547+2T>A; (p.Gln148Aspfsx51)	Frameshift	BRCA 2	c.2830A>T (p.Lys944X)	Nonsense	TRIPLE HET: BRCA2 c.426-57A>G No symptoms 71y	N
94	Lavie et al., 2011	F	BRCA 1	185delAG (p.Glu23fs)	Frameshift	BRCA 2	6174delT (p.Ser1982fs)	Frameshift	BC	N

95	Lavie et al., 2011	F	BRCA 1	185delAG (p.Glu23fs)	Frameshift	BRCA 2	6174delT (p.Ser1982fs)	Frameshift	BC	N
96	Lavie et al., 2011	F	BRCA 1	185delAG (p.Glu23fs)	Frameshift	BRCA 2	6174delT (p.Ser1982fs)	Frameshift	BC	N
97	Lavie et al., 2011	F	BRCA 1	185delAG (p.Glu23fs)	Frameshift	BRCA 2	6174delT (p.Ser1982fs)	Frameshift	BC	N
98	Lavie et al., 2011	F	BRCA 1	185delAG (p.Glu23fs)	Frameshift	BRCA 2	6174delT (p.Ser1982fs)	Frameshift	BC	N
99	Lavie et al., 2011	F	BRCA 1	185delAG (p.Glu23fs)	Frameshift	BRCA 2	6174delT (p.Ser1982fs)	Frameshift	BC	N
10 0	Lavie et al., 2011	F	BRCA 1	185delAG (p.Glu23fs)	Frameshift	BRCA 2	6174delT (p.Ser1982fs)	Frameshift	BC	N
10 1	Lavie et al., 2011	F	BRCA 1	185delAG (p.Glu23fs)	Frameshift	BRCA 2	6174delT (p.Ser1982fs)	Frameshift	BC	N
10 2	Lavie et al., 2011	F	BRCA 1	185delAG (p.Glu23fs)	Frameshift	BRCA 2	6174delT (p.Ser1982fs)	Frameshift	BC	N
10 3	Lavie et al., 2011	F	BRCA 1	185delAG (p.Glu23fs)	Frameshift	BRCA 2	6174delT (p.Ser1982fs)	Frameshift	BC	N
10 4	Lavie et al., 2011	F	BRCA 1	185delAG (p.Glu23fs)	Frameshift	BRCA 2	6174delT (p.Ser1982fs)	Frameshift	OC?	N
10 5	Lavie et al., 2011	F	BRCA 1	185delAG (p.Glu23fs)	Frameshift	BRCA 2	6174delT (p.Ser1982fs)	Frameshift	No Cancer?	N

106	Lavie et al., 2011	F	BRCA 1	185delAG (p.Glu23fs)	Frameshift	BRCA 2	6174delT (p.Ser1982fs)	Frameshift	No Cancer?	N
107	Lavie et al., 2011	F	BRCA 1	185delAG (p.Glu23fs)	Frameshift	BRCA 2	6174delT (p.Ser1982fs)	Frameshift	No Cancer?	N
108	Lavie et al., 2011	F	BRCA 1	185delAG (p.Glu23fs)	Frameshift	BRCA 2	6174delT (p.Ser1982fs)	Frameshift	No Cancer?	N
109	Lavie et al., 2011	F	BRCA 1	185delAG (p.Glu23fs)	Frameshift	BRCA 2	6174delT (p.Ser1982fs)	Frameshift	No Cancer?	N
110	Lavie et al., 2011	F	BRCA 1	185delAG (p.Glu23fs)	Frameshift	BRCA 2	6174delT (p.Ser1982fs)	Frameshift	No Cancer?	N
111	Lavie et al., 2011	F	BRCA 1	185delAG (p.Glu23fs)	Frameshift	BRCA 2	6174delT (p.Ser1982fs)	Frameshift	No Cancer?	N
112	Lavie et al., 2011	M	BRCA 1	185delAG (p.Glu23fs)	Frameshift	BRCA 2	6174delT (p.Ser1982fs)	Frameshift	No Cancer?	N
113	Lavie et al., 2011	M	BRCA 1	185delAG (p.Glu23fs)	Frameshift	BRCA 2	6174delT (p.Ser1982fs)	Frameshift	No Cancer?	N
114	Lavie et al., 2011	M	BRCA 1	185delAG (p.Glu23fs)	Frameshift	BRCA 2	6174delT (p.Ser1982fs)	Frameshift	No Cancer?	N
115	Lavie et al., 2011	M	BRCA 1	185delAG (p.Glu23fs)	Frameshift	BRCA 2	6174delT (p.Ser1982fs)	Frameshift	No Cancer?	N
116	Lavie et al., 2011	F	BRCA 1	5382insC (p.Gln1756Profs)	Frameshift	BRCA 2	6174delT (p.Ser1982fs)	Frameshift	BC	N

117	Lavie et al., 2011	F	BRCA1	5382insC (p.Gln1756Profs)	Frameshift	BRCA2	6174delT (p.Ser1982fs)	Frameshift	BC	N
118	Lavie et al., 2011	M	BRCA1	5382insC (p.Gln1756Profs)	Frameshift	BRCA2	6174delT (p.Ser1982fs)	Frameshift	No Cancer y?	N
119	Lavie et al., 2011	M	BRCA1	5382insC (p.Gln1756Profs)	Frameshift	BRCA2	6174delT (p.Ser1982fs)	Frameshift	No Cancer y?	N
120	Koren-Michowitz et al., 2005	F	BRCA1	(p.Glu23fs) or (p.Gln1756Profs)	Frameshift	BRCA2	(p.Ser1982fs)	Frameshift	TRIPLE HET: Fanconi anaemia - *FANCC c.456+4A>T 2* pathogenic 33y no cancer history	N
121	Koren-Michowitz et al., 2005	F	BRCA1	(p.Glu23fs) or (p.Gln1756Profs)	Frameshift	BRCA2	(p.Ser1982fs)	Frameshift	TRIPLE HET: BLOOM SYNDROME - BLM c.2207_2212delinsTAGATTC (p.Tyr736fs) 2* pathogenic BC 45y	N
122	Koren-Michowitz et al., 2005	F	BRCA1	(p.Glu23fs) or (p.Gln1756Profs)	Frameshift	BRCA2	(p.Ser1982fs)	Frameshift	BC	N

123	Loader and Rowley, 1998	M	BRCA 1	E1694X	Nonsense	BRCA 2	6633del5	Frameshift	"in his 30's" with no known cancer	N
124	Loader and Rowley, 1998	M	BRCA 1	E1694X	Nonsense	BRCA 2	6633del5	Frameshift	"in his 30's" with no known cancer	N
125	Loader and Rowley, 1998	F	BRCA 1	E1694X	Nonsense	BRCA 2	6633del5	Frameshift	"in her 30's" with no known cancer	N
126	Steffensen et al., 2010	F	BRCA 1	c.5096G > A (p.Arg1699Gln)	Missense	BRCA 2	c.631 + 4A>G	Splice site Variant	BC 53y OC 59y	Y
127	Steffensen et al., 2010	F	BRCA 1	c.5096G > A (p.Arg1699Gln)	Missense	BRCA 2	c.631 + 4A>G	Splice site Variant	No known cancer y?; <59 (daughter of #104)	N
128	Steffensen et al., 2010	M	BRCA 1	c.5096G > A (p.Arg1699Gln)	Missense	BRCA 2	c.631 + 4A>G	Splice site Variant	BC 76y	N
129	Steffensen et al., 2010	M	BRCA 1	c.5096G > A (p.Arg1699Gln)	Missense	BRCA 2	c.631 + 4A>G	Splice site Variant	No known cancer y?; <59 (Son of #104)	N
130	Claus et al., 2005	F	BRCA 1	W321X	Nonsense	BRCA 2	3398del5	Frameshift	BC 37y	N

131	Nomizu et al., 2015	F	BRCA1	L63X	Nonsense	BRCA2	5804del4	Frameshift	BC 41y<br><br>EnC 46y	Y
132	Tedaldi et al., 2017	F	BRCA1	c.5266dupC<br><br>(p.Gln1756fs)	Frameshift	BRCA2	c.2049_2050del<br><br>(p.Ile684fs)	Frameshift	IDC 53y	N
133	Nurmi et al., 2019	F	BRCA1	c.3485delA	Frameshift	ATM	c.6908dupA	Frameshift	TRIPLE HET; RAD51C: c.93delG2* Pathogenic, Frameshift<br>OC 60y (high-grade serous)	N
134	Nurmi et al., 2019	F	BRCA1	c.3485delA	Frameshift	ATM	c.6908dupA	Frameshift	OC (Endometrioid) 53y	N
135	Nurmi et al., 2019	F	BRCA1	c.3485delA	Frameshift	FANCM	c.5101C>T	Nonsense	OC 42y (clear cell)	N
136	Nurmi et al., 2019	F	BRCA1	c.4097-2A>G	Splice Acceptor	FANCM	c.5101C>T	Nonsense	IDC 60.1y; IHC = ER+ PR+ HER2?	N
137	Nurmi et al., 2019	F	BRCA1	c.4327C>T	Nonsense	CHEK2	c.1100delC	Frameshift	IDC 49.0y, BBC 55.8y; IHC = ER+ PR? HER2?	Y
138	Nurmi et al., 2019	F	BRCA1	c.4327C>T	Nonsense	FANCM	c.5101C>T	Nonsense	IDC 37.2y; IHC = Trip Neg<br><br>OC 40.7y	Y
139	Nurmi et al., 2019	F	BRCA1	c.5266dupC	Frameshift	FANCM	c.5101C>T	Nonsense	IDC 48.8y; IHC = Trip Neg	N

140	Nurmi et al., 2019	F	BRCA2	c.771_775delTCAAA	Frameshift	ATM	c.7570G>C	Missense	IDC 76.6y; IHC = ER+ PR- HER2?	N
141	Nurmi et al., 2019	F	BRCA2	c.7480C>T	Nonsense	PALB2	c.1592delT	Frameshift	DCIS + BBC 38.5y + 52.4y; IHC = ER+ PR+ HER2?	Y
142	Nurmi et al., 2019	F	BRCA2	c.7480C>T	Nonsense	CHEK2	c.1100delC	Frameshift	DCIS 61.7y; IHC = ER- PR- HER2+	N
143	Nurmi et al., 2019	F	BRCA2	c.8327T>G	Nonsense	FANCM	c.5101C>T	Nonsense	IDC 36.4y; IHC = ER+ PR+ HER2-	N
144	Nurmi et al., 2019	F	BRCA2	c.8327T>G	Nonsense	FANCM	c.5101C>T	Nonsense	LBC, BBC 53.2y + 77.3y; IHC = ER? PR+ HER2?<br><br>OC 82y	Y
145	Nurmi et al., 2019	F	BRCA2	c.9118-2A>G	Splice Acceptor	FANCM	c.5101C>T	Nonsense	IDC, BBC 53.5y + 59.6y; IHC = ER+ PR+ HER2?	Y
146	Stradella et al., 2019	F	BRCA1	c.3607C>T p.(Arg1203*)	Nonsense	TP53	c.659A>G p.(Tyr220Cys)	Unknown	OC 45y<br><br>Phenotype = HBOC	N
147	Sokolenko et al., 2014	F	BRCA1	5382insC	Frameshift	BLM	Q548X	Nonsense	OC 50y<br><br>BC 68y IHC = Trip neg	Y
148	Sokolenko et al., 2014	F	BRCA1	5382insC	Frameshift	BLM	Q548X	Nonsense	Waldenstrom disease 58y Δ<br><br>BC 67y; IHC = Trip neg	Y

149	Sokolenko et al., 2014	F	BRCA1	C61G	Missense	ATM	E1978X	Missense	BC 44y; IHC = ER+, PR? HER2?	N
150	Sokolenko et al., 2014	F	BRCA1	C61G	Missense	ATM	E1978X	Missense	BC 40y ; IHC = ER-, PR? HER2?	N
151	Sokolenko et al., 2014	F	BRCA1	5382insC	Frameshift	BLM	Q548X	Nonsense	BC 42y; IHC = ER-, PR? HER2?	N
152	Sokolenko et al., 2014	F	BRCA1	C61G	Missense	CHEK2	c.1100delC	Frameshift	BC 45y ; IHC = ER-, PR? HER2?	N
153	Sokolenko et al., 2014	F	BRCA1	5382insC	Frameshift	CHEK2	c.444+1G>A<br><br>(IVS2+1G >A)	Splice Donor	BC 58y; IHC = ER+, PR? HER2?	N
154	Sokolenko et al., 2014	F	CHEK2	1100delC	Frameshift	BLM	Q548X	Nonsense	BC = 54y; IHC = Trip Neg	N
155	Andrés et al., 2019	F	BRCA1	c.5123C>A (p.Ala1708Glu)	Missense	ATM	c.2413C>T<br><br>(p.Arg805X)	Nonsense	BC 55y; IHC = Trip Neg<br><br>Jaundice + elevated transaminases;<br><br>Pancreas apudoma adenocarcinoma 62y<br><br>Endometrial Cancer 71y	N
156	Ouyang et al., 2019	F	BRCA1	c.5095C>T (p.Arg1699Trp)	Missense	PALB2	c.1059del<br><br>p.K353Nfs*3	Frameshift/<br><br>Nonsense	BC 42y; IHC = Trip Neg	N

157	Cote et al., 2012	F	BRCA2	c.9004G>A (E3002K)	Missense	PALB2	c.2323C>T (Q775X)	Nonsense	BC 45y<br>Family History: Excess of colorectal cancers	N
158	Tedaldi et al., 2017	F	BRCA2	c.6998dupT (p.Pro2334fs)	Frameshift	FANCA	c.987_990del (p.Thr329fs)	Frameshift	IDC 30y	N
159	Tedaldi et al., 2017	F	BRCA2	c.658_659delGT (p.Val220fs)	Frameshift	BRIP1	c.2992_2995del (p.Lys998fs)	Frameshift	IDC x2 37y	Y
160	Tedaldi et al., 2017	F	BRCA2	c.8487+1G>A	Splice Donor	ATM	c.3275C>A (p.Ser1092Ter)	Nonsense	DCIS 38y<br>IDC 45y	Y
161	Momozawa et al., 2018	F	BRCA2	p.Ala938fs	Frameshift	ATM	c.72+1G>A	Splice Donor	IDC 45y; IHC = ER+, PR+, HER2-	N
162	Momozawa et al., 2018	F	BRCA2	p.Arg3128*	Frameshift	CHEK2	c.573+1delG	Splice Donor	IDC 56y; IHC = ER+, PR-, HER2?	N
163	Sarkadi et al., 2019	F	BRCA1	p.Ile90Serfs	Frameshift	RET	p.Cys634Trp	Missense	Medullary Thyroid Carcinoma 16y	N
164	Fostira et al., 2019	F	BRCA1	c.5497G>A (p.Val1833Met)	Missense	PALB2	c.2257C>T (p.Arg753Ter)	Nonsense	BC 44y	N
165	Fostira et al., 2019	F	BRCA2	c.2490_2491insT (p.Val831Cysfs)	Frameshift	RAD51C	c.904+5G>T	Unknown	BC 46y<br>BC 56y	Y
166	Fostira et al., 2019	F	BRCA2	c.7879A>T (p.Ile2627Phe)	Missense	SDHC	c.397C>T (p.Arg133Ter)	Nonsense	BBC 31y	Y

167	Nurmi et al., 2019	F	BRCA2	c.7480C>T	Nonsense	CHEK2	c.1100delC	Frameshift	TRIP HET; FANCM: c.5101C>T LP2*, nonsense IDC 55.4y; IHC = ER+ PR+ HER2-	N
168	Sokolenko et al., 2014	F	BRCA1	5382insC	Frameshift	CHEK2	del5395 (c.909-?_1095+?del)	Frameshift	BC 52y<br><br>IHC = ER+, PR+, HER2-	N
169	Nurmi et al., 2019	F	PALB2	c.1592delT	Frameshift	FANCM	c.5101C>T	Nonsense	IDC 33.4y; IHC = ER+ PR+ HER2+	N
170	Nurmi et al., 2019	F	PALB2	c.1592delT	Frameshift	FANCM	c.5101C>T	Nonsense	IDC 55y; IHC = Trip Neg	N
171	Nurmi et al., 2019	F	CHEK2	c.1100delC	Frameshift	ATM	c.6908dupA	Frameshift	IDC 53.2y; IHC = ER+ PR- HER2- Bladder cancer 58y	Y
172	Nurmi et al., 2019	F	CHEK2	c.1100delC	Frameshift	ATM	c.7570G>C	Missense	IDC 60y; IHC = ER+ PR+ HER2-	N
173	Nurmi et al., 2019	F	CHEK2	c.1100delC	Frameshift	FANCM	c.5101C>T	Nonsense	LBC 46y; IHC = ER+ PR+ HER2 -	N
174	Nurmi et al., 2019	F	CHEK2	c.1100delC	Frameshift	FANCM	c.5101C>T	Nonsense	IDC 87.5y; IHC = ER+ PR+ HER2-	N
175	Nurmi et al., 2019	F	ATM	c.6908dupA	Frameshift	FANCM	c.5101C>T	Nonsense	OC 46y (mucinous)	N

176	Stradella et al., 2019	F	MLH1	c.244A>G; p.(Thr82Ala)	Missense	MEN1	c.784-9G>A	Spice Site	Hyperplasia 29y, Pituitary adenoma 36y<br><br>Parathyroid tumour 39y, Neuroendocrine tumour 41y<br><br>Hepatic haemangiomas<br><br>Phenotype = MEN1	N
177	Stradella et al., 2019	F	MLH1	c.244A>G; p.(Thr82Ala)	Missense	MEN1	c.784-9G>A	Spice Site	Uterine carcinoma 41y, Colorectal cancer 48y<br><br>Haemangiomas 45y, Hyperparathyroidism 45y<br><br>Hepatic haemangiomas<br><br>Phenotype = MEN1, HNPCC	Y

178	Stradella et al., 2019	F	TSC2	c.5227C>T; p.(Arg1743Trp)	Missense	RAD51D	c.694C>T p.(Arg232*)	Nonsense	Subcutaneous benign tumours 6y<br><br>Epilepsy<br><br>Phenotype = Tuberous sclerosis	N
179	Stradella et al., 2019	F	PALB2	c.3256 C>T; p.(Arg1086*)	Nonsense	ATM	c.3802delG<br><br>p.(Val1268*)	Nonsense	BC 54y<br><br>Pancreatic cancer 59y<br><br>Phenotype = HBOC	Y
180	Stradella et al., 2019	F	SDHB	c.505C>T<br><br>p.(Gln169*)	Nonsense	FANCA	c.3558dupG<br><br>p.(Arg1187Glufs*28)	Frameshift/<br><br>Nonsense	OC 49y<br><br>Phenotype = HBOC	N
181	Njoroge et al., 2017	F	CDH1	c.2287G>T	Nonsense	PMS2	c.2445+1G>T	Splice Donor	LBC 51y<br>Follicular adenoma(thyroid) + micropapillary carcinoma 52y<br>Phenotype = HDGC + Lynch syndrome	Y
182	Sokolenko et al., 2014	F	NBN	657del5	Frameshift	BLM	Q548X	Nonsense	BC 51y; IHC = ER+, PR+, HER2-	N
183	Sokolenko et al., 2014	F	CHEK2	c.444+1G>A	Splice Donor	NBN	657del5	Frameshift	BC 48y; IHC = ER-, PR+, HER2+	N
184	Vahteristo et al., 2001	F	MSH6	c.2983G>T (p.Glu995Ter)	Nonsense	CHEK2	c.1100del (p.Thr367fs)	Frameshift	BC 34y<br><br>CRC 34y	Y

185	Brown et al., 2020	M	MEN1	c0.525_526insTT (p.Ala176Leufs*10)	Frameshift/ Nonsense	RET	c.1889G>A (p.Cys630Tyr)	Missense	Medullary Thyroid Carcinoma 27y; IHC = calcitonin +	N
186	Sokolenko et al., 2014	F	CHEK2	c.444+1G>A	Splice Donor	NBN	657del5	Frameshift	BC 53y	N
187	Sokolenko et al., 2014	F	CHEK2	c.444+1G>A	Splice Donor	NBN	657del5	Frameshift	BBC 59/60y; IHC = ER+, PR+, HER2+	Y
188	Sokolenko et al., 2014	F	CHEK2	c.444+1G>A	Splice Donor	NBN	657del5	Frameshift	BC 53y; IHC = ER+, PR-, HER2?	N
189	Pearlman et al., 2017	F	MSH2	c.2388delT (p.V797Lfs*15)	Frameshift	MUTYH	c.1187G>A (p.G396D)	Missense	30y CRC; MMR deficient Extensive family history of cancers: Father colon 60; Pat. halfsister 1 endometrial 54; Pat. half-sister 2 thyroid 39; Pat. aunt melanoma 60s; Mat. aunt breast 47; Mat. grandmother brain 52	N
190	Yamamoto et al., 2006	M	PTPN11	c.182A>G (p.Asp61Gly)	Missense	FLT3/ITD	Data not avaliable	Data not avaliable	ALL 5y survival > 154 months	N

191	Pearlman et al., 2017	F	ATM	c.7271T>G (p.V2424G)	Missense	CHEK 2	c.1100del	Frameshift	CRC 44y; MMR proficient tumour Family History: Father prostate 74; Mother breast 50s; Mat. grandmother breast 50s	N
192	Rebbeck et al., 2016	F	BRCA 1	c.3155delA (3274delA)	Frameshift	BRCA 2	c.3160_ 3163delGATA	Frameshift	BC	N
193	Rebbeck et al., 2016	F	BRCA 1	c.3155delA (3274delA)	Frameshift	BRCA 2	c.3160_ 3163delGATA	Frameshift	No cancer	N
194	Rebbeck et al., 2016	F	BRCA 1	c.3700_3704del5 (p.Val1234fs)	Frameshift	BRCA 2	c.681 + 1G > A	Splice Site Acceptor	BC	N
195	Rebbeck et al., 2016	F	BRCA 1	c.3839_3843 delinsAGGC	Frameshift	BRCA 2	c.1636delT (p.Cys546fs)	Frameshift	OC	N
196	Rebbeck et al., 2016	F	BRCA 1	c.3839_3843 delinsAGGC	Frameshift	BRCA 2	c.1636delT (p.Cys546fs)	Frameshift	No cancer	N
197	Rebbeck et al., 2016	F	BRCA 1	c.5251C > T (p.Arg1751Ter)	Nonsense	BRCA 2	c.6753_6754delTT	Frameshift	BC	N
198	Rebbeck et al., 2016	F	BRCA 1	c.5406 + 664_*827 3del	Unknown	BRCA 2	c.9748dupT (p.Ser3250fs)	Frameshift	BC	N
199	Ouyang et al., 2019	F	BRCA 1	p.T1677Ifs*2	Frameshift/ Nonsense	BRCA 2	c.631+1G>A	Splice Site	BC 35y; IHC = Trip Neg	N

200	Ouyang et al., 2019	F	BRCA1	p.V1120Dfs*11	Frameshift/ Nonsense	BRCA2	p.D687*fs*1	Frameshift/ Nonsense	Unknown Primary, y?	N
201	Fostira et al., 2019	F	BRCA1	c.5406 + 664_*8273del	Unknown	BRCA2	c.9748dupT (p.Ser3250fs)	Frameshift	BC 31y; IHC = Trip Neg BC 49y; IHC = ?	Y
202	Ng et al., 2016	F	BRCA2	c.5164_5465delAG	Frameshift	BARD1	c.1487C>G (S496X)	Nonsense	IDC 35y; IHC = ER+ PR+ HER2-	N
203	Nurmi et al., 2019	F	BRCA2	c.7480C>T	Nonsense	FANCM	c.5791C>T	Nonsense	IDC 46.2y; IHC = Trip Neg	N
204	Stradella et al., 2019	F	BRCA1	c.1961delA p.(Lys654Serfs*47)	Frameshift/ Nonsense	APC	c.423-3T>A	Splice Site	Colorectal polyposis 76y Phenotype = AFAP	N
205	Stradella et al., 2019	F	BRCA1	c.2309C>A p.(Ser770*)	Nonsense	XPA	c.553C>T p.(Gln185*)	Nonsense	OC 51y Phenotype = HBOC	N
206	Stradella et al., 2019	F	BRCA1	c.2309C>A p.(Ser770*)	Nonsense	XPA	c.553C>T p.(Gln185*)	Nonsense	OC 37y Phenotype = HBOC	N
207	Sokolenko et al., 2014	F	BRCA1	c.3247del5	Frameshift	CHEK2	del5395 (c.909-?_1095+?del)	Frameshift	BC = 42y	N
208	Bell et al., 2014	F	BRCA1	c.81-?_134 + ? del (p.Cys27*)	Nonsense	TP53	c.375 + 2T > C	Splice Site	20y metaplastic carcinoma + IDC (BBC) IHC = Both Trip Neg	Y

209	Ouyang et al., 2019	F	BRCA1	p.K463*	Nonsense	PALB2	c.751C>T (p.Gln251Ter)	Nonsense	BC 35y; IHC = Trip Neg	N
210	Ouyang et al., 2019	M	BRCA2	p.A2825Vfs*15	Frameshift/ Nonsense	PALB2	p.Q158Rfs*19	Frameshift/ Nonsense	PC 48y	N
211	Ouyang et al., 2019	F	BRCA1	p.V757Ffs*8	Frameshift/ Nonsense	ATM	c.-30-1G>A	Splice Site	BC 47y; IHC = Trip Neg	N
212	Ouyang et al., 2019	F	BRCA1	p.H399Tfs*2	Frameshift/ Nonsense	MSH6	p.R495*	Nonsense	OC 43y	N
213	Ouyang et al., 2019	F	BRCA2	p.A1193Lfs*4	Frameshift/ Nonsense	PMS2	p.Q714*	Nonsense	BC 64y; IHC = Trip Neg	N
214	Papi et al., 2009	F	BRCA1	c.3228_3229delAG (p.Gly1077fs)	Frameshift	MEN1	c.908delGCT	In-Frame Deletion	2x insulinoma 33y Mediastinal lipoma 35y hyperparathyroidism 32y, Hyperprolactinemia 33y	Y
215	Sekido et al., 2017	F	BRCA2	Clinical Diagnosis	Missense	KIT	p.Trp557Leu	Missense	TRIP HET; KIT: p.Lys558Glu BC + GIST 65y; IHC = Trip Neg Family History = Uncle BC y?, mother BC 57y, sister OC 53y	Y
216	Pearlman et al., 2017	F	BRCA2	c.1755_1759del, (p.K585Nfs*3)	Frameshift/ Nonsense	CHEK2	c.751A>T (p.I251F)	Missense	CRC 47y; MMR proficient Site = rectum	N

217	Tedaldi et al., 2017	F	BRCA1	c.5266dupC (p.Gln1756fs)	Frameshift	ERCC3	c.1757delA (p.Gln586fs)	Frameshift	IDC 39y	N
218	Fostira et al., 2019	F	BRCA2	c.1813dupA<br><br>(p.Ile605Asnfs)	Frameshift	RECQL4	c.1879-1G>A	Splice Site	BC 46y<br><br>OC 63	Y
219	Fostira et al., 2019	F	BRCA2	c.(80+1_81-1)_(593+1_594-1)del	Unknown	ATM	c.3576G>A<br><br>(p.Lys1192=)	Synonomous<br><br>(Splice site)	BC 40y	N
220	Nurmi et al., 2019	F	ATM	c.7570G>C	Missense	FANCM	c.5791C>T	Nonsense	IDC, BBC 28y + 30.2y; IHC = ER+ PR+ HER2-	Y
221	Balta et al., 2019	M	FANCA	c.1374delC	Frameshift	ATM	c.8977C>T	Missense	Additional variant FANCA: c.1361-1370delCCTCCTTTGG<br>In-Frame Deletion<br>No known cancer 36y	N
222	Balta et al., 2019	F	FANCA	c.1374delC	Frameshift	ATM	c.8977C>T	Missense	Additional variant FANCA: c.1361-1370delCCTCCTTTGG<br>In-Frame Deletion<br>No known cancer 36y	N

223	Balta et al., 2019	M	FANCA	c.1374delC	Frameshift	ATM	c.8977C>T	Missense	Homozygous variant FANCA (CHILD OF 188 + 187)<br>FA phenotype: microcephaly, growth retardation, cafe-au-lait spots, hyperpigmentation, bilateral microphthalmia, microcornea, severe malnutrition in the absence of any physical congenital skeletal abnormalities<br><br>presented at 9y with pancytopenia and macrocytosis<br><br>severe pancytopenia and fever 13y, died with no signs of leukaemia	N

224	Balta et al., 2019	F	FANCA	c.1374delC	Frameshift	ATM	c.8977C>T	Missense	Homozygous variant FANCA (CHILD OF 188 + 187)<br>FA phenotype: microcephaly, growth retardation, cafe-au-lait spots, hyperpigmentation, bilateral microphthalmia, microcornea, severe malnutrition in the absence of any physical congenital skeletal abnormalities<br><br>mild pancytopenia and macrocytosis 8y, no sign of leukaemia<br><br>Died 16y, cause unknown	N

225	Balta et al., 2019	F	FANCA	c.1374delC	Frameshift	ATM	c.8977C>T	Missense	Homozygous variant ATM (CHILD OF 188 + 187)<br>12y presented with mentally retardation and severe malnutrition and clinical signs of Ataxia–telangiectasia: (ataxia, telangiectasias of the skin and conjunctivae, frequent sinopulmonary infections, bilateral nystagmus, strabismus, hypotonia, muscle weakness, and decreased serum immunoglobulin A level)<br><br>No sign and symptom of secondary cancer including leukaemia and lymphoma as well as no clinical and laboratory data suggestive of FA	N

									were present<br><br>Died 13y due to severe sinopulmonary infections	

226	Pearlman et al., 2017	M	APC	c.2377C>T<br>(p.Q793X)	Nonsense	PMS2	c.1281delT<br>(p.H428Tfs*20)	Frameshift/<br>Nonsense	29y Polyposis;<br>MMR proficient tumour<br>Extensive family history of cancers:<br>Fathera(APC) colon 20s, FAP;<br>Mat. aunt cervical 24, multiple myeloma 50;<br>Mat. grandmother colon and lymphoma 66;<br>Mat. grandfather prostate 70s	N
227	Stradella et al., 2019	F	FH	c.905-2A>G<br>p.?	Splice Site	BARD1	c.157delT;<br>p.(Cys53Valfs*5)	Frameshift/<br>Nonsense	Cutaneous leiomyomas 40y<br>Colorectal polyposis 52y<br>Phenotype = Reed's syndrome	Y
228	Stradella et al., 2019	F	ATM	c.3712_3716del<br>p.(Leu1238Lysfs*6)	Frameshift/<br>Nonsense	FANCA	c.2602-1G>C<br>p.?	Splice Site	BC 35y<br>Phenotype = HBOC	N
229	Gong et al., 2012	F	MLH1	c.677G>T<br>R226L	Missense	MSH6	3959del4	Frameshift/<br>Nonsense	TRIPLE HET, also BRCA2 mutant<br>Endometrial hyperplasia with atypia, 41y<br>IHC = Loss of MLH1 and PMS2	N

230	Silva-Smith and Sussman, 2018	M	PMS2	c.1882C > T	Nonsense	BMPR1A	c.25A > T	Nonsense	Bladder cancer + CRC 39y; IHC=loss of PMS2 staining<br><br>Relatives as young as 5 with polyps	Y
231	Ouyang et al., 2019	F	PMS2	c.1A>G<br><br>(p.Met1Val)	Missense	ATM	p.E503*	Nonsense	CRC 67y	N
232	Carbajal-Mamani et al., 2020	F	ATM	Deletion between exons 62 and 63	Deletion<br><br>Nonsense/Frameshift?	PALB2	c.2840T>C (p.Leu947Ser)	Missense	46y benign fibroids<br><br>DCIS 49y; IHC = ER+, PR+<br><br>OC 50y (clear cell)<br><br>Family history of breast (x2) and colon (x1) cancer	Y
233	Fostira et al., 2019	F	ERCC3	c.576_583delCGTGATCC<br><br>(p.Pro192fs)	Frameshift	FANCC	c.346-1G>A	Splice Site Acceptor	TripNeg BC 34y	N
234	Fostira et al., 2019	F	PALB2	c.2747_2748+4delAGGTAA	Frameshift	SLX4	c.4089_4090delAG	Frameshift	BC 44y<br><br>OC 48y	Y
235	Fostira et al., 2019	F	PALB2	c.2257C>T<br><br>(p.Arg753Ter)	Nonsense	ATM	c.1562_1563delAG	Frameshift	BC 33y	N
236	Stajkovska et al., 2019	M	MLH1	c.333_334delTC p.His112CysfsTer9	Frameshift/<br><br>Nonsense	TP53	c.847C > T; p.Arg283Cys	Missense	Glioblastoma 13y	N

237	Genomics England<br><br>Work Environment	F	BRCA2	13:32340629:CTT>C	Frameshift	CHEK2	22:28725040:T>A	Stop Gain	infiltrating duct and lobular carcinoma, 25-30y	N
238	Genomics England<br><br>Work Environment	F	BRCA2	13:32340629:CTT>C	Frameshift	CHEK2	22:28725040:T>A	Stop Gain	breast adenocarcinoma, 45-50y	N
239	Genomics England<br><br>Work Environment	F	ATM	11:108299862:GA>G	Frameshift	BLM	15:90763016:C>T	Stop Gain	tubular breast adenocarcinoma, 45-50y	N
240	Genomics England<br><br>Work Environment	F	ATM	11:108299862:GA>G	Frameshift	BLM	15:90763016:C>T	Stop Gain	breast carcinoma, 45-50y	N
241	Genomics England<br><br>Work Environment	M	BRCA2	13:32339228:GAA>G	Frameshift	POLD1	19:50415720:G>C	Unknown	PGL, 40-45y	N
242	Genomics England	M	XPA	9:97684982:TC>T	Frameshift	ERCC5	13:102875551:TGAAAA>T	Frameshift	pituitary adenoma, 40-45y	N

	Work Environment									
243	Palmirotta et al., 2018	F	BRCA1	c.1687C>T (p.Gln563Ter)	Nonsense	BRCA2	c.9976A>T (p.Lys3326Ter)	Nonsense	BC 40y + 47y; BBC 54y<br><br>Melanoma 54y	Y
244	Ataei-Kachouei et al., 2015	F	BRCA2	p.Ala938ProfsX21	Nonsense	STK11	p.S422G	Missense	MIDC 38y ; IHC = ER+, PR+, HER2?	N
245	Stradella et al., 2019	F	APC	c.5826_5829del<br><br>p.(Asp1942Glufs*27)	Frameshift/<br><br>Nonsense	EXO1	c.1900C>T<br><br>p.(Arg634*)	Nonsense	Colorectal polyposis 52y<br><br>Colorectal cancer 53y<br><br>Phenotype = AFAP	Y
246	Momozawa et al., 2018	F	BRCA2	p.Gly2529fs	Frameshift	CHEK2	p.Ala523Thr	Missense	BC 44y; IHC = ER +, PR+, HER2?	N
247	Occhi et al., 2010	F	MEN1	E45Q	Missense	AIP	c.871G>A<br><br>(V291M)	Missense	Acromegaly 30y<br><br>No other MEN1 clinical symptoms	N
248	Yilmaz et al., 2020	M	PMS2	c.187G>A<br><br>(p. V63M)	Missense	MSH6	c.3261delC<br><br>p. F1088fs*2	Frameshift/<br><br>Nonsense	CRC 63y<br><br>Lynch Syndrome	N
249	Nakahara et al., 1997	M	MSH2	c.1916A>G (p.His639Arg)	Missense	MLH1	c.397G>T<br><br>(p.Gly133Ter)	Nonsense	CRC + stomach cancer 41y	Y

250	Nakahara et al., 1997	F	MSH2	c.1916A>G (p.His639Arg)	Missense	MLH1	c.397G>T<br><br>(p.Gly133Ter)	Nonsense	Additional MLH1 variant: A>G Ile (219) Val; Exon 8 Likely Benign (Synonomous) multiple CRCs + uterine cancer 57y	Y
251	Nakahara et al., 1997	F	MSH2	c.1916A>G (p.His639Arg)	Missense	MLH1	c.397G>T<br><br>(p.Gly133Ter)	Nonsense	Multiple CRCs + uterine cancer 55y	Y
252	Pearlman et al., 2017	F	MSH2	c.80C>T<br><br>(p.P27L)	Missense	MLH1	c.2252_2253delAA<br><br>(p.K751Sfs*3)	Frameshift/<br><br>Nonsense	CRC 28y<br><br>CRC 46y; MMR deficient	Y
253	Taeubner et al., 2017	?	PTCH 1	p.(Gly38Glu)	Missense	PTCH 2	p.(His622Tyr)	Missense	congenital embryonal rhabdomyosarcoma no tumour loss of heterozygosity (LOH) in PTCH1 and PTCH2.<br>Tumour had increased GLI1 and SMO expression, suggesting increased SHH signalling	N
254	Kamory et al., 2006	M	MLH1	p.Val716Met	Missense	MSH2	c.2210+1G>C	Splice Site<br><br>(Exon 13 deletion out of frame)	multiple synchronous colorectal carcinoma 25y<br>IHC = loss of MLH1 and MSH2	N

									expression in both primaries	
255	Frank-Raue et al., 2005	M	MEN1	IVS5 + 1G>A	Splice Site	RET	Y791F	Missense	Primary hyperparathyroidism, pancreatic tumor, Fibroma; 35y	Y
256	Frank-Raue et al., 2005	M	MEN1	IVS5 + 1G>A	Splice Site	RET	Y791F	Missense	Primary hyperparathyroidism, pituitary tumo, pancreatic tumor, Fibroma; y?; brother of #13	Y
257	Frank-Raue et al., 2005	F	MEN1	IVS5 + 1G>A	Splice Site	RET	Y791F	Missense	No clinical features, y?; daughter of #13	N
258	Lorca et al., 2019	F	POLE	c.3857G > A p.(Arg1286His)	Nonsense	NTHL1	c.527T > C p.(Ile176Thr)	Nonsense	Adenomatous Polyposis x24 70y endometrial hyperplasia 70y hypothyroidism 70y	Y
259	Wen et al., 2019	M	BRCA1	S1841Vfs*2 (c.5521delA)	Frameshift	BRCA2	Q1886* (c.5656C>T)	Nonsense	Gastric cancer 50y	N
260	Stolavora et al., 2020	F	POT1	c.347C>T (p.P116L)	Missense	CHEK2	c.909-2028_1095+330del5395 (p.M304Lfs*15)	Frameshift	Melanoma 41y Melanoma 42y Melanoma 44y BC 47y	Y

261	Stolavora et al., 2020	F	OCA2	c.1211C>T (p.T404M)	Missense	KAT6A	c.1138G>T (p.E380*)	Nonsense	No cancer 29y	N
262	Stolavora et al., 2020	M	TYRP1	c.1054_1057del4 (p.N353Vfs*31)	Frameshift	TRPM1	Δe2-7 (p.?)		Melanoma 36y	N
263	Stolavora et al., 2020	M	SLC45A2	Δe1-2 (p.?)	Frameshift	GSTM3	c.393C>A (p.Y131*)	Nonsense	Melanoma 25y	N
264	Stolavora et al., 2020	F	TYR	c.1037-7T>A (p.?)		FANCC	c.455dupA (p.N152Kfs*9)	Frameshift	BC 52y Melanoma 66y CRC 66y	Y
265	Stolavora et al., 2020	F	NBN	c.1723G>T (p.E575*)	Nonsense	NFKBIE	c.165_169dup5 (p.E57Afs*51)	Frameshift/Nonsense	Melanoma 9y	N
266	Stolavora et al., 2020	M	BRCA2	c.7007G>A (p.R2336H)	Missense	IFIH1	c.2464C>T (p.R822*)	Nonsense	Melanoma 22y	N
267	Stolavora et al., 2020	M	BRCA2	c.8168_8172ins4 (p.Y2726Mfs*10)	Frameshift	TYRP1	c.1254C>A (p.Y418*)	Nonsense	Melanoma 36y Non-Hodgkin Lymphoma 38y	Y
268	Stolavora et al., 2020	F	BRCA1	c.4214delT (p.I1405Kfs*10)	Frameshift	ATM	c.7630-2A>C (p.?)	Splice site	Triple Het - *MUTYH* c.1187G>A (p.G396D) Melanoma 46y OC 46y BC 49y	Y
269	Stolavora et al., 2020	F	ATM	c.381delA (p.V128*)	Nonsense	WRN	c.1105C>T (p.R369*)	Nonsense	Melanoma 41y Melanoma 50y	Y
270	Stolavora et al., 2020	F	RAD51D	c.405+2T>C (p.?)	Splice site	CHEK2	c.917G>C (p.G306A)	Missense	Melanoma 26y	N
271	Zheng et al., 2020	F	BRCA1	c.3348_3351delAGTT p.V1117Rfs*11	Frameshift	MSH2	large deletion of exons 4-16	Frameshift	Endometrial carcinoma 52y	N
272	Pazderová et al., 2020		BRCA2	c.8487G>T p.Gln2829His	Splice Site	CHEK2	Deletion of exons 9-10	Frameshift	Pancreatic Cancer 50y	N

27 3	Sukumar et al., 2021	F	BRCA 1	c.181T > G	Missense	BRCA 2	c.4398_4402delAC ATT	Frameshift	Triple Het - CHEK2 1100delC<br>BilatBC 55y; IHC(1) = ER-,PR+,HER2-, IHC(2) =ER+,PR+,HER2-	Y
27 4	Sukumar et al., 2021	F	BRCA 1	c.181T > G	Missense	CHEK 2	c.1100delC	Frameshift	BC 34y; IHC = ER+,PR-,HER2-	N
27 5	Sukumar et al., 2021	F	BRCA 1	c.181T > G	Missense	CHEK 2	c.1100delC	Frameshift	No cancer 29y	N
27 6	Agiannitop oulos et al., 2020	F	MSH2	Deletion of exons 11-16		PALB2	c.757_758delCT p.(Leu253Ilefs*3)	Frameshift	Endometrial Cancer 42y	N
27 7	Le Duc et al., 2020	M	TP53	c.394A>G	Missense	RAD5 1C	Deletion of exons 5-9		38y Basal Cell Carcinoma<br>41y Sabaceous Gland Carcinoma	Y
27 8	Vietri et al., 2020	F	BRCA 1	c.3756_3759delGT CT (p.Ser1253Argfs)	Frameshift	APC	c.3927_3931delAA AGA (p.Glu1309AspfsX4)	Frameshift	Profuse FAP 18y<br>Desmoid Tumour 22y<br>OC 45y	Y
27 9	Del Valle et al., 2020	F	FANC A	c.2602-1G>C p.?	Splice Site	ATM	Unknown	Unknown	BC 35y	N
28 0	Del Valle et al., 2020	F	FANC A	c.3588dup p.(Arg1187Glufs*2 8)	Frameshift	SDHB	Unknown	Unknown	OC 49y	N
28 1	Del Valle et al., 2020	M	FANC L	c.1111_1114dup p.(Thr372Asnfs*13)	Frameshift	MLH1	Unknown	Unknown	CRC 29y	N
28 2	Ramus et al., 1997; Le Page 2020	F	BRCA 1	c.3916-3917del	Frameshift	BRCA 2	c.5380delG	Frameshift	BC 30y<br>OC 36y	Y

283	Ramus et al., 1997; Le Page 2020	F	BRCA1	c.1687C>T	Missense	BRCA2	c.6469C>T	Missense	BC 46y OC 58y	Y
284	Ramus et al., 1997; Le Page 2020	F	BRCA1	c.2405_2406del	Frameshift	BRCA2	c.4285C>T	Missense	BC 52y OC 52y	Y
285	Randall et al., 1998;Le Page 2020	F	BRCA1	c.68_69delAG	Frameshift	BRCA2	c.5946delT	Frameshift	OC 57y	N
286	Randall et al., 1998;Le Page 2020	F	BRCA1	c.68_69delAG	Frameshift	BRCA2	c.5946delT	Frameshift	OC 50y	N
287	Liede et al., 1998; Le Page 2020	F	BRCA1	c.68_69delAG	Frameshift	BRCA2	c.5946delT	Frameshift	BC 48y OC 50y	Y
288	Suspitsin et al., 2013	F	BRCA1	5382insC	Frameshift	BLM	Q548X	Nonsense	BC 68y IHC = TripNeg	N
289	Suspitsin et al., 2013	F	BRCA1	5382insC	Frameshift	BLM	Q548X	Nonsense	BC 68y IHC = TripNeg	N
290	Suspitsin et al., 2013	F	CHEK2	c.1100delC	Frameshift	BLM	Q548X	Nonsense	BC 51y IHC = ER+,PR+,HER2-	N
291	Suspitsin et al., 2013	F	CHEK2	5395del	Frameshift	BRCA1	5382insC	Frameshift	BC 52y IHC = ER+,PR+,HER2-	N
292	Suspitsin et al., 2013	F	NBN	657del5	Frameshift	BLM	Q548X	Nonsense	BC 48y IHC = ER-,PR+,HER2+	N
293	Penkert et al., 2018	F	ATM	p.(Cys2931*)	Nonsense	CHEK2	exon 9–10 del	Frameshift	BC 39y	N
294	Penkert et al., 2018	F	CDKN2A	p.(Arg98*)	Nonsense	RECQL4	c.1390+1G>C	Splice Site	BC 32y IHC = HER2+	N
295	Penkert et al., 2018	F	FANCI	p.(Arg1285*)	Nonsense	PMS2	exon 3–8 del	Frameshift	BC 30y IHC = HER2+	N

29<br>6	Ohmoto et<br>al., 2018	M	PMS2	c.631 C> T<br>p.Arg211Ter	Nonsense	CHEK<br>2	c.1111 C>T<br>p.His371Tyr	Missense	CRC 58y	N

Supplementary Table 4: Full details of 290 cases with MINAS identified from the literature (and not previously reported in Whitworth et al 2016), and 6 cases from the UK 100,000 Genomes Project data were identified and combined with the 89 cases highlighted in Whitworth et al 2016 to provide a final total of 385 individuals with MINAS (see supplementary Table S1).

Case	Reference	Sex	Gene 1	Gene 1 variant	Consequence	Clinvar	Gene 2	Gene 2 variant	Consequence	Clinvar	Clinical Features/Phenotype	Subgroup	Age At First Cancer Diagnosis (Age at last non-cancer checkup if no cancer)	Age at First Breast Cancer Diagnosis	Exact, Average or Unknown Age of first cancer	Multiple Primaries?
1	Le Page et al., 2018	F	BRCA1	c.4327 C>T	Nonsense	P3*	BRCA2	c.3170del	Frameshift	P3*	Epithelial OC (eOC) 40y	Recent	40	N/A	EXACT	N

											IHC = ER-, PR-, HER2?					
2	Le Page et al., 2018	F	BR CA 1	c.4327 C>T	Nonsense	P3*	BRC A2	c.2816insA	Frameshi ft	P3*	BC 52y, eOC 63y<br><br>IHC = ER+, PR-, HER2?	Recen t	52	52	EXA CT	Y
3	Belang er et al., 2015	F	BR CA 1	c.3770_377 1del	Frameshif t	P3*	BRC A2	c.5946delT	Frameshi ft	P3*	eOC 41y	Recen t	41	N/A	EXA CT	N
4	Rebbe ck et al., 2016	F	BR CA 1	c.68_69del AG	Frameshif t	P3*	BRC A2	c.5946delT	Frameshi ft	P3*	BC = 39.9y (23–67)	Recen t	39.9	39.9	AVE RAG E	N
5	Rebbe ck et al., 2016	F	BR CA 1	c.68_69del AG	Frameshif t	P3*	BRC A2	c.5946delT	Frameshi ft	P3*	BC = 39.9y (23–67)	Recen t	39.9	39.9	AVE RAG E	N
6	Rebbe ck et al., 2016	F	BR CA 1	c.68_69del AG	Frameshif t	P3*	BRC A2	c.5946delT	Frameshi ft	P3*	BC = 39.9y (23–67)	Recen t	39.9	39.9	AVE RAG E	N
7	Rebbe ck et al., 2016	F	BR CA 1	c.68_69del AG	Frameshif t	P3*	BRC A2	c.5946delT	Frameshi ft	P3*	BC = 39.9y (23–67)	Recen t	39.9	39.9	AVE RAG E	N
8	Rebbe ck et al., 2016	F	BR CA 1	c.68_69del AG	Frameshif t	P3*	BRC A2	c.5946delT	Frameshi ft	P3*	BC = 39.9y (23–67)	Recen t	39.9	39.9	AVE RAG E	N

9	Rebbeck et al., 2016	F	BRCA1	c.68_69delAG	Frameshift	P3*	BRCA2	c.5946delT	Frameshift	P3*	BC = 39.9y (23–67)	Recent	39.9	39.9	AVERAGE	N
10	Rebbeck et al., 2016	F	BRCA1	c.68_69delAG	Frameshift	P3*	BRCA2	c.5946delT	Frameshift	P3*	BC = 39.9y (23–67)	Recent	39.9	39.9	AVERAGE	N
11	Rebbeck et al., 2016	F	BRCA1	c.68_69delAG	Frameshift	P3*	BRCA2	c.5946delT	Frameshift	P3*	BC = 39.9y (23–67)	Recent	39.9	39.9	AVERAGE	N
12	Rebbeck et al., 2016	F	BRCA1	c.68_69delAG	Frameshift	P3*	BRCA2	c.5946delT	Frameshift	P3*	BC = 39.9y (23–67)	Recent	39.9	39.9	AVERAGE	N
13	Rebbeck et al., 2016	F	BRCA1	c.68_69delAG	Frameshift	P3*	BRCA2	c.5946delT	Frameshift	P3*	BC = 39.9y (23–67)	Recent	39.9	39.9	AVERAGE	N
14	Rebbeck et al., 2016	F	BRCA1	c.68_69delAG	Frameshift	P3*	BRCA2	c.5946delT	Frameshift	P3*	BC = 39.9y (23–67)	Recent	39.9	39.9	AVERAGE	N
15	Rebbeck et al., 2016	F	BRCA1	c.68_69delAG	Frameshift	P3*	BRCA2	c.5946delT	Frameshift	P3*	BC = 39.9y (23–67)	Recent	39.9	39.9	AVERAGE	N
16	Rebbeck et al., 2016	F	BRCA1	c.68_69delAG	Frameshift	P3*	BRCA2	c.5946delT	Frameshift	P3*	BC = 39.9y (23–67)	Recent	39.9	39.9	AVERAGE	N

17	Rebbeck et al., 2016	F	BRCA1	c.68_69delAG	Frameshift	P3*	BRCA2	c.5946delT	Frameshift	P3*	OC = 59.2y (57–62)	Recent	59.2	N/A	AVERAGE	N
18	Rebbeck et al., 2016	F	BRCA1	c.68_69delAG	Frameshift	P3*	BRCA2	c.5946delT	Frameshift	P3*	BC + OC = 41.9y (26–53)	Recent	41.9	41.9	AVERAGE	Y
19	Rebbeck et al., 2016	F	BRCA1	c.68_69delAG	Frameshift	P3*	BRCA2	c.5946delT	Frameshift	P3*	BC + OC = 41.9y (26–53)	Recent	41.9	41.9	AVERAGE	Y
20	Rebbeck et al., 2016	F	BRCA1	c.68_69delAG	Frameshift	P3*	BRCA2	c.5946delT	Frameshift	P3*	BC + OC = 41.9y (26–53)	Recent	41.9	41.9	AVERAGE	Y
21	Rebbeck et al., 2016	F	BRCA1	c.68_69delAG	Frameshift	P3*	BRCA2	c.5946delT	Frameshift	P3*	No cancer = 39.1y (20–68)	Recent	39.1	N/A	AVERAGE	N
22	Rebbeck et al., 2016	F	BRCA1	c.68_69delAG	Frameshift	P3*	BRCA2	c.5946delT	Frameshift	P3*	No cancer = 39.1y (20–68)	Recent	39.1	N/A	AVERAGE	N
23	Rebbeck et al., 2016	F	BRCA1	c.68_69delAG	Frameshift	P3*	BRCA2	c.5946delT	Frameshift	P3*	No cancer = 39.1y (20–68)	Recent	39.1	N/A	AVERAGE	N
24	Rebbeck et al., 2016	F	BRCA1	c.68_69delAG	Frameshift	P3*	BRCA2	c.5946delT	Frameshift	P3*	No cancer = 39.1y (20–68)	Recent	39.1	N/A	AVERAGE	N

25	Rebbeck et al., 2016	F	BRCA1	c.68_69delAG	Frameshift	P3*	BRCA2	c.5946delT	Frameshift	P3*	No cancer = 39.1y (20–68)	Recent	39.1	N/A	AVERAGE	N
26	Rebbeck et al., 2016	F	BRCA1	c.68_69delAG	Frameshift	P3*	BRCA2	c.5946delT	Frameshift	P3*	No cancer = 39.1y (20–68)	Recent	39.1	N/A	AVERAGE	N
27	Rebbeck et al., 2016	F	BRCA1	c.68_69delAG	Frameshift	P3*	BRCA2	c.5946delT	Frameshift	P3*	No cancer = 39.1y (20–68)	Recent	39.1	N/A	AVERAGE	N
28	Rebbeck et al., 2016	F	BRCA1	c.68_69delAG	Frameshift	P3*	BRCA2	c.5946delT	Frameshift	P3*	No cancer = 39.1y (20–68)	Recent	39.1	N/A	AVERAGE	N
29	Rebbeck et al., 2016	F	BRCA1	c.68_69delAG	Frameshift	P3*	BRCA2	c.5946delT	Frameshift	P3*	No cancer = 39.1y (20–68)	Recent	39.1	N/A	AVERAGE	N
30	Rebbeck et al., 2016	F	BRCA1	c.68_69delAG	Frameshift	P3*	BRCA2	c.5946delT	Frameshift	P3*	No cancer = 39.1y (20–68)	Recent	39.1	N/A	AVERAGE	N
31	Rebbeck et al., 2016	F	BRCA1	c.68_69delAG	Frameshift	P3*	BRCA2	c.5946delT	Frameshift	P3*	No cancer = 39.1y (20–68)	Recent	39.1	N/A	AVERAGE	N
32	Rebbeck et al., 2016	F	BRCA1	c.68_69delAG	Frameshift	P3*	BRCA2	c.5946delT	Frameshift	P3*	No cancer = 39.1y (20–68)	Recent	39.1	N/A	AVERAGE	N

33	Rebbeck et al., 2016	F	BRCA1	c.68_69delAG	Frameshift	P3*	BRCA2	c.5946delT	Frameshift	P3*	No cancer = 39.1y (20–68)	Recent	39.1	N/A	AVERAGE	N
34	Rebbeck et al., 2016	F	BRCA1	c.68_69delAG	Frameshift	P3*	BRCA2	c.5946delT	Frameshift	P3*	No cancer = 39.1y (20–68)	Recent	39.1	N/A	AVERAGE	N
35	Rebbeck et al., 2016	F	BRCA1	c.68_69delAG	Frameshift	P3*	BRCA2	c.5722_5723delCT	Frameshift	P3*	BC = 39.9y (23–67)	Recent	39.9	39.9	AVERAGE	N
36	Rebbeck et al., 2016	F	BRCA1	c.1016delA (p.Lys339Argfs)	Frameshift	P3*	BRCA2	c.7379_7382delACAA	Frameshift	P3*	BC = 39.9y (23–67)	Recent	39.9	39.9	AVERAGE	N
37	Rebbeck et al., 2016	F	BRCA1	c.1504_1508del5 (p.Leu502fs)	Frameshift	P1*	BRCA2	c.2798_2799delCA (p.Thr933fs)	Frameshift	P3*	BC = 39.9y (23–67)	Recent	39.9	39.9	AVERAGE	N
38	Rebbeck et al., 2016	F	BRCA1	c.1504_1508del5 (p.Leu502fs)	Frameshift	P1*	BRCA2	c.462_463delAA (p.Asp156Terfs)	Frameshift	P3*	BC + OC = 41.9y (26–53)	Recent	41.9	41.9	AVERAGE	Y
39	Rebbeck et al., 2016	F	BRCA1	c.1687C > T (p.Gln563Ter)	Nonsense	P3*	BRCA2	c.6469C > T (Q2157*)	Nonsense	P3*	BC + OC = 41.9y (26–53)	Recent	41.9	41.9	AVERAGE	Y
40	Rebbeck et	F	BRCA1	c.181 T > G	Missense	P3*	BRCA2	c.1318_1319dupCT	Frameshift	P3*	BC = 39.9y (23–67)	Recent	39.9	39.9	AVERAGE	N

	al., 2016			(p.Cys61Gly)				(p.Thr441fs)								
41	Rebbeck et al., 2016	F	BRCA1	c.181 T > G (p.Cys61Gly)	Missense	P3*	BRCA2	c.1318_1319dupCT (p.Thr441fs)	Frameshift	P3*	No cancer = 39.1y (20–68)	Recent	39.1	N/A	AVERAGE	N
42	Rebbeck et al., 2016	F	BRCA1	c.181 T > G (p.Cys61Gly)	Missense	P3*	BRCA2	c.1318_1319dupCT (p.Thr441fs)	Frameshift	P3*	No cancer = 39.1y (20–68)	Recent	39.1	N/A	AVERAGE	N
43	Rebbeck et al., 2016	F	BRCA1	c.211A > G (p.Arg71Gly)	Missense	P3*	BRCA2	c.4380_4381delTT (p.Ser1461fs)	Frameshift	P3*	BC = 39.9y (23–67)	Recent	39.9	39.9	AVERAGE	N
44	Rebbeck et al., 2016	F	BRCA1	c.213-12A > G (IVS5-12A>G)	Novel Splice Site (Leading to FS)	P3*	BRCA2	c.7180A > T (p.Arg2394Ter)	Nonsense	P3*	BC = 39.9y (23–67)	Recent	39.9	39.9	AVERAGE	N
45	Rebbeck et al., 2016	F	BRCA1	c.2389G > T (p.Glu797Ter)	Nonsense	P3*	BRCA2	c.3068dupA (p.Asn1023Lysfs)	Frameshift	P3*	BC = 39.9y (23–67)	Recent	39.9	39.9	AVERAGE	N
46	Rebbeck et al., 2016	F	BRCA1	c.2405_2406delTG	Frameshift	P2*	BRCA2	c.4284dupT (p.Gln1429fs)	Frameshift	P3*	BC + OC = 41.9y (26–53)	Recent	41.9	41.9	AVERAGE	Y
47	Rebbeck et al., 2016	F	BRCA1	c.246delT (p.Val83fs)	Frameshift	P3*	BRCA2	c.517-2A > G (IVS6-2A>G)	Splice Acceptor	P3*	BC = 39.9y (23–67)	Recent	39.9	39.9	AVERAGE	N

48	Rebbeck et al., 2016	F	BRCA1	c.246delT (p.Val83fs)	Frameshift	P3*	BRCA2	c.517-2A > G (IVS6-2A>G)	Splice Acceptor	P3*	No cancer = 39.1y (20–68)	Recent	39.1	N/A	AVERAGE	N
49	Rebbeck et al., 2016	F	BRCA1	c.301 + 1G > A	Splice Donor	P/LP2*	BRCA2	c.5682C > G (p.Tyr1894Ter)	Nonsense	P3*	BC = 39.9y (23–67)	Recent	39.9	39.9	AVERAGE	N
50	Rebbeck et al., 2016	F	BRCA1	c.3048_3052dup5 (p.Asn1018Metfs)	Frameshift	P3*	BRCA2	c.2830A > T (p.Lys944Ter)	Nonsense	P3*	BC = 39.9y (23–67)	Recent	39.9	39.9	AVERAGE	N
51	Rebbeck et al., 2016	F	BRCA1	c.3048_3052dup5 (p.Asn1018Metfs)	Frameshift	P3*	BRCA2	c.2830A > T (p.Lys944Ter)	Nonsense	P3*	No cancer = 39.1y (20–68)	Recent	39.1	N/A	AVERAGE	N
52	Rebbeck et al., 2016	F	BRCA1	c.3228_3229delAG (p.Gly1077fs)	Frameshift	P3*	BRCA2	c.3689delC (p.Ser1230fs)	Frameshift	P3*	No cancer = 39.1y (20–68)	Recent	39.1	N/A	AVERAGE	N
53	Rebbeck et al., 2016	F	BRCA1	c.3228_3229delAG (p.Gly1077fs)	Frameshift	P3*	BRCA2	c.9253dupA (p.Thr3085Asnfs)	Frameshift	P3*	BC = 39.9y (23–67)	Recent	39.9	39.9	AVERAGE	N
54	Rebbeck et al., 2016	F	BRCA1	c.3400G > T (p.Glu1134Ter)	Nonsense	P3*	BRCA2	c.2808_2811delACAA	Frameshift	P3*	BC = 39.9y (23–67)	Recent	39.9	39.9	AVERAGE	N

								(p.Ala938Profs)								
55	Rebbeck et al., 2016	F	BRCA1	c.3400G > T (p.Glu1134Ter)	Nonsense	P3*	BRCA2	c.2808_2811delACAA (p.Ala938Profs)	Frameshift	P3*	No cancer = 39.1y (20–68)	Recent	39.1	N/A	AVERAGE	N
56	Rebbeck et al., 2016	F	BRCA1	c.3477_3480 delAAAG (p.Ile1159fs)	Frameshift	P3*	BRCA2	c.9401delG (p.Gly3134fs)	Frameshift	P2*	BC = 39.9y (23–67)	Recent	39.9	39.9	AVERAGE	N
57	Rebbeck et al., 2016	F	BRCA1	c.3627dupA	Frameshift	P3*	BRCA2	c.6724_6725delGA	Frameshift	P1*	BC = 39.9y (23–67)	Recent	39.9	39.9	AVERAGE	N
58	Rebbeck et al., 2016	F	BRCA1	c.3700_3704del5 (p.Val1234fs)	Frameshift	P3*	BRCA2	c.1815dupA (p.Pro606Thrfs)	Frameshift	P3*	BC = 39.9y (23–67)	Recent	39.9	39.9	AVERAGE	N
59	Rebbeck et al., 2016	F	BRCA1	c.3756_3759delGTCT	Frameshift	P3*	BRCA2	c.7757G > A (p.Trp2586Ter)	Nonsense	P3*	BC = 39.9y (23–67)	Recent	39.9	39.9	AVERAGE	N
60	Rebbeck et al., 2016	F	BRCA1	c.3759_3760delTA (p.Lys1254fs)	Frameshift	P3*	BRCA2	c.9699_9702 delTATG (p.Cys3233fs)	Frameshift	P3*	BC + OC = 41.9y (26–53)	Recent	41.9	41.9	AVERAGE	Y

6 1	Rebbe ck et al., 2016	F	BR CA 1	c.3770_377 1delAG	Frameshif t	P3*	BRC A2	c.5946delT	Frameshi ft	P3*	BC = 39.9y (23–67)	Recen t	39.9	39.9	AVE RAG E	N
6 2	Rebbe ck et al., 2016	F	BR CA 1	c.3770_377 1delAG	Frameshif t	P3*	BRC A2	c.5946delT	Frameshi ft	P3*	BC + OC = 41.9y (26–53)	Recen t	41.9	41.9	AVE RAG E	Y
6 3	Rebbe ck et al., 2016	F	BR CA 1	c.390C > A (p.Tyr130Te r)	Nonsense	P3*	BRC A2	c.3018delA (p.Gly1007f s)	Frameshi ft	P3*	BC = 39.9y (23–67)	Recen t	39.9	39.9	AVE RAG E	N
6 4	Rebbe ck et al., 2016	F	BR CA 1	3910delG (E1257fs)	Frameshif t	P3*	BRC A2	c.2830A > T (p.Lys944Te r)	Nonsens e	P3*	BC = 39.9y (23–67)	Recen t	39.9	39.9	AVE RAG E	N
6 5	Rebbe ck et al., 2016	F	BR CA 1	c.4035delA (p.Glu1346f s)	Frameshif t	P3*	BRC A2	c.658_659d elGT (p.Val220fs)	Frameshi ft	P3*	No cancer = 39.1y (20–68)	Recen t	39.1	N/A	AVE RAG E	N
6 6	Rebbe ck et al., 2016	F	BR CA 1	c.4065_406 8delTCAA (p.Asn1355f s)	Frameshif t	P3*	BRC A2	c.5350_535 1delAA (p.Asn1784f s)	Frameshi ft	P3*	BC = 39.9y (23–67)	Recen t	39.9	39.9	AVE RAG E	N
6 7	Rebbe ck et al., 2016	F	BR CA 1	c.4186?435 7+?dup	Unknown	P2*	BRC A2	c.2636_263 7delCT (p.Asp878_ Ser879Ter)	Nonsens e	P3*	BC = 39.9y (23–67)	Recen t	39.9	39.9	AVE RAG E	N
6 8	Rebbe ck et	F	BR CA 1	c.4186?435 7+?dup	Unknown	P2*	BRC A2	c.2636_263 7delCT	Nonsens e	P3*	No cancer = 39.1y (20–68)	Recen t	39.1	N/A	AVE RAG E	N

	al., 2016							(p.Asp878_ Ser879Ter)								
69	Rebbeck et al., 2016	F	BRCA1	c.427G > T (p.Glu143Ter)	Nonsense	P3*	BRCA2	c.8730delT (p.Asn2910fs)	Frameshift	P3*	BC = 39.9y (23–67)	Recent	39.9	39.9	AVERAGE	N
70	Rebbeck et al., 2016	F	BRCA1	c.5030_5033 delCTAA (p.Thr1677fs)	Frameshift	P3*	BRCA2	c.1399A > T (p.Lys467Ter)	Nonsense	P3*	BC = 39.9y (23–67)	Recent	39.9	39.9	AVERAGE	N
71	Rebbeck et al., 2016	F	BRCA1	c.5123C > A (p.Ala1708Glu)	Missense	P3*	BRCA2	c.6275_6276delTT (p.Leu2092fs)	Frameshift	P3*	BC = 39.9y (23–67)	Recent	39.9	39.9	AVERAGE	N
72	Rebbeck et al., 2016	F	BRCA1	c.5136G > A (p.Trp1712Ter)	Nonsense	P3*	BRCA2	c.4965delC (p.Cys1654_Tyr1655insTer)	Nonsense	P3*	BC = 39.9y (23–67)	Recent	39.9	39.9	AVERAGE	N
73	Rebbeck et al., 2016	F	BRCA1	c.5193 + 1delG (IVS18+1delG)	Splice Donor	P2*	BRCA2	c.658_659delGT (p.Val220fs)	Frameshift	P3*	BC = 39.9y (23–67)	Recent	39.9	39.9	AVERAGE	N
74	Rebbeck et al., 2016	F	BRCA1	c.5266dupC (p.Gln1756Profs)	Frameshift	P3*	BRCA2	c.8364G > A (p.Trp2788Ter)	Nonsense	P3*	BC = 39.9y (23–67)	Recent	39.9	39.9	AVERAGE	N
75	Rebbeck et	F	BRCA1	c.5266dupC	Frameshift	P3*	BRCA2	c.5946delT	Frameshift	P3*	BC = 39.9y (23–67)	Recent	39.9	39.9	AVERAGE	N

	al., 2016			(p.Gln1756 Profs)												
76	Rebbeck et al., 2016	F	BRCA1	c.5266dupC (p.Gln1756 Profs)	Frameshift	P3*	BRCA2	c.5946delT	Frameshift	P3*	BC = 39.9y (23–67)	Recent	39.9	39.9	AVERAGE	N
77	Rebbeck et al., 2016	F	BRCA1	c.5266dupC (p.Gln1756 Profs)	Frameshift	P3*	BRCA2	c.5946delT	Frameshift	P3*	BC = 39.9y (23–67)	Recent	39.9	39.9	AVERAGE	N
78	Rebbeck et al., 2016	F	BRCA1	c.5266dupC (p.Gln1756 Profs)	Frameshift	P3*	BRCA2	c.5946delT	Frameshift	P3*	BC + OC = 41.9y (26–53)	Recent	41.9	41.9	AVERAGE	Y
79	Rebbeck et al., 2016	F	BRCA1	c.5266dupC (p.Gln1756 Profs)	Frameshift	P3*	BRCA2	c.5946delT	Frameshift	P3*	No cancer = 39.1y (20–68)	Recent	39.1	N/A	AVERAGE	N
80	Rebbeck et al., 2016	F	BRCA1	c.5266dupC (p.Gln1756 Profs)	Frameshift	P3*	BRCA2	c.4478_4481delAAAG (p.Glu1493fs)	Frameshift	P3*	BC = 39.9y (23–67)	Recent	39.9	39.9	AVERAGE	N
81	Rebbeck et al., 2016	F	BRCA1	c.5266dupC (p.Gln1756 Profs)	Frameshift	P3*	BRCA2	c.5645C > A (p.Ser1882Ter)	Nonsense	P3*	BC + OC = 41.9y (26–53)	Recent	41.9	41.9	AVERAGE	Y
82	Rebbeck et al., 2016	F	BRCA1	c.962G > A (p.Trp321Ter)	Nonsense	P3*	BRCA2	c.2231C > G (p.Ser744Ter)	Nonsense	P3*	BC = 39.9y (23–67)	Recent	39.9	39.9	AVERAGE	N

83	Rebbeck et al., 2016	F	BRCA1	c.212 + 1G > A	Splice Donor	P3*	BRCA2	c.739_740delAT*	Frameshift	P3*	BC = 39.9y (23–67)	Recent	39.9	39.9	AVERAGE	N
84	Rebbeck et al., 2016	F	BRCA1	c.3196G > T (p.Glu1066Ter)	Nonsense	P3*	BRCA2	c.658_659delGT (p.Val220fs)	Frameshift	P3*	BC = 39.9y (23–67)	Recent	39.9	39.9	AVERAGE	N
85	Rebbeck et al., 2016	F	BRCA1	c.3916_3917delTT (p.Leu1306fs)	Frameshift	P3*	BRCA2	c.5380delG* (p.Asn1793_Val1794insTer)	Frameshift	P3*	BC + OC = 41.9y (26–53)	Recent	41.9	41.9	AVERAGE	Y
86	Rebbeck et al., 2016	F	BRCA1	c.548-?_4185 + ?del	Large Deletion	P1*	BRCA2	c.2269A > T	Nonsense	P3*	BC + OC = 41.9y (26–53)	Recent	41.9	41.9	AVERAGE	Y
87	Na et al., 2017	M	BRCA1	c.5266dup; (p.Q1756Pfs*74)	Frameshift	P3*	BRCA2	c.5946del; (p.S1982Rfs*22)	Frameshift	P3*	PC 57y (localised) Biopsy Gleason Score: 6	Recent	57	N/A	EXACT	N
88	Spannuth et al., 2007	F	BRCA1	5382insC (p.Gln1756Profs)	Frameshift	P3*	BRCA2	6174delT (p.Ser1982fs)	Frameshift	P3*	IDC 46y bilateral OC 57y	Recent	46	46	EXACT	Y
89	Meynard et al., 2018	F	BRCA1	c.1016dupA (p.V340Gfs*6)	Frameshift	P3*	BRCA2	c.6814delA (p.R2272Efs*8)	Frameshift	P3*	BBC 46y (distinct histologies right/left)	Recent	46	46	EXACT	Y

											Right IDC; IHC = ER+, PR-, HER2 WT.<br><br>Left IDC; IHC = TripNeg					
90	Nomizu et al., 2015	F	BRCA1	c.188T>A<br><br>(p.Leu63Ter)	Frameshift	P3*	BRCA2	c.5576_5579del<br><br>(p.Ile1859fs)	Frameshift	P3*	BC 55y IHC = ER+ PR- HER2-, BRCA1- BRCA2-	Recent	55	55	EXACT	N
91	Vietri et al., 2013	F	BRCA1	c.547+2T>A;<br><br>(p.Gln148Aspfsx51)	Frameshift	P3*	BRCA2	c.2830A>T<br><br>(p.Lys944X)	Nonsense	P3*	TRIPLE HET: BRCA2 c.426-57A>G BBC 32y; IHC = ER+, PR+, HER2-	Recent	32	32	EXACT	Y
92	Vietri et al., 2013	F	BRCA1	c.547+2T>A;<br><br>(p.Gln148Aspfsx51)	Frameshift	P3*	BRCA2	c.2830A>T<br><br>(p.Lys944X)	Nonsense	P3*	TRIPLE HET: BRCA2 c.426-57A>G BC 39y	Recent	39	39	EXACT	N
93	Vietri et al., 2013	M	BRCA1	c.547+2T>A;<br><br>(p.Gln148Aspfsx51)	Frameshift	P3*	BRCA2	c.2830A>T<br><br>(p.Lys944X)	Nonsense	P3*	TRIPLE HET: BRCA2 c.426-57A>G No symptoms 71y	Recent	71	N/A	EXACT	N

94	Lavie et al., 2011	F	BRCA1	185delAG (p.Glu23fs)	Frameshift	P3*	BRCA2	6174delT (p.Ser1982fs)	Frameshift	P3*	BC = 49.3y (+-17)	Recent	49.3	49.3	AVERAGE	N
95	Lavie et al., 2011	F	BRCA1	185delAG (p.Glu23fs)	Frameshift	P3*	BRCA2	6174delT (p.Ser1982fs)	Frameshift	P3*	BC = 49.3y (+-17)	Recent	49.3	49.3	AVERAGE	N
96	Lavie et al., 2011	F	BRCA1	185delAG (p.Glu23fs)	Frameshift	P3*	BRCA2	6174delT (p.Ser1982fs)	Frameshift	P3*	BC = 49.3y (+-17)	Recent	49.3	49.3	AVERAGE	N
97	Lavie et al., 2011	F	BRCA1	185delAG (p.Glu23fs)	Frameshift	P3*	BRCA2	6174delT (p.Ser1982fs)	Frameshift	P3*	BC = 49.3y (+-17)	Recent	49.3	49.3	AVERAGE	N
98	Lavie et al., 2011	F	BRCA1	185delAG (p.Glu23fs)	Frameshift	P3*	BRCA2	6174delT (p.Ser1982fs)	Frameshift	P3*	BC = mean 47.0y (+/- 13.5)	Recent	47	47	AVERAGE	N
99	Lavie et al., 2011	F	BRCA1	185delAG (p.Glu23fs)	Frameshift	P3*	BRCA2	6174delT (p.Ser1982fs)	Frameshift	P3*	BC = mean 47.0y (+/- 13.5)	Recent	47	47	AVERAGE	N
100	Lavie et al., 2011	F	BRCA1	185delAG (p.Glu23fs)	Frameshift	P3*	BRCA2	6174delT (p.Ser1982fs)	Frameshift	P3*	BC = mean 47.0y (+/- 13.5)	Recent	47	47	AVERAGE	N
101	Lavie et al., 2011	F	BRCA1	185delAG (p.Glu23fs)	Frameshift	P3*	BRCA2	6174delT (p.Ser1982fs)	Frameshift	P3*	BC = mean 47.0y (+/- 13.5)	Recent	47	47	AVERAGE	N

102	Lavie et al., 2011	F	BRCA1	185delAG (p.Glu23fs)	Frameshift	P3*	BRCA2	6174delT (p.Ser1982fs)	Frameshift	P3*	BC = mean 47.0y (+/- 13.5)	Recent	47	47	AVERAGE	N
103	Lavie et al., 2011	F	BRCA1	185delAG (p.Glu23fs)	Frameshift	P3*	BRCA2	6174delT (p.Ser1982fs)	Frameshift	P3*	BC = mean 47.0y (+/- 13.5)	Recent	47	47	AVERAGE	N
104	Lavie et al., 2011	F	BRCA1	185delAG (p.Glu23fs)	Frameshift	P3*	BRCA2	6174delT (p.Ser1982fs)	Frameshift	P3*	OC = y?	Recent	N/A	N/A	UNKNOWN	N
105	Lavie et al., 2011	F	BRCA1	185delAG (p.Glu23fs)	Frameshift	P3*	BRCA2	6174delT (p.Ser1982fs)	Frameshift	P3*	No Cancer y?	Recent	N/A	N/A	UNKNOWN	N
106	Lavie et al., 2011	F	BRCA1	185delAG (p.Glu23fs)	Frameshift	P3*	BRCA2	6174delT (p.Ser1982fs)	Frameshift	P3*	No Cancer y?	Recent	N/A	N/A	UNKNOWN	N
107	Lavie et al., 2011	F	BRCA1	185delAG (p.Glu23fs)	Frameshift	P3*	BRCA2	6174delT (p.Ser1982fs)	Frameshift	P3*	No Cancer y?	Recent	N/A	N/A	UNKNOWN	N
108	Lavie et al., 2011	F	BRCA1	185delAG (p.Glu23fs)	Frameshift	P3*	BRCA2	6174delT (p.Ser1982fs)	Frameshift	P3*	No Cancer y?	Recent	N/A	N/A	UNKNOWN	N
109	Lavie et al., 2011	F	BRCA1	185delAG (p.Glu23fs)	Frameshift	P3*	BRCA2	6174delT (p.Ser1982fs)	Frameshift	P3*	No Cancer y?	Recent	N/A	N/A	UNKNOWN	N

110	Lavie et al., 2011	F	BRCA1	185delAG (p.Glu23fs)	Frameshift	P3*	BRCA2	6174delT (p.Ser1982fs)	Frameshift	P3*	No Cancer y?	Recent	N/A	N/A	UNKNOWN	N
111	Lavie et al., 2011	F	BRCA1	185delAG (p.Glu23fs)	Frameshift	P3*	BRCA2	6174delT (p.Ser1982fs)	Frameshift	P3*	No Cancer y?	Recent	N/A	N/A	UNKNOWN	N
112	Lavie et al., 2011	M	BRCA1	185delAG (p.Glu23fs)	Frameshift	P3*	BRCA2	6174delT (p.Ser1982fs)	Frameshift	P3*	No Cancer y?	Recent	N/A	N/A	UNKNOWN	N
113	Lavie et al., 2011	M	BRCA1	185delAG (p.Glu23fs)	Frameshift	P3*	BRCA2	6174delT (p.Ser1982fs)	Frameshift	P3*	No Cancer y?	Recent	N/A	N/A	UNKNOWN	N
114	Lavie et al., 2011	M	BRCA1	185delAG (p.Glu23fs)	Frameshift	P3*	BRCA2	6174delT (p.Ser1982fs)	Frameshift	P3*	No Cancer y?	Recent	N/A	N/A	UNKNOWN	N
115	Lavie et al., 2011	M	BRCA1	185delAG (p.Glu23fs)	Frameshift	P3*	BRCA2	6174delT (p.Ser1982fs)	Frameshift	P3*	No Cancer y?	Recent	N/A	N/A	UNKNOWN	N
116	Lavie et al., 2011	F	BRCA1	5382insC (p.Gln1756Profs)	Frameshift	P3*	BRCA2	6174delT (p.Ser1982fs)	Frameshift	P3*	BC = mean 37.5 (+/-13.5)	Recent	37.5	37.5	AVERAGE	N
117	Lavie et al., 2011	F	BRCA1	5382insC (p.Gln1756Profs)	Frameshift	P3*	BRCA2	6174delT (p.Ser1982fs)	Frameshift	P3*	BC = mean 37.5 (+/-13.5)	Recent	37.5	37.5	AVERAGE	N

118	Lavie et al., 2011	M	BRCA1	5382insC (p.Gln1756Profs)	Frameshift	P3*	BRCA2	6174delT (p.Ser1982fs)	Frameshift	P3*	No Cancer y?	Recent	N/A	N/A	UNKNOWN	N
119	Lavie et al., 2011	M	BRCA1	5382insC (p.Gln1756Profs)	Frameshift	P3*	BRCA2	6174delT (p.Ser1982fs)	Frameshift	P3*	No Cancer y?	Recent	N/A	N/A	UNKNOWN	N
120	Koren-Michowitz et al., 2005	F	BRCA1	(p.Glu23fs) or (p.Gln1756Profs)	Frameshift	P3*	BRCA2	(p.Ser1982fs)	Frameshift	P3*	TRIPLE HET: Fanconi anaemia - *FANCC c.456+4A>T 2* pathogenic 33y no cancer history	Recent	33	N/A	AVERAGE	N
121	Koren-Michowitz et al., 2005	F	BRCA1	(p.Glu23fs) or (p.Gln1756Profs)	Frameshift	P3*	BRCA2	(p.Ser1982fs)	Frameshift	P3*	TRIPLE HET: BLOOM SYNDROME - BLM c.2207_2212delinsTAGATTC (p.Tyr736fs) 2* pathogenic BC 45y	Recent	45	45		N

122	Koren-Michowitz et al., 2005	F	BRCA1	(p.Glu23fs) or (p.Gln1756Profs)	Frameshift	P3*	BRCA2	(p.Ser1982fs)	Frameshift	P3*	BC 38y (SD +/- 9y) Inferred to be 32y as above case (#99) is 45y	Recent	38	38		N
123	Loader and Rowley, 1998	M	BRCA1	E1694X	Nonsense	P3*	BRCA2	6633del5	Frameshift	P3*	"in his 30's" with no known cancer	Recent	35	N/A	AVERAGE	N
124	Loader and Rowley, 1998	M	BRCA1	E1694X	Nonsense	P3*	BRCA2	6633del5	Frameshift	P3*	"in his 30's" with no known cancer	Recent	35	N/A	AVERAGE	N
125	Loader and Rowley, 1998	F	BRCA1	E1694X	Nonsense	P3*	BRCA2	6633del5	Frameshift	P3*	"in her 30's" with no known cancer	Recent	35	N/A	AVERAGE	N
126	Steffensen et al., 2010	F	BRCA1	c.5096G > A (p.Arg1699Gln)	Missense	P3*	BRCA2	c.631 + 4A>G	Splice site Variant	P/LP2*	BC 53y OC 59y	Recent	53	53	EXACT	Y
127	Steffensen et al., 2010	F	BRCA1	c.5096G > A (p.Arg1699Gln)	Missense	P3*	BRCA2	c.631 + 4A>G	Splice site Variant	P/LP2*	No known cancer y?; <59 (daughter of #104)	Recent	N/A	N/A	UNKNOWN	N

128	Steffensen et al., 2010	M	BRCA1	c.5096G > A (p.Arg1699Gln)	Missense	P3*	BRCA2	c.631 + 4A>G	Splice site Variant	P/LP2*	BC 76y	Recent	76	76	EXACT	N
129	Steffensen et al., 2010	M	BRCA1	c.5096G > A (p.Arg1699Gln)	Missense	P3*	BRCA2	c.631 + 4A>G	Splice site Variant	P/LP2*	No known cancer y?; <59 (Son of #104)	Recent	N/A	N/A	UNKNOWN	N
130	Claus et al., 2005	F	BRCA1	W321X	Nonsense	P3*	BRCA2	3398del5	Frameshift	P3*	BC 37y	Recent	37	37	EXACT	N
131	Nomizu et al., 2015	F	BRCA1	L63X	Nonsense	P3*	BRCA2	5804del4	Frameshift	P3*	BC 41y EnC 46y	Recent	41	41	EXACT	Y
132	Tedaldi et al., 2017	F	BRCA1	c.5266dupC (p.Gln1756fs)	Frameshift	P3*	BRCA2	c.2049_2050del (p.Ile684fs)	Frameshift	P3*	IDC 53y	Recent	53	53	EXACT	N
133	Nurmi et al., 2019	F	BRCA1	c.3485delA	Frameshift	P3*	ATM	c.6908dupA	Frameshift	P2*	TRIPLE HET; RAD51C: c.93delG2* Pathogenic, Frameshift OC 60y (high-grade serous)	Recent	60	N/A	EXACT	N
134	Nurmi et al., 2019	F	BRCA1	c.3485delA	Frameshift	P3*	ATM	c.6908dupA	Frameshift	P2*	OC (Endometrioid) 53y	Recent	53	N/A	EXACT	N

135	Nurmi et al., 2019	F	BRCA1	c.3485delA	Frameshift	P3*	FANCM	c.5101C>T	Nonsense	LP2*	OC 42y (clear cell)	Recent	42	N/A	EXACT	N
136	Nurmi et al., 2019	F	BRCA1	c.4097-2A>G	Splice Acceptor	P3*	FANCM	c.5101C>T	Nonsense	LP2*	IDC 60.1y; IHC = ER+ PR+ HER2?	Recent	60.1	60.1	EXACT	N
137	Nurmi et al., 2019	F	BRCA1	c.4327C>T	Nonsense	P3*	CHEK2	c.1100delC	Frameshift	1*Conflict	IDC 49.0y, BBC 55.8y; IHC = ER+ PR? HER2?	Recent	49	49	EXACT	Y
138	Nurmi et al., 2019	F	BRCA1	c.4327C>T	Nonsense	P3*	FANCM	c.5101C>T	Nonsense	LP2*	IDC 37.2y; IHC = Trip Neg<br><br>OC 40.7y	Recent	37.2	37.2	EXACT	Y
139	Nurmi et al., 2019	F	BRCA1	c.5266dupC	Frameshift	P3*	FANCM	c.5101C>T	Nonsense	LP2*	IDC 48.8y; IHC = Trip Neg	Recent	48.8	48.8	EXACT	N
140	Nurmi et al., 2019	F	BRCA2	c.771_775delTCAAA	Frameshift	P3*	ATM	c.7570G>C	Missense	LP2*	IDC 76.6y; IHC = ER+ PR- HER2?	Recent	76.6	76.6	EXACT	N
141	Nurmi et al., 2019	F	BRCA2	c.7480C>T	Nonsense	P3*	PALB2	c.1592delT	Frameshift	P2*	DCIS + BBC 38.5y + 52.4y; IHC = ER+ PR+ HER2?	Recent	38.5	38.5	EXACT	Y
142	Nurmi et al., 2019	F	BRCA2	c.7480C>T	Nonsense	P3*	CHEK2	c.1100delC	Frameshift	1*Conflict	DCIS 61.7y; IHC = ER- PR- HER2+	Recent	61.7	61.7	EXACT	N

143	Nurmi et al., 2019	F	BRCA2	c.8327T>G	Nonsense	P3*	FANCM	c.5101C>T	Nonsense	LP2*	IDC 36.4y; IHC = ER+ PR+ HER2-	Recent	36.4	36.4	EXACT	N
144	Nurmi et al., 2019	F	BRCA2	c.8327T>G	Nonsense	P3*	FANCM	c.5101C>T	Nonsense	LP2*	LBC, BBC 53.2y + 77.3y; IHC = ER? PR+ HER2?<br><br>OC 82y	Recent	53.2	53.2	EXACT	Y
145	Nurmi et al., 2019	F	BRCA2	c.9118-2A>G	Splice Acceptor	P3*	FANCM	c.5101C>T	Nonsense	LP2*	IDC, BBC 53.5y + 59.6y; IHC = ER+ PR+ HER2?	Recent	53.5	53.5	EXACT	Y
146	Stradella et al., 2019	F	BRCA1	c.3607C>T p.(Arg1203*)	Nonsense	P3*	TP53	c.659A>G p.(Tyr220Cys)	Unknown	P3*	OC 45y<br><br>Phenotype = HBOC	Recent	45	N/A	EXACT	N
147	Sokolenko et al., 2014	F	BRCA1	5382insC	Frameshift	P3*	BLM	Q548X	Nonsense	P2*	OC 50y<br><br>BC 68y IHC = Trip neg	Recent	50	68	EXACT	Y
148	Sokolenko et al., 2014	F	BRCA1	5382insC	Frameshift	P3*	BLM	Q548X	Nonsense	P2*	Waldenstrom disease 58y Δ<br><br>BC 67y; IHC = Trip neg	Recent	58	67	EXACT	Y

149	Sokolenko et al., 2014	F	BRCA1	C61G	Missense	P3*	ATM	E1978X	Missense	P2*	BC 44y; IHC = ER+, PR? HER2?	Recent	44	44	EXACT	N
150	Sokolenko et al., 2014	F	BRCA1	C61G	Missense	P3*	ATM	E1978X	Missense	P2*	BC 40y ; IHC = ER-, PR? HER2?	Recent	40	40	EXACT	N
151	Sokolenko et al., 2014	F	BRCA1	5382insC	Frameshift	P3*	BLM	Q548X	Nonsense	P2*	BC 42y; IHC = ER-, PR? HER2?	Recent	42	42	EXACT	N
152	Sokolenko et al., 2014	F	BRCA1	C61G	Missense	P3*	CHEK2	c.1100delC	Frameshift	1*Conflict	BC 45y ; IHC = ER-, PR? HER2?	Recent	45	45	EXACT	N
153	Sokolenko et al., 2014	F	BRCA1	5382insC	Frameshift	P3*	CHEK2	c.444+1G>A<br><br>(IVS2+1G>A)	Splice Donor	P/LP2*	BC 58y; IHC = ER+, PR? HER2?	Recent	58	58	EXACT	N
154	Sokolenko et al., 2014	F	CHEK2	1100delC	Frameshift	1*Conflict	BLM	Q548X	Nonsense	P2*	BC = 54y; IHC = Trip Neg	Recent	54	54	EXACT	N
155	Andrés et al., 2019	F	BRCA1	c.5123C>A (p.Ala1708Glu)	Missense	P3*	ATM	c.2413C>T<br><br>(p.Arg805X)	Nonsense	P2*	BC 55y; IHC = Trip Neg<br><br>Jaundice + elevated transaminases;	Recent	55	55	EXACT	Y

											Pancreas apudoma adenocarcinoma 62y<br><br>Endometrial Cancer 71y					
156	Ouyang et al., 2019	F	BRCA1	c.5095C>T (p.Arg1699Trp)	Missense	P3*	PALB2	c.1059del<br><br>p.K353Nfs*3	Frameshift/<br><br>Nonsense	P/LP2*	BC 42y; IHC = Trip Neg	Recent	42	42	EXACT	N
157	Cote et al., 2012	F	BRCA2	c.9004G>A<br><br>(E3002K)	Missense	P/LP2*	PALB2	c.2323C>T<br><br>(Q775X)	Nonsense	P2*	BC 45y<br><br>Family History: Excess of colorectal cancers	Recent	45	45	EXACT	N
158	Tedaldi et al., 2017	F	BRCA2	c.6998dupT<br><br>(p.Pro2334fs)	Frameshift	P3*	FANCA	c.987_990del<br><br>(p.Thr329fs)	Frameshift	P2*	IDC 30y	Recent	30	30	EXACT	N
159	Tedaldi et al., 2017	F	BRCA2	c.658_659delGT<br><br>(p.Val220fs)	Frameshift	P3*	BRIP1	c.2992_2995del (p.Lys998fs)	Frameshift	P2*	IDC x2 37y	Recent	37	37	EXACT	Y
160	Tedaldi et al., 2017	F	BRCA2	c.8487+1G>A	Splice Donor	P3*	ATM	c.3275C>A (p.Ser1092Ter)	Nonsense	P1*	DCIS 38y<br><br>IDC 45y	Recent	38	38	EXACT	Y
161	Momozawa	F	BRCA2	p.Ala938fs	Frameshift	P3*	ATM	c.72+1G>A	Splice Donor	P/LP2*	IDC 45y; IHC = ER+, PR+, HER2-	Recent	45	45	EXACT	N

	et al., 2018														
162	Momozawa et al., 2018	F	BRCA2	p.Arg3128*	Frameshift	P3*	CHEK2	c.573+1delG	Splice Donor	P1*	IDC 56y; IHC = ER+, PR-, HER2?	Recent	56	56	EXACT	N
163	Sarkadi et al., 2019	F	BRCA1	p.Ile90Serfs	Frameshift	P3*	RET	p.Cys634Trp	Missense	P1*	Medullary Thyroid Carcinoma 16y	Recent	16	N/A	EXACT	N
164	Fostira et al., 2019	F	BRCA1	c.5497G>A (p.Val1833Met)	Missense	P/LP2*	PALB2	c.2257C>T (p.Arg753Ter)	Nonsense	P2*	BC 44y	Recent	44	44	EXACT	N
165	Fostira et al., 2019	F	BRCA2	c.2490_2491insT (p.Val831Cysfs)	Frameshift	P3*	RAD51C	c.904+5G>T	Unknown	LP2*	BC 46y BC 56y	Recent	46	46	EXACT	Y
166	Fostira et al., 2019	F	BRCA2	c.7879A>T (p.Ile2627Phe)	Missense	P3*	SDHC	c.397C>T (p.Arg133Ter)	Nonsense	P2*	BBC 31y	Recent	31	31	EXACT	Y
167	Nurmi et al., 2019	F	BRCA2	c.7480C>T	Nonsense	P3*	CHEK2	c.1100delC	Frameshift	1*Conflict	TRIP HET; FANCM: c.5101C>T LP2*, nonsense IDC 55.4y; IHC = ER+ PR+ HER2-	Recent	55.4	55.4	EXACT	N

168	Sokolenko et al., 2014	F	BRCA1	5382insC	Frameshift	P3*	CHEK2	del5395 (c.909-?_1095+?del)	Frameshift	P2*	BC 52y<br><br>IHC = ER+, PR+, HER2-	Recent	52	52	EXACT	N
169	Nurmi et al., 2019	F	PALB2	c.1592delT	Frameshift	P2*	FANCM	c.5101C>T	Nonsense	LP2*	IDC 33.4y; IHC = ER+ PR+ HER2+	Recent	33.4	33.4	EXACT	N
170	Nurmi et al., 2019	F	PALB2	c.1592delT	Frameshift	P2*	FANCM	c.5101C>T	Nonsense	LP2*	IDC 55y; IHC = Trip Neg	Recent	55	55	EXACT	N
171	Nurmi et al., 2019	F	CHEK2	c.1100delC	Frameshift	1*Conflict	ATM	c.6908dupA	Frameshift	P2*	IDC 53.2y; IHC = ER+ PR- HER2- Bladder cancer 58y	Recent	53.2	53.2	EXACT	Y
172	Nurmi et al., 2019	F	CHEK2	c.1100delC	Frameshift	1*Conflict	ATM	c.7570G>C	Missense	LP2*	IDC 60y; IHC = ER+ PR+ HER2-	Recent	60	60	EXACT	N
173	Nurmi et al., 2019	F	CHEK2	c.1100delC	Frameshift	1*Conflict	FANCM	c.5101C>T	Nonsense	LP2*	LBC 46y; IHC = ER+ PR+ HER2 -	Recent	46	46	EXACT	N
174	Nurmi et al., 2019	F	CHEK2	c.1100delC	Frameshift	1*Conflict	FANCM	c.5101C>T	Nonsense	LP2*	IDC 87.5y; IHC = ER+ PR+ HER2-	Recent	87.5	87.5	EXACT	N
175	Nurmi et al., 2019	F	ATM	c.6908dupA	Frameshift	P2*	FANCM	c.5101C>T	Nonsense	LP2*	OC 46y (mucinous)	Recent	46	N/A	EXACT	N
176	Stradella et al., 2019	F	MLH1	c.244A>G; p.(Thr82Ala)	Missense	LP3*	MEN1	c.784-9G>A	Spice Site	P2*	Hyperplasia 29y, Pituitary adenoma 36y	Recent	36	N/A	EXACT	Y

											Parathyroid tumour 39y, Neuroendocrine tumour 41y<br><br>Hepatic haemangiomas<br><br>Phenotype = MEN1					
177	Stradella et al., 2019	F	MLH1	c.244A>G; p.(Thr82Ala)	Missense	LP3*	MEN1	c.784-9G>A	Spice Site	P2*	Uterine carcinoma 41y, Colorectal cancer 48y<br><br>Haemangiomas 45y, Hyperparathyroidism 45y<br><br>Hepatic haemangiomas<br><br>Phenotype =	Recent	41	N/A	EXACT	Y

											MEN1,<br>HNPCC					
1<br>7<br>8	Stradel<br>la et<br>al.,<br>2019	F	TSC<br>2	c.5227C>T;<br>p.(Arg1743<br>Trp)	Missense	P2*	RA<br>D5<br>1D	c.694C>T<br>p.(Arg232*)	Nonsens<br>e	P2*	Subcutaneo<br>us benign<br>tumours 6y<br><br>Epilepsy<br><br>Phenotype =<br>Tuberous<br>sclerosis	Recen<br>t	6	N/A	EXA<br>CT	N
1<br>7<br>9	Stradel<br>la et<br>al.,<br>2019	F	PAL<br>B2	c.3256 C>T;<br>p.(Arg1086<br>*)	Nonsense	P/LP<br>2*	AT<br>M	c.3802delG<br><br>p.(Val1268*<br>)	Nonsens<br>e	P2*	BC 54y<br><br>Pancreatic<br>cancer 59y<br><br>Phenotype =<br>HBOC	Recen<br>t	54	54	EXA<br>CT	Y
1<br>8<br>0	Stradel<br>la et<br>al.,<br>2019	F	SD<br>HB	c.505C>T<br><br>p.(Gln169*)	Nonsense	P1*	FA<br>NC<br>A	c.3558dupG<br><br>p.(Arg1187<br>Glufs*28)	Frameshi<br>ft/<br><br>Nonsens<br>e	P2*	OC 49y<br><br>Phenotype =<br>HBOC	Recen<br>t	49	N/A	EXA<br>CT	N
1<br>8<br>1	Njorog<br>e et<br>al.,<br>2017	F	CD<br>H1	c.2287G>T	Nonsense	P/LP<br>2*	PM<br>S2	c.2445+1G><br>T	Splice<br>Donor	P2*	LBC 51y<br>Follicular<br>adenoma(th<br>yroid) +<br>micropapilla<br>ry<br>carcinoma<br>52y<br>Phenotype =<br>HDGC +	Recen<br>t	51	51	EXA<br>CT	Y

											Lynch syndrome					
182	Sokolenko et al., 2014	F	NBN	657del5	Frameshift	P2*	BLM	Q548X	Nonsense	P2*	BC 51y; IHC = ER+, PR+, HER2-	Recent	51	51	EXACT	N
183	Sokolenko et al., 2014	F	CHEK2	c.444+1G>A	Splice Donor	P/LP 2*	NBN	657del5	Frameshift	P2*	BC 48y; IHC = ER-, PR+, HER2+	Recent	48	48	EXACT	N
184	Vahteristo et al., 2001	F	MSH6	c.2983G>T (p.Glu995Ter)	Nonsense	P3*	CHEK2	c.1100del (p.Thr367fs)	Frameshift	1*Conflict	BC 34y<br>CRC 34y	Recent	34	34	EXACT	Y
185	Brown et al., 2020	M	MEN1	c0.525_526 insTT (p.Ala176Leufs*10)	Frameshift/<br>Nonsense	P1*	RET	c.1889G>A (p.Cys630Tyr)	Missense	P1*	Medullary Thyroid Carcinoma 27y; IHC = calcitonin +	Recent	27	N/A	EXACT	N
186	Sokolenko et al., 2014	F	CHEK2	c.444+1G>A	Splice Donor	P/LP 2*	NBN	657del5	Frameshift	P2*	BC 53y	Recent	53	53	EXACT	N
187	Sokolenko et al., 2014	F	CHEK2	c.444+1G>A	Splice Donor	P/LP 2*	NBN	657del5	Frameshift	P2*	BBC 59/60y; IHC = ER+, PR+, HER2+	Recent	59	59	EXACT	Y

188	Sokolenko et al., 2014	F	CHEK2	c.444+1G>A	Splice Donor	P/LP2*	NBN	657del5	Frameshift	P2*	BC 53y; IHC = ER+, PR-, HER2?	Recent	53	53	EXACT	N
189	Pearlman et al., 2017	F	MSH2	c.2388delT (p.V797Lfs*15)	Frameshift	P3*	MUTYH	c.1187G>A (p.G396D)	Missense	P/LP2*	30y CRC; MMR deficient Extensive family history of cancers: Father colon 60; Pat. halfsister 1 endometrial 54; Pat. half-sister 2 thyroid 39; Pat. aunt melanoma 60s; Mat. aunt breast 47; Mat. grandmother brain 52	Recent	30	N/A	EXACT	N
190	Yamamoto et al., 2006	M	PTPN11	c.182A>G (p.Asp61Gly)	Missense	P2*	FLT3/ITD	Data not avaliable	Data not avaliable	N/A	ALL 5y survival > 154 months	Recent	5	N/A	EXACT	N

191	Pearlman et al., 2017	F	ATM	c.7271T>G (p.V2424G)	Missense	P/LP2*	CHEK2	c.1100del	Frameshift	1*Conflict	CRC 44y; MMR proficient tumour Family History: Father prostate 74; Mother breast 50s; Mat. grandmother breast 50s	Recent	44	N/A	EXACT	N
192	Rebbeck et al., 2016	F	BRCA1	c.3155delA (3274delA)	Frameshift	P3*	BRCA2	c.3160_ 3163delGATA	Frameshift	N/A	BC = 39.9y (23–67)	Recent	39.9	39.9	AVERAGE	N
193	Rebbeck et al., 2016	F	BRCA1	c.3155delA (3274delA)	Frameshift	P3*	BRCA2	c.3160_ 3163delGATA	Frameshift	N/A	No cancer = 39.1y (20–68)	Recent	39.1	N/A	AVERAGE	N
194	Rebbeck et al., 2016	F	BRCA1	c.3700_3704del5 (p.Val1234fs)	Frameshift	P3*	BRCA2	c.681 + 1G > A	Splice Site Acceptor	N/A	BC = 39.9y (23–67)	Recent	39.9	39.9	AVERAGE	N
195	Rebbeck et al., 2016	F	BRCA1	c.3839_3843 delinsAGGC	Frameshift	N/A	BRCA2	c.1636delT (p.Cys546fs)	Frameshift	P3*	OC = 59.2y (57–62)	Recent	59.2	N/A	AVERAGE	N
196	Rebbeck et	F	BRCA1	c.3839_3843 delinsAGGC	Frameshift	N/A	BRCA2	c.1636delT (p.Cys546fs)	Frameshift	P3*	No cancer = 39.1y (20–68)	Recent	39.1	N/A	AVERAGE	N

	al., 2016															
197	Rebbeck et al., 2016	F	BRCA1	c.5251C > T (p.Arg1751Ter)	Nonsense	P3*	BRCA2	c.6753_6754delTT	Frameshift	N/A	BC = 39.9y (23–67)	Recent	39.9	39.9	AVERAGE	N
198	Rebbeck et al., 2016	F	BRCA1	c.5406 + 664_*8273del	Unknown	N/A	BRCA2	c.9748dupT (p.Ser3250fs)	Frameshift	P3*	BC = 39.9y (23–67)	Recent	39.9	39.9	AVERAGE	N
199	Ouyang et al., 2019	F	BRCA1	p.T1677Ifs*2	Frameshift/ Nonsense	N/A	BRCA2	c.631+1G>A	Splice Site	P/LP2*	BC 35y; IHC = Trip Neg	Recent	35	35	EXACT	N
200	Ouyang et al., 2019	F	BRCA1	p.V1120Dfs*11	Frameshift/ Nonsense	N/A	BRCA2	p.D687*fs*1	Frameshift/ Nonsense	N/A	Unknown Primary, y?	Recent	N/A	N/A	UNKNOWN	N
201	Fostira et al., 2019	F	BRCA1	c.5406 + 664_*8273del	Unknown	N/A	BRCA2	c.9748dupT (p.Ser3250fs)	Frameshift	P3*	BC 31y; IHC = Trip Neg<br>BC 49y; IHC = ?	Recent	31	31	EXACT	Y
202	Ng et al., 2016	F	BRCA2	c.5164_5465delAG	Frameshift	N/A	BARD1	c.1487C>G (S496X)	Nonsense	P1*	IDC 35y; IHC = ER+ PR+ HER2-	Recent	35	35	EXACT	N
203	Nurmi et al., 2019	F	BRCA2	c.7480C>T	Nonsense	P3*	FANCM	c.5791C>T	Nonsense	1* Conflict	IDC 46.2y; IHC = Trip Neg	Recent	46.2	N/A	EXACT	N

204	Stradella et al., 2019	F	BRCA1	c.1961delA p.(Lys654Serfs*47)	Frameshift/ Nonsense	P3*	APC	c.423-3T>A	Splice Site	1*Conflict	Colorectal polyposis 76y Phenotype = AFAP	Recent	76	N/A	EXACT	N
205	Stradella et al., 2019	F	BRCA1	c.2309C>A p.(Ser770*)	Nonsense	P3*	XPA	c.553C>T p.(Gln185*)	Nonsense	N/A	OC 51y Phenotype = HBOC	Recent	51	N/A	EXACT	N
206	Stradella et al., 2019	F	BRCA1	c.2309C>A p.(Ser770*)	Nonsense	P3*	XPA	c.553C>T p.(Gln185*)	Nonsense	N/A	OC 37y Phenotype = HBOC	Recent	37	N/A	EXACT	N
207	Sokolenko et al., 2014	F	BRCA1	c.3247del5	Frameshift	N/A	CHEK2	del5395 (c.909-?_1095+?del)	Frameshift	P2*	BC = 42y	Recent	42	N/A	EXACT	N
208	Bell et al., 2014	F	BRCA1	c.81-?_134 + ? del (p.Cys27*)	Nonsense	N/A	TP53	c.375 + 2T > C	Splice Site	N/A	20y metaplastic carcinoma + IDC (BBC) IHC = Both Trip Neg	Recent	20	20	EXACT	Y
209	Ouyang et al., 2019	F	BRCA1	p.K463*	Nonsense	N/A	PALB2	c.751C>T (p.Gln251Ter)	Nonsense	P/LP 2*	BC 35y; IHC = Trip Neg	Recent	35	35	EXACT	N
210	Ouyang et al., 2019	M	BRCA2	p.A2825Vfs*15	Frameshift/	N/A	PALB2	p.Q158Rfs*19	Frameshift/	N/A	PC 48y	Recent	48	N/A	EXACT	N

					Nonsense				Nonsense							
211	Ouyang et al., 2019	F	BRCA1	p.V757Ffs*8	Frameshift/ Nonsense	P3*	ATM	c.-30-1G>A	Splice Site	N/A	BC 47y; IHC = Trip Neg	Recent	47	47	EXACT	N
212	Ouyang et al., 2019	F	BRCA1	p.H399Tfs*2	Frameshift/ Nonsense	N/A	MSH6	p.R495*	Nonsense	P3*	OC 43y	Recent	43	N/A	EXACT	N
213	Ouyang et al., 2019	F	BRCA2	p.A1193Lfs*4	Frameshift/ Nonsense	N/A	PMS2	p.Q714*	Nonsense	P2*	BC 64y; IHC = Trip Neg	Recent	64	64	EXACT	N
214	Papi et al., 2009	F	BRCA1	c.3228_3229delAG (p.Gly1077fs)	Frameshift	P2*	MEN1	c.908delGCT	In-Frame Deletion	N/A	2x insulinoma 33y Mediastinal lipoma 35y hyperparathyroidism 32y, Hyperprolactinemia 33y	Recent	33	N/A	EXACT	Y
215	Sekido et al., 2017	F	BRCA2	Clinical Diagnosis	Missense	P?*	KIT	p.Trp557Leu	Missense	N/A	TRIP HET; KIT: p.Lys558Glu BC + GIST 65y; IHC = Trip Neg Family History = Uncle BC y?,	Recent	65	65	EXACT	Y

											mother BC 57y, sister OC 53y					
216	Pearlman et al., 2017	F	BRCA2	c.1755_1759del, (p.K585Nfs*3)	Frameshift/ Nonsense	P3*	CHEK2	c.751A>T (p.I251F)	Missense	VUS2*	CRC 47y; MMR proficient Site = rectum	Recent	47	N/A	EXACT	N
217	Tedaldi et al., 2017	F	BRCA1	c.5266dupC (p.Gln1756fs)	Frameshift	P3*	ERCC3	c.1757delA (p.Gln586fs)	Frameshift	VUS1*	IDC 39y	Recent	39	39	EXACT	N
218	Fostira et al., 2019	F	BRCA2	c.1813dupA (p.Ile605Asnfs)	Frameshift	P3*	RECQL4	c.1879-1G>A	Splice Site	N/A	BC 46y OC 63	Recent	46	46	EXACT	Y
219	Fostira et al., 2019	F	BRCA2	c.(80+1_81-1)_(593+1_594-1)del	Unknown	N/A	ATM	c.3576G>A (p.Lys1192=)	Synonymous (Splice site)	P2*	BC 40y	Recent	40	40	EXACT	N
220	Nurmi et al., 2019	F	ATM	c.7570G>C	Missense	LP2*	FANCM	c.5791C>T	Nonsense	1*Conflict	IDC, BBC 28y + 30.2y; IHC = ER+ PR+ HER2-	Recent	28	28	EXACT	Y
221	Balta et al., 2019	M	FANCA	c.1374delC	Frameshift	N/A	ATM	c.8977C>T	Missense	P/LP2*	Additional variant FANCA: c.1361-1370delCCTCCTTTGG	Recent	36	N/A	EXACT	N

											In-Frame Deletion No known cancer 36y					
222	Balta et al., 2019	F	FANCA	c.1374delC	Frameshift	N/A	ATM	c.8977C>T	Missense	P/LP2*	Additional variant FANCA: c.1361-1370delCCTCCTTTGG In-Frame Deletion No known cancer 36y	Recent	36	N/A	EXACT	N

223	Balta et al., 2019	M	FANCA	c.1374delC	Frameshift	N/A	ATM	c.8977C>T	Missense	P/LP 2*	Homozygous variant FANCA (CHILD OF 188 + 187) FA phenotype: microcephaly, growth retardation, cafe-au-lait spots, hyperpigmentation, bilateral microphthalmia, microcornea, severe malnutrition in the absence of any physical congenital skeletal abnormalities<br><br>presented at 9y with pancytopenia and macrocytosi	Recent	13	N/A	EXACT	N

											s<br><br>severe pancytopeni a and fever 13y, died with no signs of leukaemia					

224	Balta et al., 2019	F	FANCA	c.1374delC	Frameshift	N/A	ATM	c.8977C>T	Missense	P/LP 2*	Homozygous variant FANCA (CHILD OF 188 + 187) FA phenotype: microcephaly, growth retardation, cafe-au-lait spots, hyperpigmentation, bilateral microphthalmia, microcornea, severe malnutrition in the absence of any physical congenital skeletal abnormalities<br><br>mild pancytopenia and macrocytosis 8y, no sign	Recent	16	N/A	EXACT	N

											of leukaemia<br><br>Died 16y, cause unknown					

2 2 5	Balta et al., 2019	F	FA NC A	c.1374delC	Frameshif t	N/A	AT M	c.8977C>T	Missense	P/LP 2*	Homozygou s variant ATM (CHILD OF 188 + 187) 12y presented with mentally retardation and severe malnutrition and clinical signs of Ataxia– telangiectasi a: (ataxia, telangiectasi as of the skin and conjunctiva e, frequent sinopulmon ary infections, bilateral nystagmus, strabismus, hypotonia, muscle weakness, and	Recen t		13	N/A	EXA CT	N

											decreased serum immunoglobulin A level)<br><br>No sign and symptom of secondary cancer including leukaemia and lymphoma as well as no clinical and laboratory data suggestive of FA were present<br><br>Died 13y due to severe sinopulmonary infections					

226	Pearlman et al., 2017	M	APC	c.2377C>T (p.Q793X)	Nonsense	N/A	PMS2	c.1281delT (p.H428Tfs*20)	Frameshift/ Nonsense	N/A	29y Polyposis; MMR proficient tumour Extensive family history of cancers: Fathera(APC) colon 20s, FAP; Mat. aunt cervical 24, multiple myeloma 50; Mat. grandmother colon and lymphoma 66; Mat. grandfather prostate 70s	Recent	29	N/A	EXACT	N
227	Stradella et al., 2019	F	FH	c.905-2A>G p.?	Splice Site	N/A	BARD1	c.157delT; p.(Cys53Valfs*5)	Frameshift/ Nonsense	N/A	Cutaneous leiomyomas 40y Colorectal polyposis 52y Phenotype =	Recent	40	N/A	EXACT	Y

											Reed's syndrome					
228	Stradella et al., 2019	F	ATM	c.3712_3716del<br>p.(Leu1238Lysfs*6)	Frameshift/<br>Nonsense	P/LP2*	FANCA	c.2602-1G>C<br>p.?	Splice Site	N/A	BC 35y<br>Phenotype = HBOC	Recent	35	35	EXACT	N
229	Gong et al., 2012	F	MLH1	c.677G>T<br>R226L	Missense	P3*	MSH6	3959del4	Frameshift/<br>Nonsense	N/A	TRIPLE HET, also BRCA2 mutant Endometrial hyperplasia with atypia, 41y<br>IHC = Loss of MLH1 and PMS2	Recent	41	N/A	EXACT	N
230	Silva-Smith and Sussman, 2018	M	PMS2	c.1882C > T	Nonsense	P1*	BMPR1A	c.25A > T	Nonsense	N/A	Bladder cancer + CRC 39y; IHC=loss of PMS2 staining<br>Relatives as young as 5 with polyps	Recent	39	N/A	EXACT	Y
231	Ouyang et al., 2019	F	PMS2	c.1A>G<br>(p.Met1Val)	Missense	LP3*	ATM	p.E503*	Nonsense	N/A	CRC 67y	Recent	67	N/A	EXACT	N

232	Carbajal-Mamani et al., 2020	F	ATM	Deletion between exons 62 and 63	Deletion<br>Nonsense/Frameshift?	N/A	PALB2	c.2840T>C (p.Leu947Ser)	Missense	VUS2*	46y benign fibroids<br>DCIS 49y; IHC = ER+, PR+<br>OC 50y (clear cell)<br>Family history of breast (x2) and colon (x1) cancer	Recent	46	49	EXACT	Y
233	Fostira et al., 2019	F	ERCC3	c.576_583delCGTGATCC<br>(p.Pro192fs)	Frameshift	N/A	FANCC	c.346-1G>A	Splice Site Acceptor	LP2*	TripNeg BC 34y	Recent	34	34	EXACT	N
234	Fostira et al., 2019	F	PALB2	c.2747_2748+4delAGGTAA	Frameshift	P1*	SLX4	c.4089_4090delAG	Frameshift	N/A	BC 44y<br>OC 48y	Recent	44	44	EXACT	Y
235	Fostira et al., 2019	F	PALB2	c.2257C>T<br>(p.Arg753Ter)	Nonsense	P2*	ATM	c.1562_1563delAG	Frameshift	N/A	BC 33y	Recent	33	33	EXACT	N
236	Stajkovska et al., 2019	M	MLH1	c.333_334delTC p.His112CysfsTer9	Frameshift/<br>Nonsense	N/A	TP53	c.847C > T; p.Arg283Cys	Missense	VUS3*	Glioblastoma 13y	Recent	13	N/A	EXACT	N

237	Genomics England Work Environment	F	BRCA2	13:32340629:CTT>C	Frameshift	N/A	CHEK2	22:28725040:T>A	Stop Gain	N/A	infiltrating duct and lobular carcinoma, 25-30y	1000 Genomes	25-30y	25-30y	AVERAGE	N
238	Genomics England Work Environment	F	BRCA2	13:32340629:CTT>C	Frameshift	N/A	CHEK2	22:28725040:T>A	Stop Gain	N/A	breast adenocarcinoma, 45-50y	1000 Genomes	45-50y	45-50y	AVERAGE	N
239	Genomics England Work Environment	F	ATM	11:108299862:GA>G	Frameshift	N/A	BLM	15:90763016:C>T	Stop Gain	N/A	tubular breast adenocarcinoma, 45-50y	1000 Genomes	45-50y	45-50y	AVERAGE	N
240	Genomics England Work Environment	F	ATM	11:108299862:GA>G	Frameshift	N/A	BLM	15:90763016:C>T	Stop Gain	N/A	breast carcinoma, 45-50y	1000 Genomes	45-50y	45-50y	AVERAGE	N

241	Genomics England<br><br>Work Environment	M	BRCA2	13:32339228:GAA>G	Frameshift	N/A	POLD1	19:50415720:G>C	Unknown	N/A	PGL, 40-45y	1000 Genomes	45-50y	N/A	AVERAGE	N
242	Genomics England<br><br>Work Environment	M	XPA	9:97684982:TC>T	Frameshift	N/A	ERCC5	13:102875551:TGAAAA>T	Frameshift	N/A	pituitary adenoma, 40-45y	1000 Genomes	45-50y	N/A	AVERAGE	N
243	Palmirotta et al., 2018	F	BRCA1	c.1687C>T (p.Gln563Ter)	Nonsense	P3*	BRCA2	c.9976A>T (p.Lys3326Ter)	Nonsense	B3*	BC 40y + 47y; BBC 54y<br><br>Melanoma 54y	Recent	40	40	EXACT	Y
244	Ataei-Kachouei et al., 2015	F	BRCA2	p.Ala938ProfsX21	Nonsense	P3*	STK11	p.S422G	Missense	N/A	MIDC 38y ; IHC = ER+, PR+, HER2?	Recent	38	38	EXACT	N
245	Stradella et al., 2019	F	APC	c.5826_5829del<br><br>p.(Asp1942Glufs*27)	Frameshift/<br><br>Nonsense	P2*	EXO1	c.1900C>T<br><br>p.(Arg634*)	Nonsense	N/A	Colorectal polyposis 52y<br><br>Colorectal cancer 53y	Recent	52	N/A	EXACT	Y

											Phenotype = AFAP					
246	Momozawa et al., 2018	F	BRCA2	p.Gly2529fs	Frameshift	P2*	CHEK2	p.Ala523Thr	Missense	VUS 2*	BC 44y; IHC = ER +, PR+, HER2?	Recent	44	44	EXACT	N
247	Occhi et al., 2010	F	MEN1	E45Q	Missense	N/A	AIP	c.871G>A<br>(V291M)	Missense	N/A	Acromegaly 30y<br>No other MEN1 clinical symptoms	Recent	30	N/A	EXACT	N
248	Yilmaz et al., 2020	M	PMS2	c.187G>A<br>(p. V63M)	Missense	VUS 1*	MSH6	c.3261delC<br>p. F1088fs*2	Frameshift/<br>Nonsense	P3*	CRC 63y<br>Lynch Syndrome	Recent	63	N/A	EXACT	N
249	Nakahara et al., 1997	M	MSH2	c.1916A>G (p.His639Arg)	Missense	VUS 1*	MLH1	c.397G>T<br>(p.Gly133Ter)	Nonsense	P3*	CRC + stomach cancer 41y	Recent	41	N/A	EXACT	Y
250	Nakahara et al., 1997	F	MSH2	c.1916A>G (p.His639Arg)	Missense	VUS 1*	MLH1	c.397G>T<br>(p.Gly133Ter)	Nonsense	P3*	Additional MLH1 variant: A>G Ile (219) Val; Exon 8 Likely Benign (Synonomous) multiple	Recent	57	N/A	EXACT	Y

											CRCs + uterine cancer 57y					
251	Nakahara et al., 1997	F	MSH2	c.1916A>G (p.His639Arg)	Missense	VUS1*	MLH1	c.397G>T<br><br>(p.Gly133Ter)	Nonsense	P3*	Multiple CRCs + uterine cancer 55y	Recent	55	N/A	EXACT	Y
252	Pearlman et al., 2017	F	MSH2	c.80C>T<br><br>(p.P27L)	Missense	VUS2*	MLH1	c.2252_2253delAA<br><br>(p.K751Sfs*3)	Frameshift/<br><br>Nonsense	P3*	CRC 28y<br><br>CRC 46y; MMR deficient	Recent	28	N/A	EXACT	Y
253	Taeubner et al., 2017	?	PTCH1	p.(Gly38Glu)	Missense	LB1*	PTCH2	p.(His622Tyr)	Missense	B1*	congenital embryonal rhabdomyosarcoma<br>no tumour loss of heterozygosity (LOH) in PTCH1 and PTCH2. Tumour had increased GLI1 and SMO expression, suggesting increased	Recent	0	N/A	EXACT	N

											SHH signalling					
254	Kamory et al., 2006	M	MLH1	p.Val716Met	Missense	B3*	MSH2	c.2210+1G>C	Splice Site (Exon 13 deletion out of frame)	LP3*	multiple synchronous colorectal carcinoma 25y IHC = loss of MLH1 and MSH2 expression in both primaries	Recent	25	N/A	EXACT	N
255	Frank-Raue et al., 2005	M	MEN1	IVS5 + 1G>A	Splice Site	LP1*	RET	Y791F	Missense	Conflict	Primary hyperparathyroidism, pancreatic tumor, Fibroma; 35y	Recent	35	N/A	EXACT	Y
256	Frank-Raue et al., 2005	M	MEN1	IVS5 + 1G>A	Splice Site	LP1*	RET	Y791F	Missense	Conflict	Primary hyperparathyroidism, pituitary	Recent	N/A	N/A	EXACT	Y

											tumo, pancreatic tumor, Fibroma; y?; brother of #13					
257	Frank-Raue et al., 2005	F	MEN1	IVS5 + 1G>A	Splice Site	LP1*	RET	Y791F	Missense	Conflict	No clinical features, y?; daughter of #13	Recent	N/A	N/A	EXACT	N
258	Borg et al. 2000	F	BRCA1	c.3047_3048insTGAGA p.(Asn1018Met fs*8)	N/A	N/A	MLH1	c.131C>T p.(Ser44Phe) . Additional nonpathogenic variant c.1321G>A p.(Ala441Thr	N/A	N/A	IDC 35y (MSI low, ERPR -ve)†	Historical	35	35	EXACT	N

259	Pedroni et al. 2013	F	BRCA1	c. 300T>G p.(Cys61Gly)	N/A	N/A	MLH1	c.1489dupC p.(Arg497Pro fs*6)	N/A	N/A	BC 35y; (Loss of MLH1 on IHC . LOH MLH1 and BRCA1)†; Endometrial carcinoma (Loss of MLH1 on IHC. LOH MLH1)‡; Ovarian carcinoma 39y (Loss of MLH1 on IHC. LOH MLH1)*; Renal clear cell carcinoma 39yΔ; Breast cancer (contralateral) 46y (Loss of MLH1 on IHC. LOH and BRCA1)‡	Historical	35	35	EXACT	Y
260	Kast et al. 2012	F	BRCA1	c.213-12A>G, p.(Arg71Ser	N/A	N/A	MSH6	c.515dup p.(Leu173T	N/A	N/A	Endometrial endometroid	Historical	46	N/A	EXACT	N

				fs*21)				hr fs*9)			adenocarcinoma 46y (Loss of MSH6 on IHC)‡					
261	Thiffault et al. 2004	F	BRCA2	c.314T>G p.(Leu105*)	N/A	N/A	MSH2	c.1277_1386 del (Exon 8 deletion)	N/A	N/A	LBC + and DCIS 32y (ERPR +ve)†; Endometrioid adenocarcinoma 40y (No MMR deficiency on IHC. MSI low)Δ; Colon villotubular adenoma. 40 (Loss of MSH2 on IHC. MSI high)‡	Historical	32	32	EXACT	Y
262	Foppiani et al. 2008	M	RET	c.2410G>A p.(Val804Met)	N/A	N/A	CDKN2A	c.142C>A p.(Pro48Thr)	N/A	N/A	Cutaneous malignant melanoma <55y‡; Parathyroid chief cell adenoma 55y†; Thyroid sclerotic	Historical	55	N/A	UNKNOWN	N

											papillary carcinoma 55y†; Thyroid C cell hyperplasia 55y†					
263	Manoukian et al. 2007	F	BRCA2	c.7180A>T p.(Arg2394*)	N/A	N/A	TP53	c.847C>T p.(Arg283Cys)	N/A	N/A	Breast cancer 31y*; Breast cancer 66y*; Leiomyosarcoma of chest wall 71y (In breast radiotherapy field)‡	Historical	31	31	EXACT	Y
264	Monnerat et al. 2007	F	BRCA2	c.4889C>G p.(Ser1630*)	N/A	N/A	TP53	c.329G>T p.(Arg110Leu)	N/A	N/A	Cutaneous malignant melanoma 65y‡; Breast cancer 69y*; Ovarian cancer 69y*;	Historical	65	69	EXACT	Y

											Colon cancer 74y‡					
265	Ghataorhe et al. 2007	F	BRCA2	c.2808_2811del ACAA p.(Ala938Profs *21)	N/A	N/A	MEN1	c.1064+1delG T	N/A	N/A	Abnormal secretory parathyroid gland 34y‡; Pancreastic mass. Unknown histology. Non functional 35y*	Historical	34	34	EXACT	Y
266	Ghataorhe et al. 2007	F	BRCA2	c.2808_2811del ACAA p.(Ala938Profs *21)	N/A	N/A	MEN1	c.1064+1delG T	N/A	N/A	Cushing syndrome (implied pituitary origin) 10y‡; Hypercalcaemia (implied hyperparathyroidism) 31y‡	Historical	10	N/A	EXACT	N
267	Ghataorhe et al. 2007	M	BRCA2	c.2808_2811del ACAA	N/A	N/A	MEN1	c.1064+1delG T	N/A	N/A	Parathyroid hyperpalsia 56y‡; Breast cancer 60y†	Historical	56	60	EXACT	Y

				p.(Ala938Profs *21)												
268	Kashiwada et al. 2012	F	APC	c.637C>T. p.(Arg213*)	N/A	N/A	FLCN	c.1285dup p.(His429Pro fs*27)	N/A	N/A	Facial papules <28y‡; Colon carcinoma and multiple colon polyps 28y†; Recurrent pneumothoraces x4. Pulmonary cysts 28y (first one)‡	Historical	28	N/A	UNKNOWN	Y
269	Kilmartin et al. 1996	M	APC	c.3340 C>T p.(Arg1114*)	N/A	N/A	VHL	Gene deletion (in offspring)	N/A	N/A	Retinal haemangioma x2 21y‡; Cerebellar haemangioblastoma 41y‡ Rectal carcinoma and multiple colonic polyps 41y†	Historical	21	N/A	EXACT	Y

270	Mastroianno et al. 2011	M	RET	c.1997A>T p.(Lys666Met)	N/A	N/A	MEN1	c.669+1G>T	N/A	N/A	Pituitary tumour 38y‡; Primary hyperparathyroidism 45y*; Papillary thyroid cancer 46yΔ; Medullary thyroid cancer 46y†; Gastric carcinoid tumour 47y‡; Gastrinoma ‡	Historical	38	N/A	EXACT	Y
271	Mastroianno et al. 2011	M	RET	c.1997A>T p.(Lys666Met)	N/A	N/A	MEN1	c.669+1G>T	N/A	N/A	Primary hyperparathyroidism 40y*; Cushing syndrome (implied pituitary origin) 40y‡; Carcinoid tumour 40y‡;	Historical	40	N/A	EXACT	Y

											Lipoma 40y‡; Angiofibroma 40y‡; Papillary thyroid cancerΔ; Medullary thyroid cancer 40y†; Gastrinoma 41y‡					
272	Mastroianno et al. 2011	M	RET	c.1997A>T p.(Lys666Met)	N/A	N/A	MEN1	c.669+1G>T	N/A	N/A	No features 6y	Historical	6	N/A	EXACT	N
273	Mastroianno et al. 2011	F	RET	c.1997A>T p.(Lys666Met)	N/A	N/A	MEN1	c.669+1G>T	N/A	N/A	Primary hyperparathyroidism 13y*; Pituitary tumour 15y*	Historical	15	N/A	EXACT	N

274	Plon et al. 2008	F	PTEN	c.334C>G p.(Leu112Val). Cryptic splice site	N/A	N/A	TP53	c.844C>T p.(Arg282Trp)	N/A	N/A	Neuroblastoma 0y‡; Lipoma. Abdominal wall 0y†; Haemangiomas 1y†; Macrocephaly†; Ovarian granulosa cell tumour 1y (No somatic PTEN or TP53 mutations. LOH PTEN . No LOH TP53)Δ; Xanthoastrocytoma, Temporal lobe 3y (No somatic PTEN or TP53 mutations. No LOH PTEN or TP53)Δ; Pelvic	Historical	0	N/A	EXACT	Y

											liposarcoma 4y (No somatic PTEN or TP53 mutations. LOH PTEN . No LOH TP53)Δ					

275	Valle et al. 2004	F	PTEN	c.634+5G>A	N/A	N/A	APC	c.540dup p.(Gln181Thrfs*12)	N/A	N/A	Multiple colonic polyps 10y‡; Subcutaneous nodules‡; Multinodular goitre 26y†; Papillary thyroid cancer, multiple nodular hyperplasia and follicular adenomas 26y†; Diffuse lymphocytic chronic thyroiditis†; Ovarian Morgani hidatide 15yΔ; Cerebellar dysplastic gangliocytoma 26y†; Palmar keratosis 26y†;	Historical	10	N/A	EXACT	Y

											Head fbroma 26y†; Lipomas 26y†; Melanocytic neavi x2 28y†; Facial papules 28y†; Oral papillomatosis 28y†					

276	Zbuk et al. 2007	F	PTEN	c.47dup p.(Tyr16*)	N/A	N/A	SDHC	c.397C>T p.(Arg133*)	N/A	N/A	Macrocepahly†; Papillomatous papules†; Paraganglioma. Left common carotid 18y‡; Fibrocystic breast diasease 20's†; Papillary thyroid cancer 37y†; Paraganglioma. Right carotid body 39y‡; Uterine leiomyomas 30's†	Historical	18	N/A	EXACT	Y

277	Lindor et al. 2012	M	APC	c.694C>T p.(Arg232*)	N/A	N/A	MLH1	c.1732_2271 del (deletion exons 16-19)	N/A	N/A	Rectal carcinoma and multiple colon polyps 14y*; Jejunal adenocarcinoma x6: 28y x3, 34y, 44y, 52y (Loss of MLH1 and PMS2 on IHC)Δ; Duodenal adenocarcinoma 54y*; Congenital hypertrophy of retinal pigment epithelium 54y†; Squamous cell carcinoma. Multiple facialΔ; Pilomatricoma. Scalp 54y†; Sebaceous adenoma 54y (Loss of	Historical	14	N/A	EXACT	Y

											MLH1 and PMS2 on IHC)‡					
278	Soravia et al. 2005	M	APC	c.3471-3474delGAGA p.(Glu1157Aspfs*7)	N/A	N/A	MSH2	c.1192dupG p.(Ala398Glyfs*19)	N/A	N/A	Colon polyps x5. 4 adenomas 24y (1 dysplastic MSI high. Loss of MSH2 and MSH6 on IHC)†; Colon adenocarcinoma. Right colon 25y*; Gastric/duo	Historical	24	N/A	EXACT	Y

											denal adenoma x30 25y*; Desmoid tumour. Mesenteric 26y†					
279	Uhrhammer and Bignon. 2008	M	APC	c.3183_3187delACAAA p.(Gln1062*)	N/A	N/A	MSH2	c.255_256del p.(Phe85Leufs*14)	N/A	N/A	Colon cancer 16y*	Historical	16	N/A	EXACT	N
280	Scheenstra et al. 2003	M	APC	c.3927_3931del p.(1309Aspfs*4)	N/A	N/A	MLH1	c.677G>A p.(Arg226Gln). Affects splicing.	N/A	N/A	Multiple colon polyps (100's) 10†; Tubular adenomas with dysplasia 10y (Loss of MLH1 on IHC)*	Historical	10	N/A	EXACT	Y
281	Puijenbroek et	F	MSH6	c.1784delT p.(Leu595fs*15)	N/A	N/A	MUTYH	c.536A>G p.(Tyr179Cys) and	N/A	N/A	Colon adenomas x5 48y (All MSI stable.	Historical	48	N/A	EXACT	Y

	al. 2007							c.1187G>A<br>p.(Gly396Asp)			Retained MSH6 expression) ‡					
282	Pern et al. 2012	F	BRCA1	c.927delA<br>p.(Lys309Asnfs*5)	N/A	N/A	PALB2	c.756dup<br>p.(Leu253Serfs*4)	N/A	N/A	Uterine myomas <65yΔ; Meningioma <65yΔ; Breast invasive ductal carcinoma. Multifocal 65y (Triple negative histology)*	Historical	65	65	UNKNOWN	Y
283	Ercolino et al.<br>2014	M	NF1	c.1185+1G>A	N/A	N/A	RET	c.2410G>A<br>p.(Val804Met)	N/A	N/A	Macrocephaly, café au lait patches and axillary freckling 57y†; Kyphoscoliosis 57y†; Multiple cutaneous neurofibromas 57y†; Thyroid C-cell	Historical	57	N/A	EXACT	N

											hyperplasia 57y‡; Parathyroid hyperplasia 57y‡					
284	Campos et al. 2013	F	BRCA1	c.4107_4110dup p.(Gly1371Ilefs*4)	N/A	N/A	NF1	c.4120C>T p.(Gln1374*)	N/A	N/A	Café au lait patches, multiple cutaneous neurofibromas and Axillary/inguinal freckling in childhood‡; Breast infiltrating duct carcinoma 35y‡	Historical	35	35	EXACT	Y
285	Augustyn et al. 2011	F	BRCA1	c.1961delA p.(Lys654Serfs*47)	N/A	N/A	BRCA2	c.1672delC p,(Ile558Leufs*15)	N/A	N/A	Ovarian serous carcinoma with papillary features.	Historical	50	N/A	EXACT	Y

											Bilateral 50y*					
286	Augustyn et al. 2011	F	BRCA1	c.5266dupC p.(Gln1756Profs*74)	N/A	N/A	BRCA2	c.4829_4830del p.(Val1610Glyfs*4)	N/A	N/A	Breast cancer 40y (Triple negative histology)*	Historical	40	40	EXACT	N
287	Bell et al. 2002	F	BRCA1	c.5266dupC p.(Gln1756Profs*74)	N/A	N/A	BRCA2	c.5946delT p.(Ser1982Argfs*22)	N/A	N/A	Breast cancer 33y (LOH BRCA2. No LOH BRCA1)‡; Breast cancer 44y (LOH BRCA2. No LOH BRCA1)‡; Breast cancer 47y (LOH BRCA1. No LOH BRCA2)†	Historical	33	33	EXACT	Y
288	Caldes 2002	F	BRCA1	c.5123C>A p.(Ala1708Glu)	N/A	N/A	BRCA2	c.6275_6276del p.(Leu2092Profs*7)	N/A	N/A	No features	Historical	N/A	N/A	EXACT	N

289	Caldes 2002	M	BRCA1	c.5123C>A p.(Ala1708Glu)	N/A	N/A	BRCA2	c.6275_6276del p.(Leu2092Profs*7)	N/A	N/A	Prostate cancer 66y*	Historical	66	N/A	EXACT	N
290	Caldes 2002	F	BRCA1	c.5123C>A p.(Ala1708Glu)	N/A	N/A	BRCA2	c.6275_6276del p.(Leu2092Profs*7)	N/A	N/A	Breast cancer 70y*	Historical	70	70	EXACT	N
291	Caldes 2002	F	BRCA1	c.5123C>A p.(Ala1708Glu)	N/A	N/A	BRCA2	c.6275_6276del p.(Leu2092Profs*7)	N/A	N/A	Breast cancer 66y*	Historical	66	66	EXACT	N
292	Caldes 2002	F	BRCA1	c.5123C>A p.(Ala1708Glu)	N/A	N/A	BRCA2	c.6275_6276del p.(Leu2092Profs*7)	N/A	N/A	Breast cancer 28y (No LOH BRCA1 or BRCA2)*	Historical	28	28	EXACT	N
293	Caldes 2002	F	BRCA1	c.5123C>A p.(Ala1708Glu)	N/A	N/A	BRCA2	c.6275_6276del p.(Leu2092P	N/A	N/A	No features	Historical	N/A	N/A	EXACT	N

								rofs*7)							
294	Caldes 2002	F	BRCA1	c.5123C>A p.(Ala1708Glu)	N/A	N/A	BRCA2	c.6275_6276del p.(Leu2092Profs*7)	N/A	N/A	No features	Historical	N/A	N/A	EXACT	N
295	Caldes 2002	F	BRCA1	c.5123C>A p.(Ala1708Glu)	N/A	N/A	BRCA2	c.6275_6276del p.(Leu2092Profs*7)	N/A	N/A	No features	Historical	N/A	N/A	EXACT	N
296	Choi et al. 2006	F	BRCA1	c.1504_1508delTTAAA p.(Leu502Alafs*2)	N/A	N/A	BRCA2	c.2798_2799delCA p.(Thr933Argfs*2)	N/A	N/A	Breast infiltrating duct carcinoma 26y*	Historical	26	26	EXACT	N
297	Choi et al. 2006	F	BRCA1	c.4981G>T p.(Glu1661*)	N/A	N/A	BRCA2	c.5946delT p.(Ser1982Argfs*22)	N/A	N/A	Breast infiltrating duct carcinoma 33y*	Historical	33	33	EXACT	N

298	Heidemann et al. 2012	F	BRCA1	c.5266dup p.(Gln1756Profs*74)	N/A	N/A	BRCA2	c.5645C>G p.(Ser1882*)	N/A	N/A	Breast cancer 37y*; Breast cancer 39y*; Ovarian cancer 63y*	Historical	37	37	EXACT	Y
299	Heidemann et al. 2012	F	BRCA1	c.68_69delAG p.(Glu23Valfs*17)	N/A	N/A	BRCA2	c.5718_5719del p.(Leu1908Argfs*2)	N/A	N/A	No features	Historical	N/A	N/A	EXACT	N
300	Heidemann et al. 2012	F	BRCA1	c.68_69delAG p.(Glu23Valfs*17)	N/A	N/A	BRCA2	c.5718_5719del p.(Leu1908Argfs*2)	N/A	N/A	Breast cancer 32y*	Historical	32	32	EXACT	N
301	Heidemann et al. 2012	F	BRCA1	c.962G>A p.(Trp321*)	N/A	N/A	BRCA2	c. 2231C>G p.(Ser744*)	N/A	N/A	Breast cancer 31y*; Breast cancer (contralateral) 35y*	Historical	31	31	EXACT	Y

302	Heidemann et al. 2012	F	BRCA1	c.3910delG p.(Glu1304Lysfs*3)	N/A	N/A	BRCA2	c.2830A>T p.(Lys944*)	N/A	N/A	Breast cancer 39y*	Historical	39	39	EXACT	N
303	Heidemann et al. 2012	F	BRCA1	c.5277+1delG	N/A	N/A	BRCA2	c.658_659del GT p.(Val220Ilefs*4)	N/A	N/A	Colorectal cancer. Caecal 58yΔ; Ovarian cancer 61y*	Historical	58	N/A	EXACT	Y
304	Heidemann et al. 2012	F	BRCA1	c.5277+1delG	N/A	N/A	BRCA2	c.658_659del GT p.(Val220Ilefs*4)	N/A	N/A	No features	Historical	N/A	N/A	EXACT	N
305	Heidemann et al. 2012	F	BRCA1	c.3700_3704del GTAAA p.(Val1234Glnfs*8)	N/A	N/A	BRCA2	c.1813_1814insA p.(Ile605Asnfs*11)	N/A	N/A	Cervical cancer 26yΔ; Breast cancer 40y*	Historical	26	40	EXACT	Y

306	Leegte et al. 2005	F	BRCA1	c.2685_2686delAA p.(Pro897fs*5)	N/A	N/A	BRCA2	c.3487delG p. (Asp1163Ilefs*5)	N/A	N/A	Ovarian papillary serous cystadenocarcinoma 40y (LOH BRCA2)*; Breast infiltrative ductal carcinoma 45y (LOH BRCA1)*	Historical	40	45	EXACT	Y
307	Leegte et al. 2005	F	BRCA1	c.2685_2686delAA p.(Pro897fs*5)	N/A	N/A	BRCA2	c.4449delA p.(Asp1484Thrfs*2)	N/A	N/A	Breast cancer. Ductal 28y*	Historical	28	28	EXACT	N
308	Leegte et al. 2005	F	BRCA1	c.66_67delAG p.(Glu23Valfs*17)	N/A	N/A	BRCA2	c.5946delT p.(Ser1982Argfs*22)	N/A	N/A	No features	Historical	N/A	N/A	EXACT	N
309	Leegte et al. 2005	F	BRCA1	c.5263_5264insC	N/A	N/A	BRCA2	c.5946delT p.(Ser1982Ar	N/A	N/A	Breast invasive lobular carcinoma 51y*	Historical	51	51	EXACT	N

				p.(Ser1756Profs*74)				gfs*22)								
310	Liede et al. 1998	F	BRCA1	c.2389G>T p.(Glu797*)	N/A	N/A	BRCA2	c.3067_3068insA p.(Asn1023Lysfs*3)	N/A	N/A	Breast adenocarcinoma 35y*	Historical	35	35	EXACT	N
311	Loubser et al. 2012	M	BRCA1	c.2641G>T p.(Glu881*)	N/A	N/A	BRCA2	c.7934delG p.(Arg2645Asnfs*3)	N/A	N/A	No features 49y	Historical	49	N/A	EXACT	N
312	Loubser et al. 2012	F	BRCA1	c.2641G>T p.(Glu881*)	N/A	N/A	BRCA2	c.7934delG p.(Arg2645Asnfs*3)	N/A	N/A	Breast ductal carcinoma 42y	Historical	42	N/A	EXACT	N
313	Moslehi et al. 2000	F	BRCA1	c.66_67delAG p.(Glu23Valfs*17)	N/A	N/A	BRCA2	c.5946delT p.(Ser1982Argfs*22)	N/A	N/A	No features 36y	Historical	36	N/A	EXACT	N

314	Musolino et al. 2005	F	BRCA1	c.4285_4286insG p.(Tyr1429*)	N/A	N/A	BRCA2	c.7738C>T p.(Gln2580*)	N/A	N/A	Breast infiltrating duct carcinoma 37y (Triple negative histology)*	Historical	37	37	EXACT	N
315	Noh et al. 2011	F	BRCA1	c.3746_3747insA p.(Glu1250Argfs*5)	N/A	N/A	BRCA2	c.6952_6953del p.(Arg2318Lysfs*21)	N/A	N/A	Breast infiltrating duct carcinoma 26y*	Historical	26	26	EXACT	N
316	Noh et al. 2011	F	BRCA1	c.390C>A p.(Tyr130*)	N/A	N/A	BRCA2	c.3018delA p.(Gly1007Valfs*36)	N/A	N/A	Breast infiltrating duct carcinoma 45y*	Historical	45	45	EXACT	N
317	Noh et al. 2011	F	BRCA1	c.5030_5033delCTAA p.(Thr1677Ilefs*2)	N/A	N/A	BRCA2	c.1399A>T p.(Lys467*)	N/A	N/A	Breast infiltrating duct carcinoma 35y*	Historical	35	35	EXACT	N

318	Pilato et al. 2010	F	BRCA1	c.5263_5264du p p.(Gln1756Prof s*10)	N/A	N/A	BRCA2	c.5796_5797 delTA p.(His1932Gl nfs*12)	N/A	N/A	Breast intraductal carcinoma 38y (Triple negative histology)*; Ovarian papillary adenocarcinoma. Bilateral 42y*	Historical	38	38	EXACT	Y
319	Smith et al. 2008	F	BRCA1	c.3331_3334de lCAAG p.(Gln1111Asnf s*5)	N/A	N/A	BRCA2	c.631+2T>G	N/A	N/A	Breast cancer 34y*; Colorectal carcinoma. Transverse. (No loss of MMR proteins on IHC. No microsatellite instability) 35yΔ; Breast cancer 53y*	Historical	34	34	EXACT	Y
320	Smith et al. 2008	F	BRCA1	c.3331_3334de lCAAG p.(Gln1111	N/A	N/A	BRCA2	c.631+2T>G	N/A	N/A	No features 65y	Historical	65	N/A	EXACT	N

				Asnfs*5)												
321	Tesoriero et al. 1999	F	BRCA1	c.3769_3770delGA p.(Glu1257Glyfs*9)	N/A	N/A	BRCA2	c.5946delT p.(Ser1982Argfs*22)	N/A	N/A	Breast cancer <40y (LOH BRCA2)*	Historical	N/A	N/A	UNKNOWN	N
322	Zuradelli et al. 2010	F	BRCA1	c.835delC p.(His279Metfs*19)	N/A	N/A	BRCA2	c.8195T>G p.(Leu2732*)	N/A	N/A	Breast carcinoma. Metaplastic 43y (Triple negative histology)*	Historical	43	43	EXACT	N
323	Zuradelli et al. 2010	F	BRCA1	c.3916_3917delTT p.(Leu1306Aspfs*23)	N/A	N/A	BRCA2	c.5380delG p.(Val1794*)	N/A	N/A	Breast ductal cancer. Medullary type 30y (ERPRve)*; Ovarian serous papillary carcinoma 36y*	Historical	30	30	EXACT	Y

324	Zuradelli et al. 2010	F	BRCA1	c.1687C>T p.(Gln563*)	N/A	N/A	BRCA2	c.6469C>T p.(Gln2157*)	N/A	N/A	Breast infiltrating duct carcinoma 2x foci 46y (1 lymph node ERPR -ve. 1 lymph node ERPR+ve)*	Historical	46	46	EXACT	N
325	Zuradelli et al. 2010	F	BRCA1	c.2405_2406delTG p.(Val802Glufs*7)	N/A	N/A	BRCA2	c.4285C>T p.(Gln1429*)	N/A	N/A	Breast ductal carcinoma 52y(Triple negative histology)* Ovarian serous adenocarcinoma. Bilateral 52y*	Historical	52	52	EXACT	Y
326	Friedman et al. 1998	F	BRCA1	c.66_67delAG p.(Glu23Valfs*17)	N/A	N/A	BRCA2	c.5946delT p.(Ser1982Argfs*22)	N/A	N/A	Breast cancer 38y*	Historical	38	38	EXACT	N
327	Friedman et al.	F	BRCA1	c.66_67delAG p.(Glu23Val	N/A	N/A	BRCA2	c.5946delT p.(Ser1982Ar	N/A	N/A	Ovarian cancer 57y*	Historical	57	N/A	EXACT	N

	1998			fs* 17)				gfs*22)								
328	Friedman et al. 1998	F	BRCA1	c.66_67delAG p.(Glu23Valfs* 17)	N/A	N/A	BRCA2	c.5946delT p.(Ser1982Ar gfs*22)	N/A	N/A	No features	Historical	N/A	N/A	EXACT	N
329	Friedman et al. 1998	F	BRCA1	c.66_67delAG p.(Glu23Valfs* 17)	N/A	N/A	BRCA2	c.5946delT p.(Ser1982Ar gfs*22)	N/A	N/A	Breast cancer 45y*	Historical	45	45	EXACT	N
330	Ramus et al. 1997	F	BRCA1	c.66_67delAG p.(Glu23Valfs* 17)	N/A	N/A	BRCA2	c.5946delT p.(Ser1982Ar gfs*22)	N/A	N/A	Breast cancer 48y*; Ovarian cancer 50y*	Historical	48	48	EXACT	Y
331	Leegte et al. 2005 Frank et al. 2002	Unknown	BRCA1	c.66_67delAG p.(Glu23Valfs* 17)	N/A	N/A	BRCA2	c.5946delT p.(Ser1982Ar gfs*22)	N/A	N/A	Breast cancer 39y*	Historical	39	39	EXACT	N

332	Leegte et al. 2005 Frank et al. 2002	Unknown	BRCA1	c.66_67delAG p.(Glu23Valfs*17)	N/A	N/A	BRCA2	c.5946delT p.(Ser1982Argfs*22)	N/A	N/A	Breast cancer 41y*	Historical	41	41	EXACT	N
333	Leegte et al. 2005 Frank et al. 2002	Unknown	BRCA1	c.66_67delAG p.(Glu23Valfs*17)	N/A	N/A	BRCA2	c.5946delT p.(Ser1982Argfs*22)	N/A	N/A	Breast cancer. Bilateral 34y*	Historical	34	34	EXACT	Y
334	Leegte et al. 2005 Frank et al. 2002	Unknown	BRCA1	c.66_67delAG p.(Glu23Valfs*17)	N/A	N/A	BRCA2	c.5946delT p.(Ser1982Argfs*22)	N/A	N/A	Breast cancer 55y*; Breast cancer (contralateral) 56y*	Historical	55	55	EXACT	Y
335	Leegte et al. 2005 Frank et al. 2002	Unknown	BRCA1	c.66_67delAG p.(Glu23Valfs*17)	N/A	N/A	BRCA2	c.5946delT p.(Ser1982Argfs*22)	N/A	N/A	No features	Historical	N/A	N/A	EXACT	N
336	Leegte et al. 2005 Frank	Unknown	BRCA1	c.66_67delAG p.(Glu23Valfs*	N/A	N/A	BRCA2	c.5946delT p.(Ser1982Ar	N/A	N/A	Breast cancer 40y*	Historical	40	40	EXACT	N

	et al. 2002			17)				gfs*22)								
337	Leegte et al. 2005<br><br>Frank et al. 2002	Unknown	BRCA1	c.66_67delAG<br><br>p.(Glu23Valfs*17)	N/A	N/A	BRCA2	c.5946delT<br><br>p.(Ser1982Argfs*22)	N/A	N/A	Breast cancer 33y*; Breast cancer (contralateral) 49y*	Historical	33	33	EXACT	Y
338	Leegte et al. 2005<br><br>Frank et al. 2002	Unknown	BRCA1	c.66_67delAG<br><br>p.(Glu23Valfs*17)	N/A	N/A	BRCA2	c.5946delT<br><br>p.(Ser1982Argfs*22)	N/A	N/A	No features 61y	Historical	61	N/A	EXACT	N
339	Randall et al.<br><br>1998	F	BRCA1	c.66_67delAG<br><br>p.(Glu23Valfs*17)	N/A	N/A	BRCA2	c.5946delT<br><br>p.(Ser1982Argfs*22)	N/A	N/A	Breast cancer. Multifocal lobular carcinoma 30y (LOH BRCA1)*; Ovarian cancer 41y (LOH BRCA1 and BRCA2)*	Historical	30	30	EXACT	Y

340	Whitworth et al. 2016	M	FLCN	c.1062+2T>G	N/A	N/A	NF1	c.1381C>T p.(Arg461*)	N/A	N/A	Testicular seminoma 39yΔ; Renal cell carcinoma. Chromophobe 55y†; Phaeochromocytoma 43y‡; Gastrointestinal stromal tumour x2 55y‡; Malignant peripheral nerve sheath tumour 56y‡; Multiple cutaneous neurofbromas‡; Cafe au lait patches‡; Recurrent pneumothoraces.	Historical	39	N/A	EXACT	Y

341	Whitworth et al. 2016	M	FLCN	c.715C>T p.(Arg239Cys)	N/A	N/A	TP53	c.526T>C p.(Cys176Arg)	N/A	N/A	Rectal carcinoma 27yΔ; Gastroesophageal adenocarcinoma 32yΔ; Renal cell carcinoma. Chromophobe 32y†; Facial fbrofolliculomas†.	Historical	27	N/A	EXACT	Y
342	Whitworth et al. 2016	F	FLCN	c.1285delC p.(His429Thrfs*39)	N/A	N/A	MSH2	c.892C>T p.(Gln298*)	N/A	N/A	Pneumothorax 37y†; Endometrial cancer 52y‡.	Historical	52	N/A	EXACT	N
343	Whitworth et al. 2016	F	FLCN	c.1285delC p.(His429Thrfs*39)	N/A	N/A	MSH2	c.892C>T p.(Gln298*)	N/A	N/A	Renal cell carcinoma†; Colorectal polyps‡; Multiple pneumothoraces†.	Historical	N/A	N/A	EXACT	Y
344	Whitworth et al. 2016	M	FLCN	c.1285delC p.(His429Thrfs*39)	N/A	N/A	MSH2	c.892C>T p.(Gln298*)	N/A	N/A	Facial fibrofolliculomas†	Historical	N/A	N/A	EXACT	N

345	Whitworth et al. 2016	M	MLH1	c.306G>T p.(Glu102Asp)	N/A	N/A	XPA	c.555+8A>G	N/A	N/A	Caecal cancer. Mucinous 65y†; Sigmoid cancer 67y†; Previous skin tumours including squamous carcinoma in an actinic keratosis, multiple seborrhoeic keratoses, keratoacanthomata/ squamous carcinomas x2, junctional naevi, squamous carcinoma and lentigo malignae x2‡	Historical	65	N/A	EXACT	Y

346	Whitworth et al. 2016	F	BRCA2	c.5213_5216del p.(Thr1738Ilefs*2)	N/A	N/A	NF1	c.6792C>G p.(Tyr2264*)	N/A	N/A	Breast ductal carcinoma 48y*; Cutaneous melanoma 57y†; Multiple cutaneous neurofibromas‡; Malignant peripheral nerve sheath tumour‡; Café au lait patch‡; Possible Lisch nodules‡.	Historical	48	48	EXACT	Y
347	Lorca et al., 2019	F	POLE	c.3857G > A p.(Arg1286His)	Nonsense	VUS2*	NTHL1	c.527T > C p.(Ile176Thr)	Nonsense	LB2*	Adenomatous Polyposis x24 70y endometrial hyperplasia 70y hypothyroidism 70y	Recent	70	N/A	EXACT	Y
348	Wen et al., 2019	M	BRCA1	S1841Vfs*2 (c.5521delA)	Frameshift	P3*	BRCA2	Q1886* (c.5656C>T)	Nonsense	P3*	Gastric cancer 50y	Recent	50	N/A	EXACT	N

349	Stolavora et al., 2020	F	POT1	c.347C>T (p.P116L)	Missense	VUS1*	CHEK2	c.909-2028_1095+330del5395 (p.M304Lfs*15)	Frameshift	N/A	Melanoma 41y Melanoma 42y Melanoma 44y BC 47y	Recent	41	47	EXACT	Y
350	Stolavora et al., 2020	F	OCA2	c.1211C>T (p.T404M)	Missense	LP1*	KAT6A	c.1138G>T (p.E380*)	Nonsense	N/A	29y	Recent	29	N/A	EXACT	N
351	Stolavora et al., 2020	M	TYRP1	c.1054_1057del4 (p.N353Vfs*31)	Frameshift	N/A	TRPM1	Δe2-7 (p.?)		N/A	Melanoma 36y	Recent	36	N/A	EXACT	N
352	Stolavora et al., 2020	M	SLC45A2	Δe1-2 (p.?)	Frameshift	N/A	GSTM3	c.393C>A (p.Y131*)	Nonsense	N/A	Melanoma 25y	Recent	25	N/A	EXACT	N
353	Stolavora et al., 2020	F	TYR	c.1037-7T>A (p.?)		P/LP2* - Albinism	FANCC	c.455dupA (p.N152Kfs*9)	Frameshift	P1*	BC 52y Melanoma 66y CRC 66y	Recent	52	52	EXACT	Y
354	Stolavora et al., 2020	F	NBN	c.1723G>T (p.E575*)	Nonsense	P/LP2*	NFKBIE	c.165_169dup5 (p.E57Afs*51)	Frameshift/Nonsense	VUS	Melanoma 9y	Recent	9	N/A	EXACT	N
355	Stolavora et al., 2020	M	BRCA2	c.7007G>A (p.R2336H)	Missense	P3*	IFIH1	c.2464C>T (p.R822*)	Nonsense	VUS	Melanoma 22y	Recent	22	N/A	EXACT	N

356	Stolavora et al., 2020	M	BRCA2	c.8168_8172ins4 (p.Y2726Mfs*10)	Frameshift	P3*	TYRP1	c.1254C>A (p.Y418*)	Nonsense	VUS	Melanoma 36y Non-Hodgkin Lymphoma 38y	Recent	36	N/A	EXACT	Y
357	Stolavora et al., 2020	F	BRCA1	c.4214delT (p.I1405Kfs*10)	Frameshift	N/A	ATM	c.7630-2A>C (p.?)	Splice site	P2*	Triple Het - *MUTYH* c.1187G>A (p.G396D) Melanoma 46y OC 46y BC 49y	Recent	46	49	EXACT	Y
358	Stolavora et al., 2020	F	ATM	c.381delA (p.V128*)	Nonsense	P2*	WRN	c.1105C>T (p.R369*)	Nonsense	P2*	Melanoma 41y Melanoma 50y	Recent	41	N/A	EXACT	Y
359	Stolavora et al., 2020	F	RAD51D	c.405+2T>C (p.?)	Splice site	Conflicting LP/VUS1*	CHEK2	c.917G>C (p.G306A)	Missense	Conflicting LP/VUS1*	Melanoma 26y	Recent	26	N/A	EXACT	N
360	Zheng et al., 2020	F	BRCA1	c.3348_3351delAGTT p.V1117Rfs*11	Frameshift	N/A	MSH2	large deletion of exons 4-16	Frameshift	N/A	Endometrial carcinoma 52y	Recent	52	N/A	EXACT	N
361	Pazderová et al., 2020		BRCA2	c.8487G>T p.Gln2829His	Splice Site	N/A	CHEK2	Deletion of exons 9-10	Frameshift	N/A	Pancreatic Cancer 50y	Recent	50	N/A	EXACT	N

362	Sukumar et al., 2021	F	BRCA1	c.181T > G	Missense	P3*	BRCA2	c.4398_4402delACATT	Frameshift	P2*	Triple Het - CHEK2 1100delC BilatBC 55y; IHC(1) = ER-,PR+,HER2-, IHC(2) =ER+,PR+,HER2-	Recent	55	55	EXACT	Y
363	Sukumar et al., 2021	F	BRCA1	c.181T > G	Missense	P3*	CHEK2	c.1100delC	Frameshift	1*Conflict	BC 34y; IHC = ER+,PR-,HER2-	Recent	34	34	EXACT	N
364	Sukumar et al., 2021	F	BRCA1	c.181T > G	Missense	P3*	CHEK2	c.1100delC	Frameshift	1*Conflict	29y	Recent	29	N/A	EXACT	N
365	Agiannitopoulos et al., 2020	F	MSH2	Deletion of exons 11-16		N/A	PALB2	c.757_758delCT p.(Leu253Ilefs*3)	Frameshift	N/A	Endometrial Cancer 42y	Recent	42	N/A	EXACT	N
366	Le Duc et al., 2020	M	TP53	c.394A>G	Missense	Conflicting LP/P/VUS1*	RAD51C	Deletion of exons 5-9		N/A	38y Basal Cell Carcinoma 41y Sabaceous Gland Carcinoma	Recent	38	N/A	EXACT	Y
367	Vietri et al., 2020	F	BRCA1	c.3756_3759delGTCT (p.Ser1253Argfs)	Frameshift	P2*	APC	c.3927_3931delAAAGA (p.Glu1309AspfsX4)	Frameshift	N/A	Profuse FAP 18y Desmoid	Recent	18	N/A	EXACT	Y

											Tumour 22y OC 45y					
368	Del Valle et al., 2020	F	FANCA	c.2602-1G>C p.?	Splice Site	N/A	ATM	Unknown	Unknown	N/A	BC 35y	Recent	35	35	EXACT	N
369	Del Valle et al., 2020	F	FANCA	c.3588dup p.(Arg1187Glufs*28)	Frameshift	P1*	SDHB	Unknown	Unknown	N/A	OC 49y	Recent	49	N/A	EXACT	N
370	Del Valle et al., 2020	M	FANCL	c.1111_1114dup p.(Thr372Asnfs*13)	Frameshift	Conflicting VUS/B1*	MLH1	Unknown	Unknown	N/A	CRC 29y	Recent	29	N/A	EXACT	N
371	Ramus et al., 1997; Le Page 2020	F	BRCA1	c.3916-3917del	Frameshift	P3*	BRCA2	c.5380delG	Frameshift	P3*	BC 30y OC 36y	Recent	30	30	EXACT	Y
372	Ramus et al., 1997; Le Page 2020	F	BRCA1	c.1687C>T	Missense	P3*	BRCA2	c.6469C>T	Missense	P3*	BC 46y OC 58y	Recent	46	46	EXACT	Y
373	Ramus et al., 1997; Le	F	BRCA1	c.2405_2406del	Frameshift	P2*	BRCA2	c.4285C>T	Missense	P3*	BC 52y OC 52y	Recent	52	52	EXACT	Y

	Page 2020															
374	Randall et al., 1998;Le Page 2020	F	BRCA1	c.68_69delAG	Frameshift	P3*	BRCA2	c.5946delT	Frameshift	P3*	OC 57y	Recent	57	N/A	EXACT	N
375	Randall et al., 1998;Le Page 2020	F	BRCA1	c.68_69delAG	Frameshift	P3*	BRCA2	c.5946delT	Frameshift	P3*	OC 50y	Recent	50	N/A	EXACT	N
376	Liede et al., 1998; Le Page 2020	F	BRCA1	c.68_69delAG	Frameshift	P3*	BRCA2	c.5946delT	Frameshift	P3*	BC 48y OC 50y	Recent	48	48	EXACT	Y
377	Suspitsin et al., 2013	F	BRCA1	5382insC	Frameshift	P3*	BLM	Q548X	Nonsense	P2*	BC 68y IHC = TripNeg	Recent	68	68	EXACT	N
378	Suspitsin et al., 2013	F	BRCA1	5382insC	Frameshift	P3*	BLM	Q548X	Nonsense	P2*	BC 68y IHC = TripNeg	Recent	68	68	EXACT	N
379	Suspitsin et al., 2013	F	CHEK2	c.1100delC	Frameshift	1*Conflict	BLM	Q548X	Nonsense	P2*	BC 51y IHC = ER+,PR+,HER2-	Recent	51	51	EXACT	N
380	Suspitsin et	F	CHEK2	5395del	Frameshift	N/A	BRCA1	5382insC	Frameshift	P3*	BC 52y IHC = ER+,PR+,HER2-	Recent	52	52	EXACT	N

	al., 2013															
381	Suspitsin et al., 2013	F	NBN	657del5	Frameshift	P2*	BLM	Q548X	Nonsense	P2*	BC 48y IHC = ER-,PR+,HER2+	Recent	48	48	EXACT	N
382	Penkert et al., 2018	F	ATM	p.(Cys2931*)	Nonsense	P/LP2*	CHEK2	exon 9–10 del	Frameshift	N/A	BC 39y	Recent	39	39	EXACT	N
383	Penkert et al., 2018	F	CDKN2A	p.(Arg98*)	Nonsense	Conflicting 1* LP/VUS	RECQL4	c.1390+1G>C	Splice Site	N/A	BC 32y IHC = HER2+	Recent	32	32	EXACT	N
384	Penkert et al., 2018	F	FANCI	p.(Arg1285*)	Nonsense	N/A	PMS2	exon 3–8 del	Frameshift	N/A	BC 30y IHC = HER2+	Recent	30	30	EXACT	N
385	Ohmoto et al., 2018	M	PMS2	c.631 C> T p.Arg211Ter	Nonsense	P1*	CHEK2	c.1111 C>T p.His371Tyr	Missense	VUS 2*	CRC 58y	Recent	58	N/A	EXACT	N

Supplementary Table 5: Details of MINAS cases with multiple primary tumours. Those in which atypical tumour types were present are highlighted in bold

Case	Reference	Sex	Gene 1	Gene 2	Primary Tumours	Subgroup
2	Le Page et al., 2018	F	BRCA1	BRCA2	Breast, Ovarian	Recent
18	Rebbeck et al., 2016	F	BRCA1	BRCA2	Breast, Ovarian	Recent
19	Rebbeck et al., 2016	F	BRCA1	BRCA2	Breast, Ovarian	Recent
20	Rebbeck et al., 2016	F	BRCA1	BRCA2	Breast, Ovarian	Recent
38	Rebbeck et al., 2016	F	BRCA1	BRCA2	Breast, Ovarian	Recent
39	Rebbeck et al., 2016	F	BRCA1	BRCA2	Breast, Ovarian	Recent
46	Rebbeck et al., 2016	F	BRCA1	BRCA2	Breast, Ovarian	Recent
60	Rebbeck et al., 2016	F	BRCA1	BRCA2	Breast, Ovarian	Recent
62	Rebbeck et al., 2016	F	BRCA1	BRCA2	Breast, Ovarian	Recent
78	Rebbeck et al., 2016	F	BRCA1	BRCA2	Breast, Ovarian	Recent
81	Rebbeck et al., 2016	F	BRCA1	BRCA2	Breast, Ovarian	Recent
85	Rebbeck et al., 2016	F	BRCA1	BRCA2	Breast, Ovarian	Recent
86	Rebbeck et al., 2016	F	BRCA1	BRCA2	Breast, Ovarian	Recent
88	Spannuth et al., 2007	F	BRCA1	BRCA2	Breast, Breast, Ovarian	Recent
89	Meynard et al., 2018	F	BRCA1	BRCA2	Breast, Breast	Recent
91	Vietri et al., 2013	F	BRCA1	BRCA2	Breast, Breast	Recent
126	Steffensen et al., 2010	F	BRCA1	BRCA2	Breast, Ovarian	Recent
**131**	Nomizu et al., 2015	F	BRCA1	BRCA2	Breast, **Endometrial**	Recent
137	Nurmi et al., 2019	F	BRCA1	CHEK2	Breast, Breast	Recent
138	Nurmi et al., 2019	F	BRCA1	FANCM	Breast, Ovarian	Recent
141	Nurmi et al., 2019	F	BRCA2	PALB2	Breast, Breast	Recent
144	Nurmi et al., 2019	F	BRCA2	FANCM	Breast, Breast	Recent
145	Nurmi et al., 2019	F	BRCA2	FANCM	Breast, Breast	Recent
147	Sokolenko et al., 2014	F	BRCA1	BLM	Breast, Ovarian	Recent

**148**	Sokolenko et al., 2014	F	BRCA1	BLM	Breast, **Non-Hodgkin's Lymphoma**	Recent
**155**	Andrés et al., 2019	F	BRCA1	ATM	Breast, **Neuroendocrine**, Endometrial	Recent
159	Tedaldi et al., 2017	F	BRCA2	BRIP1	Breast, Breast	Recent
160	Tedaldi et al., 2017	F	BRCA2	ATM	Breast, Breast	Recent
165	Fostira et al., 2019	F	BRCA2	RAD51C	Breast, Breast	Recent
166	Fostira et al., 2019	F	BRCA2	SDHC	Breast, Breast	Recent
171	Nurmi et al., 2019	F	CHEK2	ATM	Breast, Bladder	Recent
**176**	Stradella et al., 2019	F	MLH1	MEN1	Pituitary, Parathyroid, Neuroendocrine, **Haemangioma**	Recent
**177**	Stradella et al., 2019	F	MLH1	MEN1	Uterine, CRC, **Haemangioma**	Recent
179	Stradella et al., 2019	F	PALB2	ATM	Breast, Pancreas	Recent
181	Njoroge et al., 2017	F	CDH1	PMS2	Breast, Thyroid	Recent
184	Vahteristo et al., 2001	F	MSH6	CHEK2	Breast, CRC	Recent
187	Sokolenko et al., 2014	F	CHEK2	NBN	Breast, Breast	Recent
201	Fostira et al., 2019	F	BRCA1	BRCA2	Breast, Breast	Recent
208	Bell et al., 2014	F	BRCA1	TP53	Breast, Breast	Recent
214	Papi et al., 2009	F	BRCA1	MEN1	Pancreatic Neuroendocrine, Pancreatic Neuroendocrine, Lipoma	Recent
215	Sekido et al., 2017	F	BRCA2	KIT	Breast, GIST	Recent
218	Fostira et al., 2019	F	BRCA2	RECQL4	Breast, Ovarian	Recent
220	Nurmi et al., 2019	F	ATM	FANCM	Breast, Breast	Recent
227	Stradella et al., 2019	F	FH	BARD1	Cutaneous Leiomyoma, Cutaneous Leiomyoma, CRC	Recent
230	Silva-Smith and Sussman, 2018	M	PMS2	BMPR1A	Bladder, CRC	Recent
**232**	Carbajal-Mamani et al., 2020	F	ATM	PALB2	Breast, Breast, **Ovarian**	Recent
234	Fostira et al., 2019	F	PALB2	SLX4	Breast, Ovarian	Recent

**259**	Pedroni et al. 2013	F	BRCA1	MLH1	Breast, Endometrial, Ovarian, **Renal**, Breast	Historical
**261**	Thiffault et al. 2004	F	BRCA2	MSH2	Breast, Breast, **Endometrial**, CRC	Historical
263	Manoukian et al. 2007	F	BRCA2	TP53	Breast, Breast, Soft Tissue Sarcoma	Historical
264	Monnerat et al. 2007	F	BRCA2	TP53	Melanoma, Breast, Ovarian, CRC	Historical
265	Ghataorhe et al. 2007	F	BRCA2	MEN1	Parathyroid, Pancreas	Historical
267	Ghataorhe et al. 2007	M	BRCA2	MEN1	Parathyroid, Breast	Historical
268	Kashiwada et al. 2012	F	APC	FLCN	CRC, CRC	Historical
269	Kilmartin et al. 1996	M	APC	VHL	CRC, Haemangioma, CNS	Historical
**270**	Mastroianno et al. 2011	M	RET	MEN1	Pituitary, **Thyroid**, **Thyroid**, Stomach, Stomach Carcinoid	Historical
**271**	Mastroianno et al. 2011	M	RET	MEN1	Carcinoid, Lipoma, Thyroid, Thyroid, **Angiofibroma,** Stomach	Historical
**274**	Plon et al. 2008	F	PTEN	TP53	CNS, Lipoma, Haemangioma, **Ovarian**, Soft Tissue Sarcoma, CNS	Historical
**275**	Valle et al. 2004	F	PTEN	APC	CRC, Thyroid, Thyroid, **Ovarian**, CNS, Lipoma	Historical
276	Zbuk et al. 2007	F	PTEN	SDHC	Paraganglioma, Breast, Thyroid, Paraganglioma, Uterine	Historical
**277**	Lindor et al. 2012	M	APC	MLH1	CRC, Small Bowel, **Small Bowel**, Skin, Skin, Sabaceous	Historical
278	Soravia et al. 2005	M	APC	MSH2	CRC, CRC, Stomach, Small Bowel, Desmoid	Historical

280	Scheenstra et al. 2003	M	APC	MLH1	CRC, CRC	Historical
281	Puijenbroek et al. 200	F	MSH6	MUTYH	CRC, CRC	Historical
**282**	Pern et al. 2012	F	BRCA1	PALB2	**Uterine**, **CNS**, Breast	Historical
284	Campos et al. 2013	F	BRCA1	NF1	Skin, Breast	Historical
285	Augustyn et al. 2011	F	BRCA1	BRCA2	Ovarian, Ovarian	Historical
287	Bell et al. 2002	F	BRCA1	BRCA2	Breast, Breast	Historical
298	Heidemann et al. 2012	F	BRCA1	BRCA2	Breast, Breast, Ovarian	Historical
301	Heidemann et al. 2012	F	BRCA1	BRCA2	Breast, Breast	Historical
**303**	Heidemann et al. 2012	F	BRCA1	BRCA2	**CRC**, Ovarian	Historical
305	Heidemann et al. 2012	F	BRCA1	BRCA2	**Cervical**, Breast	Historical
306	Leegte et al. 2005	F	BRCA1	BRCA2	Ovarian, Breast	Historical
318	Pilato et al. 2010	F	BRCA1	BRCA2	Breast, Ovarian	Historical
**319**	Smith et al. 2008	F	BRCA1	BRCA2	Breast, **CRC**, Breast	Historical
323	Zuradelli et al. 2010	F	BRCA1	BRCA2	Breast, Ovarian	Historical
325	Zuradelli et al. 2010	F	BRCA1	BRCA2	Breast, Ovarian	Historical
330	Ramus et al. 1997	F	BRCA1	BRCA2	Breast, Ovarian	Historical
333	Leegte et al. 2005; Frank et al. 2002	Unknown	BRCA1	BRCA2	Breast, Breast	Historical
334	Leegte et al. 2005; Frank et al. 2002	Unknown	BRCA1	BRCA2	Breast, Breast	Historical

337	Leegte et al. 2005<br>Frank et al. 2002	Unknown	BRCA1	BRCA2	Breast, Breast	Historical
339	Randall et al. 1998	F	BRCA1	BRCA2	Breast, Ovarian	Historical
**340**	Whitworth et al. 2015	M	FLCN	NF1	**Testicular**, Renal, Phaeochromocytoma, GIST, GIST, Sarcoma, Skin	Historical
**341**	Whitworth et al. 2015	M	FLCN	TP53	**CRC, Oesophageal**, Renal	Historical
343	Whitworth et al. 2015	F	FLCN	MSH2	Renal, CRC	Historical
345	Whitworth et al. 2015	M	MLH1	XPA	CRC, CRC, Skin, Skin	Historical
346	Whitworth et al. 2015	F	BRCA2	NF1	Breast, Melanoma, Skin, Sarcoma	Historical
349	Stolavora et al., 2020	F	POT1	CHEK2	Melanoma, Melanoma, Breast	Recent
**353**	Stolavora et al., 2020	F	TYR	FANCC	Breast, **Melanoma**, **CRC**	Recent
**356**	Stolavora et al., 2020	M	BRCA2	TYRP1	Melanoma, **Non-Hodgkin Lymphoma**	Recent
357	Stolavora et al., 2020	F	BRCA1	ATM	Breast, Ovarian	Recent
**358**	Stolavora et al., 2020	F	ATM	WRN	**Melanoma**, **Melanoma**	Recent
362	Sukumar et al., 2021	F	BRCA1	BRCA2	Breast, Breast	Recent
**366**	Le Duc et al., 2020	M	TP53	RAD51C	Skin, **Sabaceous**	Recent
367	Vietri et al., 2020	F	BRCA1	APC	Polyps, Desmoid, Ovarian	Recent
371	Ramus et al., 1997; Le Page 2020	F	BRCA1	BRCA2	Breast, Ovarian	Recent
372	Ramus et al., 1997; Le Page 2020	F	BRCA1	BRCA2	Breast, Ovarian	Recent
373	Ramus et al., 1997; Le Page 2020	F	BRCA1	BRCA2	Breast, Ovarian	Recent
376	Liede et al., 1998; Le Page 2020	F	BRCA1	BRCA2	Breast, Ovarian	Recent

Supplementary Figure 1. *Workflow for systematic literature review*

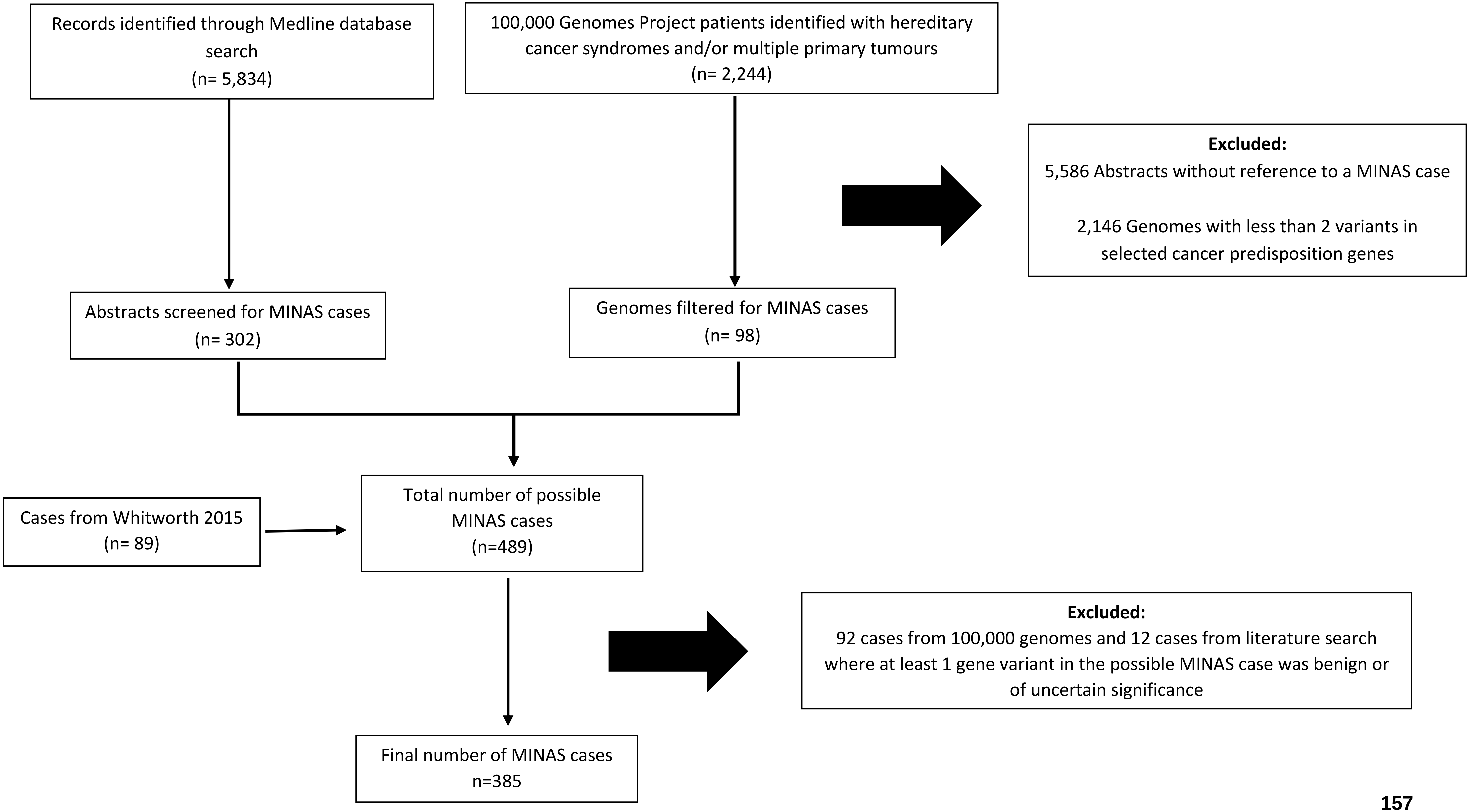

Supplement: Supplementary file 1 — Supplementary Material [file 41431_2021_1013_MOESM1_ESM.pdf]
